# Supplementary material for: The imprinted Igf2-Igf2r axis is critical for matching placental microvasculature expansion to fetal growth
Source: Dev Cell. 2022 Jan 10;57(1):63–79.e8. doi: 10.1016/j.devcel.2021.12.005 (PMC8751640; doi:10.1016/j.devcel.2021.12.005)
Supplement: Document S1. Figures S1–S7 and Tables S4–S7 [file mmc1.pdf]

Developmental Cell, Volume *57*

Supplemental information

The imprinted *Igf2-Igf2r* axis is critical for matching placental microvasculature expansion to fetal growth

Ionel Sandovici, Aikaterini Georgopoulou, Vicente Pérez-García, Antonia Hufnagel, Jorge López-Tello, Brian Y.H. Lam, Samira N. Schiefer, Chelsea Gaudreau, Fátima Santos, Katharina Hoelle, Giles S.H. Yeo, Keith Burling, Moritz Reiterer, Abigail L. Fowden, Graham J. Burton, Cristina M. Branco, Amanda N. Sferruzzi-Perri, and Miguel Constância

SUPPLEMENTAL INFORMATION

FIGURE S1

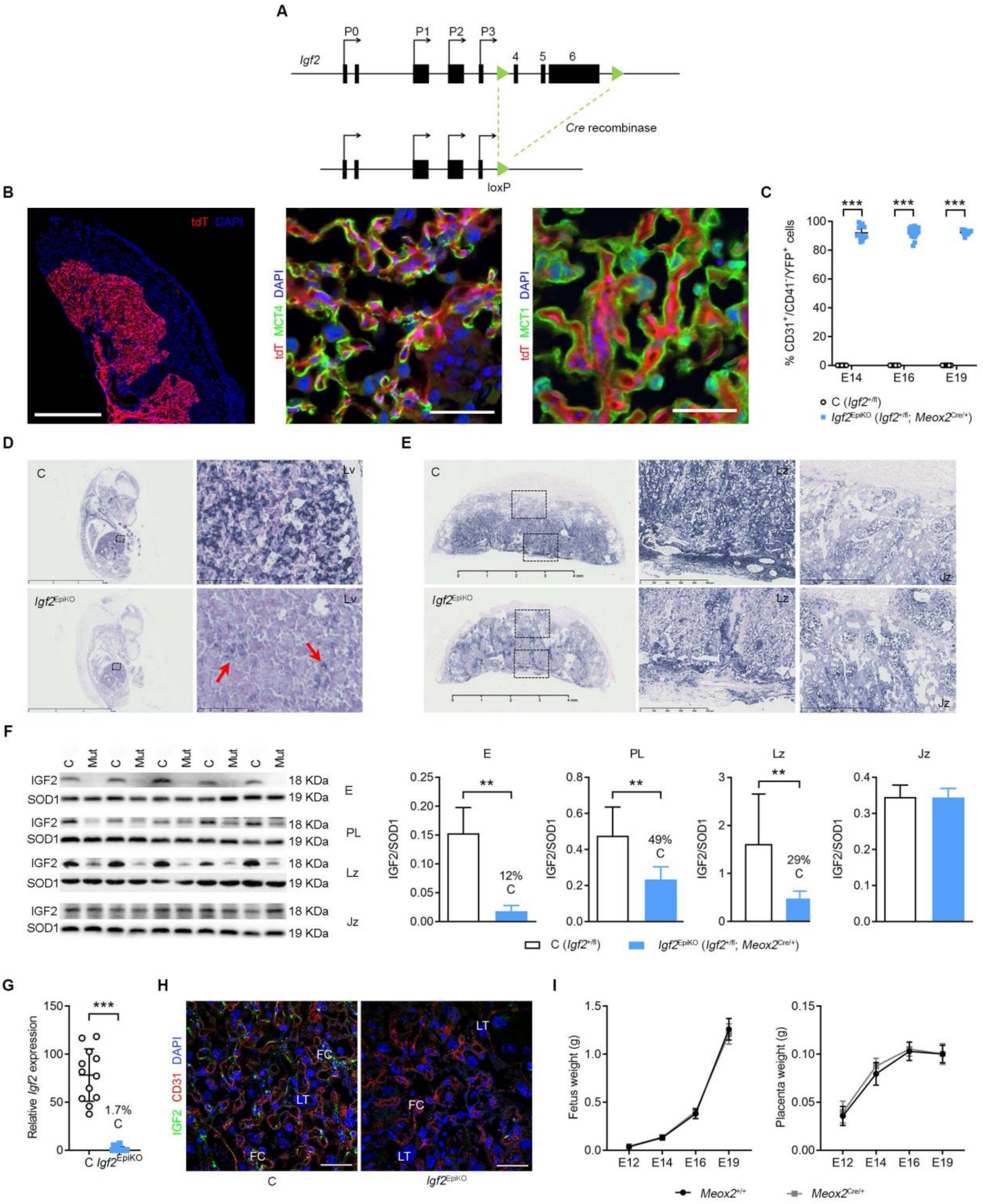

Figure S1. Specificity and efficiency of *Igf2* deletion in fetal tissues and feto-placental endothelial cells by *Meox2*Cre. Related to Figure 2.

(**A**) Schematic representation of the floxed *Igf2* allele. P0-P3 are alternative promoters. Protein-coding exons (4–5), flanked by loxP sites (green triangles), are excised upon Cre-loxP mediated recombination. (**B**) Representative confocal microscopy of a placental frozen section at E16 of

gestation, double transgenic for *Meox2*^{Cre} and Ai9(RCL-tdT) reporter. The *Meox2*^{Cre} is not expressed in the syncytiotrophoblast layers, as demonstrated by the lack of immunostaining overlap between the tomato protein (red) and MCT4 (a marker of the syncytiotrophoblast layer II, facing the feto-placental capillaries) or MCT1 (marker of the syncytiotrophoblast layer I, facing the maternal blood spaces). Scale bars are 1 mm (left panel) and 50 µm (middle and right panels). (**C**) Flow cytometry analysis shows that the majority (>80%) of *Igf2*^{EpiKO} mutant FPEC (CD31^{+}/CD41^{-} cells) express YFP (activated by *Meox2*^{Cre} mediated deletion of the *Rosa26* ^{fl}STOP^{fl}YFP STOP cassette), thus demonstrating good efficiency of *Meox2*-Cre in these cells (n=9–18 per genotype). (**D**) *Igf2* mRNA in situ hybridization (ISH) in E14 control and mutant fetuses. Dark blue indicates *Igf2* mRNA, with nuclei marked in red. Insets illustrate efficient *Igf2* deletion in the liver (Lv); arrows – small pockets of cells with incomplete *Igf2* deletion (mosaic activity of *Meox2*^{Cre}). Scale bars are 6 mm (left) and 100 µm (right). (**E**) *Igf2* mRNA ISH in E14 control and mutant placentae. Insets show reduced *Igf2* mRNA signal in the placental labyrinthine zone (Lz) of mutants, due to its deletion from FPEC, while *Igf2* expression is unchanged in the junctional zone (Jz). Scale bars are 4 mm (left) and 500 µm (right). (**F**) Western blot analysis of pro-IGF2 (18 kDa) in cell lysates from whole fetuses (F), whole placenta (PL) micro-dissected Lz and Jz at E14, and corresponding data quantification shown as graphs (n=5 per genotype). SOD1 (19 kDa) was used as loading control. (**G**) Efficiency of *Igf2* deletion evaluated by qRT-PCR in fluorescence-activated sorted FPEC (n=12 per genotype). (**H**) Representative immunofluorescence confocal microscopy at E16 showing near complete absence of IGF2 protein within CD31+ feto-placental endothelial cells in *Igf2*^{EpiKO} mutants compared to littermate controls. FC – fetal capillaries, LT – labyrinthine trophoblast. Scale bars are 50µm. (**I**) Fetal and placental growth kinetics are not altered in *Meox2*$^{Cre/+}$ carriers (maternal inheritance) (n=8–30 conceptuses per genotype at each developmental stage). For all graphs, data is shown as individual values or averages ± SD; ** $P < 0.01$, *** $P < 0.001$ calculated by two-way ANOVA plus Sidak's multiple comparisons tests in (C) or Mann Whitney tests in (F) and (G).

Figure S2

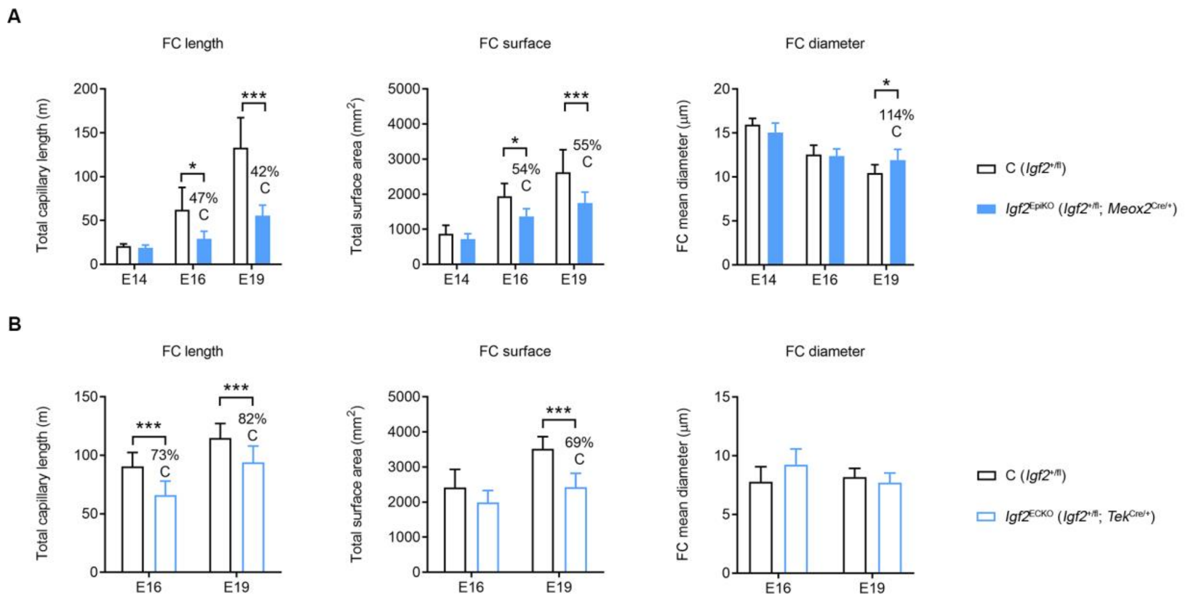

Figure S2. Impact of *Igf2*^{EpiKO} and *Igf2*^{ECKO} deletions on feto-placental capillary expansion during gestation. Related to Figure 2.

(**A**) Parameters of feto-placental capillaries (FC) measured by stereology in *Igf2*^{EpiKO} mutant (*Igf2*$^{+/\mathrm{fl}}$; *Meox2*$^{\mathrm{Cre}/+}$) versus control (C – *Igf2* $^{+/\mathrm{fl}}$) placentae (n=6 per genotype at each developmental stage). (**B**) Parameters of feto-placental capillaries (FC) measured by stereology in *Igf2*^{ECKO} mutant (*Igf2*$^{+/\mathrm{fl}}$; *Tek*$^{\mathrm{Cre}/+}$) versus control (C – *Igf2* $^{+/\mathrm{fl}}$) placentae (n=5–7 per genotype at each developmental stage). For all graphs, data is shown as averages ± SD; * $P < 0.05$, *** $P < 0.001$ calculated by two-way ANOVA plus Sidak's multiple comparisons tests.

Figure S3

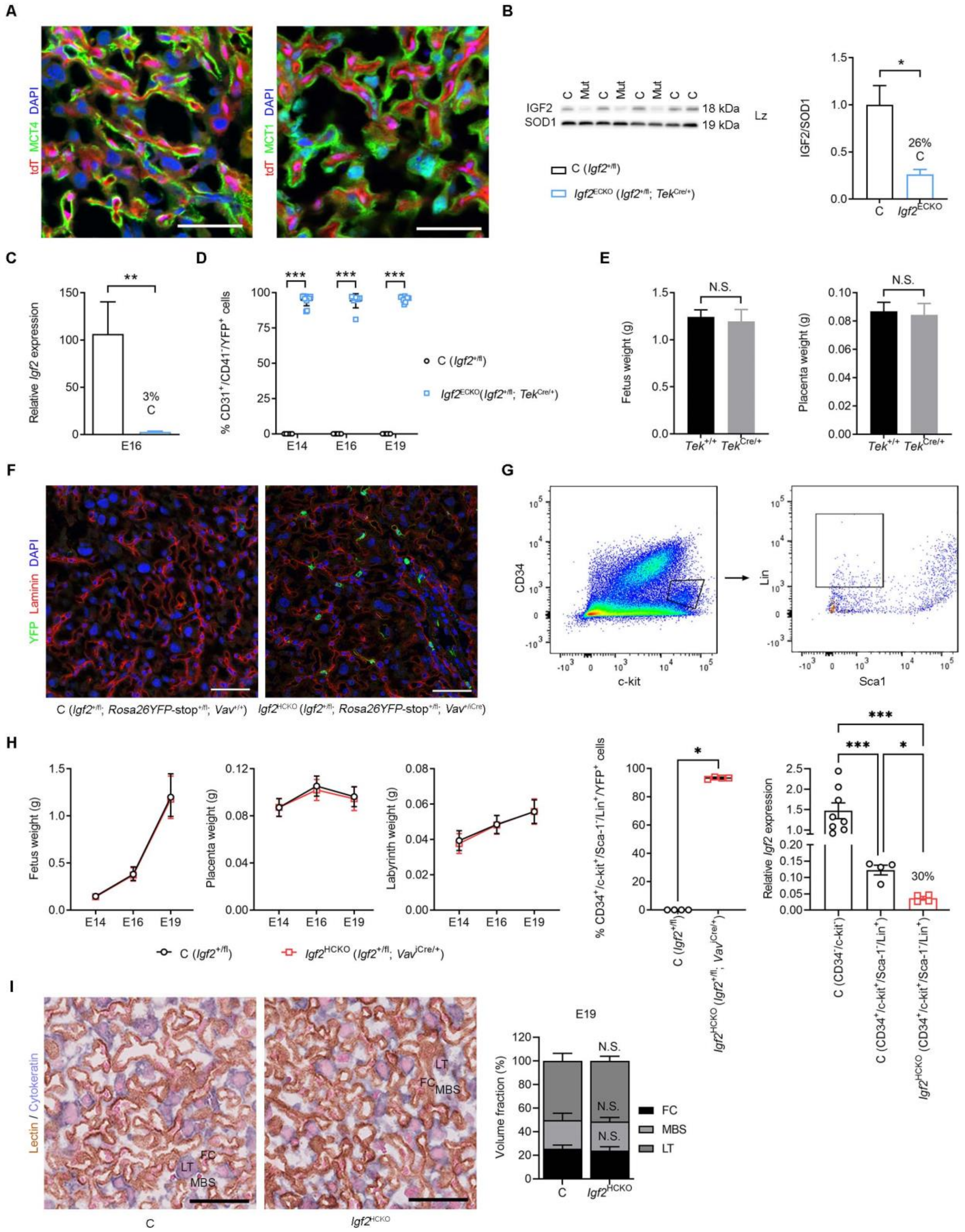

Figure S3. Specificity and efficiency of *Igf2* deletion in the endothelium by *Tek*^{Cre} and in the hematopoietic lineage by *Vav*^{iCre}. Related to Figure 2.

(**A**) Representative confocal microscopy of frozen placental sections from a double transgenic for *TeK*^{Cre} and Ai9(RCL-tdT) reporter at E16 of gestation. The *TeK*^{Cre} is not expressed in the syncytiotrophoblast layers, as demonstrated by the lack of immunostaining overlap between the

tomato protein (red) and MCT4 (Syn-TII layer) or MCT1 (Syn-TI layer). Scale bars are 50 µm. (**B**) Western blot analysis of pro-IGF2 (18 kDa) in cell lysates from Lz micro-dissected at E16 and corresponding data quantification (n=3 per genotype). SOD1 (19 kDa) was used as internal control for loading. (**C**) Efficiency of *Igf2* deletion evaluated by qRT-PCR in fluorescence-activated sorted FPEC (n=5–7 per genotype). (**D**) Flow cytometry analysis shows that the majority (>80%) of *Igf2*^{ECKO} mutant FPEC express YFP, thus demonstrating good efficiency of *TeK*^{Cre} in these cells (n=5–11 per genotype). (**E**) Fetal and placental growth kinetics are not altered in *TeK*$^{Cre/+}$ carriers (maternal inheritance) at E19 (n=13–15 conceptuses per genotype from 4 independent litters). (**F**) Representative double immunostainings for laminin (marking feto-placental capillaries) and YFP in E19 *Igf2*^{HCKO} mutant and littermate control (C) placentae. YFP expression is activated in cells of hematopoietic lineage by deletion of the floxed STOP cassette within the *Rosa26*^{fl}STOP^{fl}YFP reporter construct. Scale bars are 50µm. (**G**) Gating strategy used to isolate cells of hematopoietic lineage from E13 placentae by FACS (top) and efficiency of *Igf2* deletion evaluated by qRT-PCR (bottom). The CD34^{+}/c-kit^{+} population gated in the top left panel was subsequently used to isolate Lin^{+}/Sca1^{-} hematopoietic cells (top right panel). Bottom left: more than 90% of CD34^{+}/c-kit^{+}/Lin^{+}/Sca1^{-} cells are positive for YFP, indicating efficient activity of *Vav*^{iCre} in the hematopoietic cells. Bottom right: measurement of mRNA levels at E13 by qRT-PCR indicates overall low levels of *Igf2* expression in the hematopoietic lineage (approximately one order of magnitude lower than in CD34^{-}/c-kit^{-} cells), as well as efficient deletion of *Igf2* in hematopoietic cells of *Igf2*^{HCKO} mutants compared to littermate controls (n=4–8/group). (**H**) Fetal, placental, and Lz growth kinetics are not altered in *Igf2*^{HCKO} mutants compared to controls (n=11–25 conceptuses from n=5–6 litters for each developmental stage). (**I**) Left: representative double immunostaining for lectin (brown, marking feto-placental capillaries) and cytokeratin (blue, marking the labyrinthine trophoblast) in E19 placental sections of *Igf2*^{HCKO} mutants and littermate controls. Right: the relative volume fractions occupied by the three main labyrinthine constituents (FC – fetal capillaries, MBS – maternal blood spaces and LT – labyrinthine trophoblast) are not altered in *Igf2*^{HCKO} mutants compared to controls at E19 (n=4–7 samples/group). For all graphs, data is shown as individual values or averages ± SD in (B), (D), (E), (G) – bottom left and (I), SEM in (C) and (G) – bottom right, or 95% confidence intervals (95%CI) in (H); N.S. – statistically non-significant; * $P < 0.05$, ** $P < 0.01$ and *** $P < 0.001$ calculated by Mann Whitney tests in (B), (C), (E) and (G) – bottom left, two-way ANOVA plus Sidak's multiple comparisons tests in (D) and (I), Brown-Forsythe and Welch ANOVA tests in (G) – bottom right and mixed effects model in (H).

Figure S4

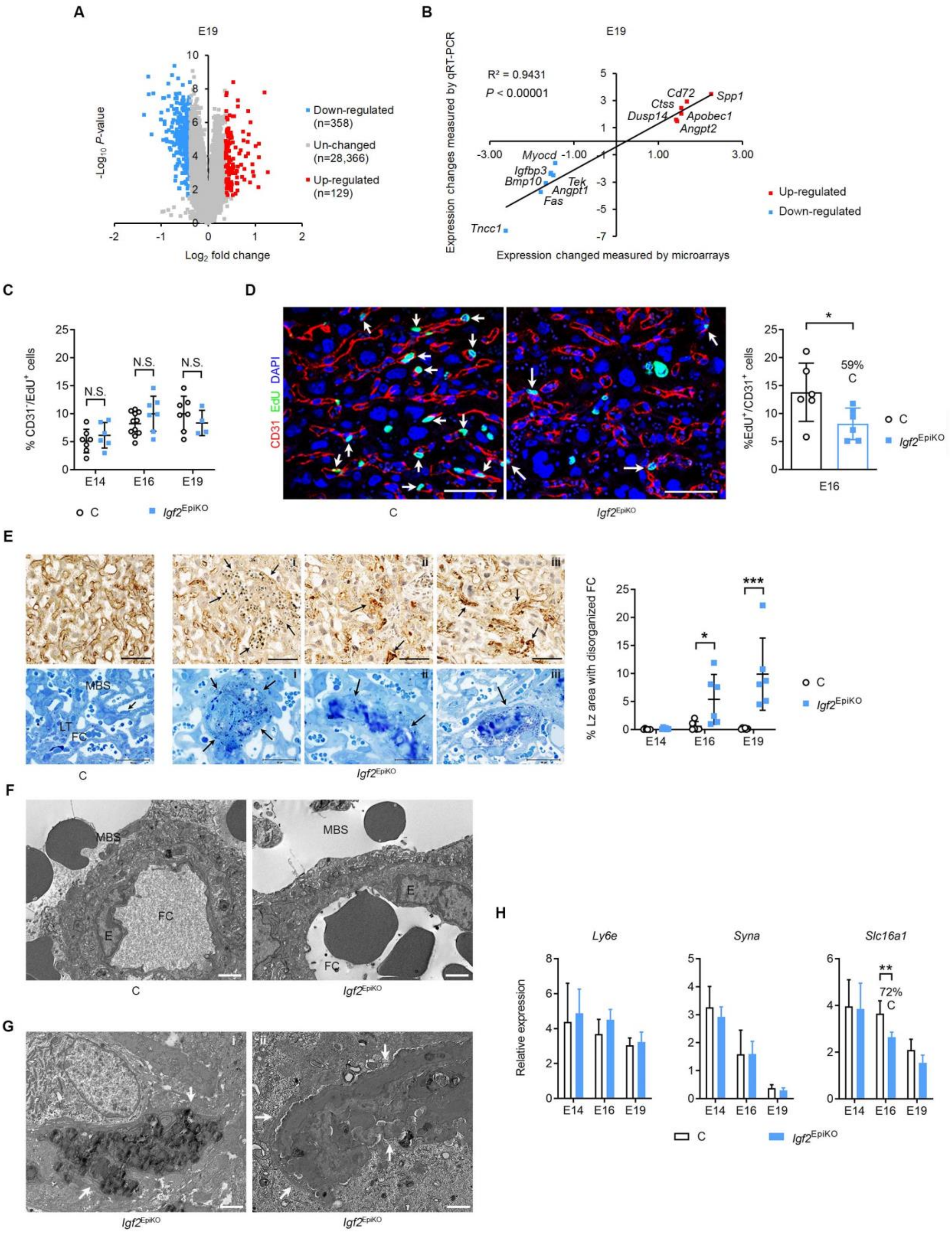

Figure S4. Fetus-derived *Igf2* deletion (*Igf2*^{EpiKO}) alters gene expression in placental Lz and FPEC survival and proliferation. Related to Figure 3.

(**A**) Volcano plot depicting differentially expressed genes (DEG) identified in E19 Lz by expression microarray analysis (n=6 samples per genotype, all from male conceptuses). (**B**) Biological validation using qRT-PCR for 13 DEGs (n=6–7 samples per genotype), normalized against three housekeeping

genes (*Sdha*, *Gapdh* and *Pmm1*). (**C**) The reduction in proliferation seen in FEPC (Fig. 3e) is not observed in non-endothelial cells from Lz measured by flow cytometry analysis after EdU injections (16 hours exposure; n=4–11 per group). (**D**) Representative confocal microscopy image of FPEC proliferation in control (panel i) versus mutant (panel ii) E16 placentae by immunofluorescent staining for CD31 combined with Click-iT EdU imaging. Arrows point towards FPEC nuclei that incorporated EdU *in vivo* during the 16 hours exposure to the thymidine analogue. Scale bar is 50 µm. The accompanying graph shows data quantification based on counting between 250 to 600 FPEC per sample (n=6 placentae/genotype). **(E)** Top row: CD31 staining in control (C) and *Igf2*^{EpiKO} mutant E16 Lz illustrating abnormally large FCs lacking endothelial cells (i) or obstructed capillaries surrounded by fragmented and disorganized FPEC (ii, iii). Scale bars are 50µm. Bottom row: methylene blue-stained E16 Lz resin sections (arrows indicate a FPEC in C and thrombotic FC in mutants: i-iii). Scale bars are 30µm. The graph on the right side shows the quantification of percentage of Lz areas with disorganized pattern of FC. Measurement of total Lz and abnormal Lz areas was performed using HALO image analysis software (n=6 samples per genotype at each developmental stage). (**F**) Representative transmission electron microscopy (TEM) micrographs showing intact feto-maternal barriers separating fetal capillaries (FC) from maternal blood spaces (MBS) in both controls (C) and *Igf2*^{EpiKO} mutants, at E16. Scale bars are 2µm (n=3 biological replicates per group with 6-15 micrographs/sample). (**G**) TEM micrographs from E16 *Igf2*^{EpiKO} mutants. White arrows point towards an apoptotic endothelial cell (panel i) and a blocked/thrombotic capillary with apoptotic endothelial lining (panel ii). In both cases the overlying trophoblast is intact. Scale bars are 2µm. (**H**) qRT-PCR analysis of genes expressed in Syn-TI in micro-dissected Lz in mutants versus controls (n=6–8 samples per group for each developmental time point). For all graphs, data is presented as averages or individual values ± SD; N.S. – non-significant, * $P < 0.05$, ** $P < 0.01$, *** $P < 0.001$ by two-way ANOVA plus Sidak's multiple comparisons tests in (C), (D) and (H) or Mann Whitney test in (E).

Figure S5

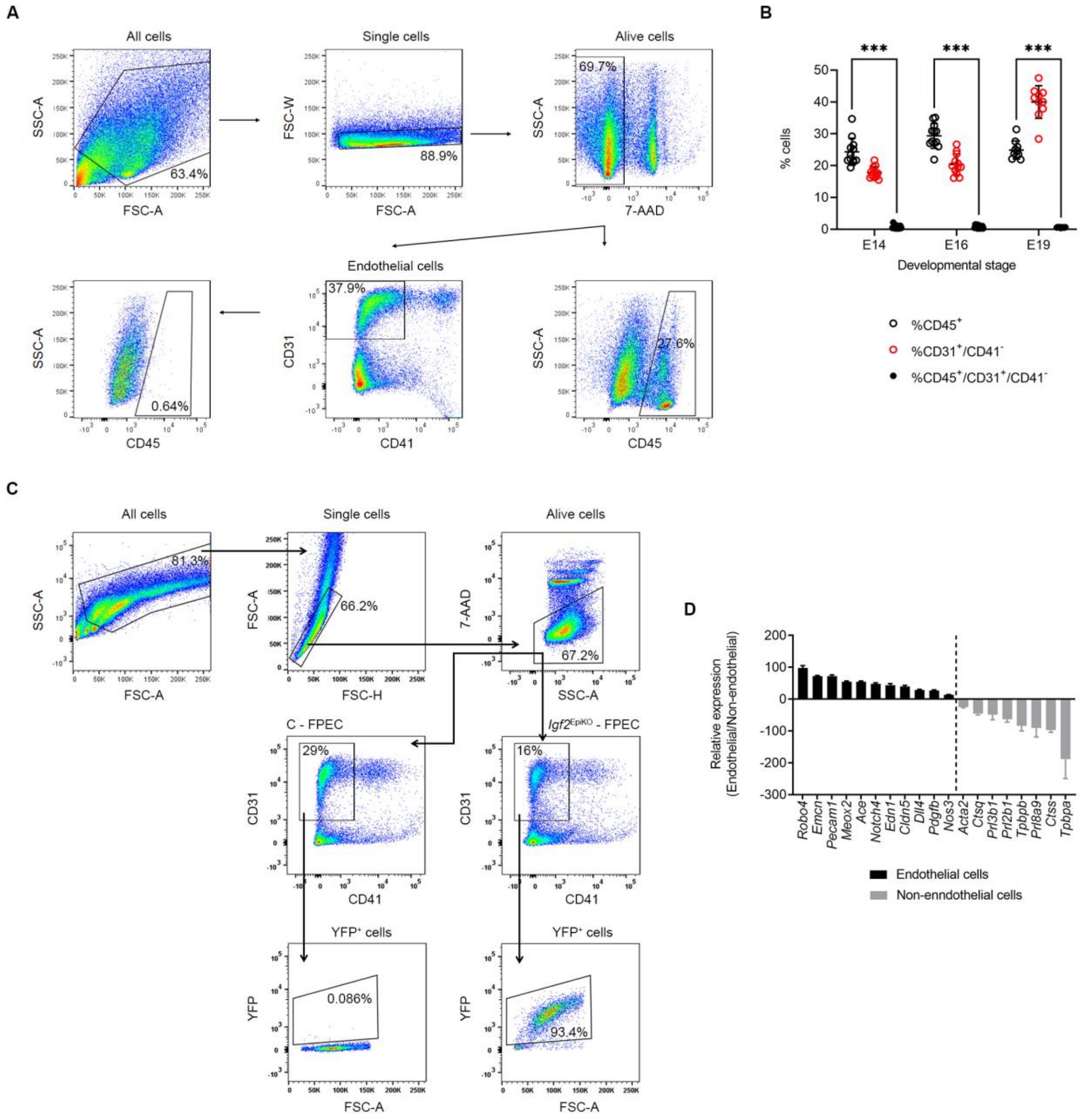

Figure S5. Experimental design used for FPEC analysis by flow cytometry and FPEC isolation by FACS. Related to Figures 4 and 5.

(**A**) Gating strategy used for flow cytometry analysis of CD45 expression (marker of all differentiated hematopoietic cells, except erythrocytes and plasma cells) within FPEC (defined as $CD31^+/CD41^-$ cells). The example shown is from an E19 sample. (**B**) Statistical analysis of CD45+, $CD31^+/CD41^-$ (FPEC) and $CD45^+/CD31^+/CD41^-$ cells at E14, E16 and E19. At all three developmental stages, the proportion of $CD45^+$ within the FPEC ($CD31^+/CD41^-$) is very low. The graph show individual data points with averages and SD (n=10-12 samples from two litters at each developmental stage). *** $p < 0.001$ by two-way ANOVA with Tuckey's multiple comparisons test. (**C**) Gating strategy used for FPEC and YFP analysis by flow cytometry and FPEC isolation by FACS. Mutant FPEC are also positive for YFP (activated by Cre mediated deletion of the *Rosa26* ^{fl}STOP^{fl}YFP STOP cassette), thus providing an internal control for Cre efficiency in each biological sample. (**D**) RNA-seq analysis of marker genes expressed in FPEC or non-endothelial cells isolated by FACS from E16 control Lz. The graph shows the relative enrichment in

FPEC of known markers of endothelial cells (black) and depletion of marker genes expressed by other cell types found in the Lz (grey): pericytes (*Acta2*), sinusoidal trophoblast giant cells (*Ctsq*, *Prl2b1*, *Prl8a9*), parietal trophoblast giant cells (*Prl3b1*) or spongiotrophoblast cells (*Tpbpa*, *Tpbpb*).

Figure S6

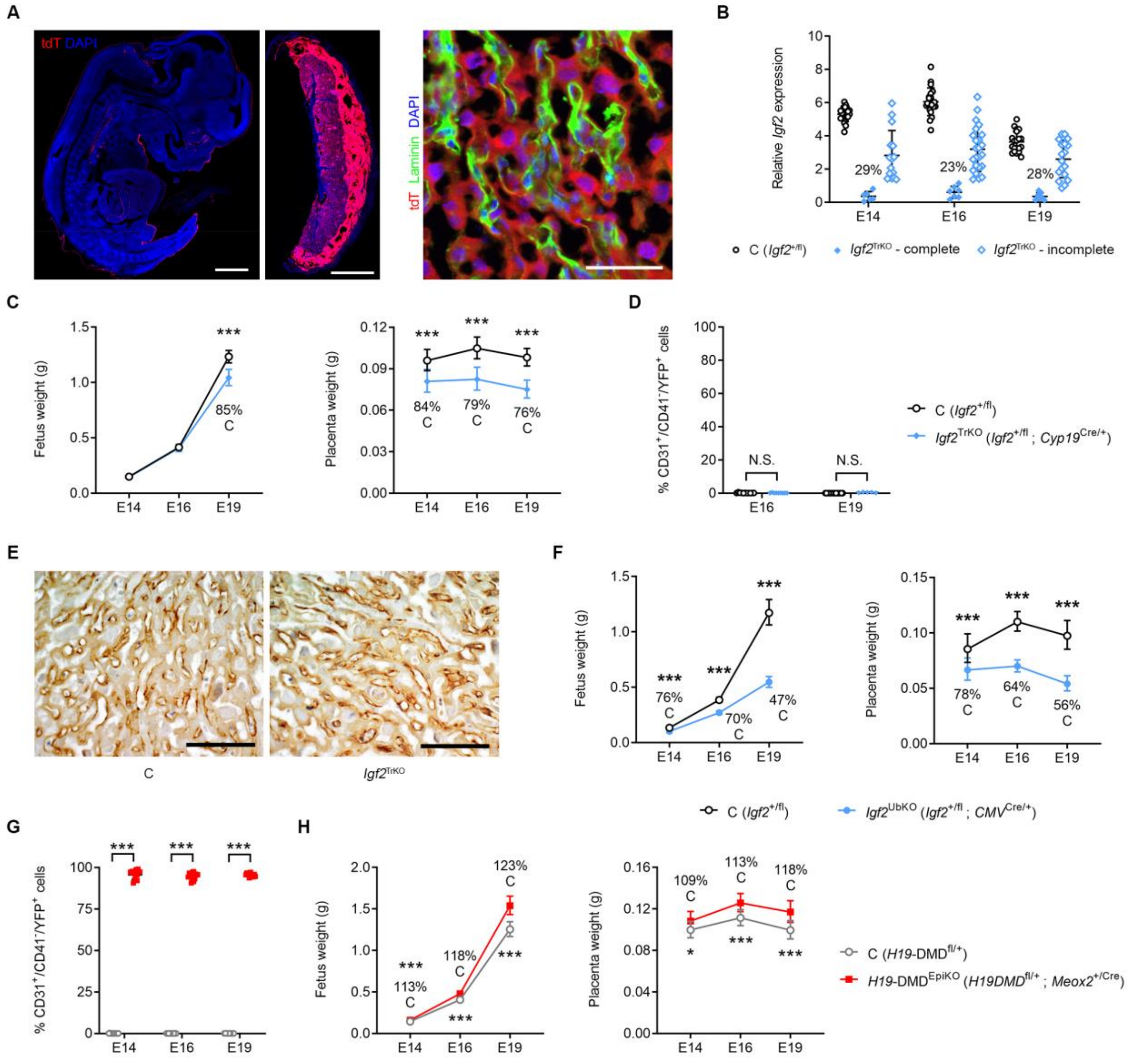

Figure S6. Specificity and efficiency of *Igf2*^{TrKO}, *Igf2*^{UbKO} and *H19*-DMD^{EpiKO} deletions. Related to Figure 4.

(**A**) Representative confocal microscopy on frozen sections from a double transgenic Ai9(RCL-tdT), *Cyp19*^{Cre} fetus and corresponding placenta, at E16, demonstrating high Cre activity (red) in placenta and weak activity in embryonic skin and eye lenses. Right panel: *Cyp19*^{Cre} is only active in the trophoblast cells in Lz, as demonstrated by lack of overlapping between the tomato protein (red) and laminin (green) expressed in FPEC. Scale bars are 1 mm (left and middle panel) or 50 µm (right panel). (**B**) Efficiency of *Igf2* deletion by *Cyp19*^{Cre} in *Igf2*^{TrKO} mutants (*Igf2*$^{+/fl}$; *Cyp19*$^{Cre/+}$) versus controls (*Igf2*$^{+/fl}$) evaluated using qRT-PCR in micro-dissected placental Jz layer (n=20–31 samples per genotype). Only 23-29% of all *Igf2*^{TrKO} mutants have high levels of deletion (>80%). (**C**) Placenta growth restriction precedes fetal growth restriction in *Igf2*^{TrKO} mutants (n=4–9 litters at each developmental stage; only mutants with >80% deletion were included in this analysis). (**D**) Flow cytometry analysis showing that *Cyp19*^{Cre} is not expressed in FPEC (note lack of YFP expression in *Igf2*^{TrKO} mutants) (n=6–21 per genotype). (**E**) Representative CD31 immunostainings in E16 control and *Igf2*^{TrKO} Lz (scale bars are 100µm) showing no impact of the deletion on FPEC numbers. (**F**) Severe fetal and placental growth

restriction in *Igf2*^{UbKO} (*Igf2*$^{+/fl}$; *CMV*$^{Cre/+}$) mutants (n=3-8 litters at each developmental stage). (**G**) Flow cytometry analysis shows that the majority (>80%) of *H19*-DMD^{EpiKO} (*Meox2*$^{+/Cre}$; *H19*-DMD$^{fl/+}$) mutant FPEC express YFP, demonstrating good efficiency of *Meox2*^{Cre} in these cells (n=9–15 per genotype). (**H**) Fetal and placental overgrowth in *H19*-DMD^{EpiKO} mutants (n=3-4 litters at each developmental stage). For all graphs, data is shown as averages or individual values ± SD in (B), (D) and (G) or 95% confidence intervals (95%CI) in (C), (F) and (H). N.S. – non-significant, * $P < 0.05$; ** $P < 0.01$; *** $P < 0.001$ calculated by two-way ANOVA plus Sidak's multiple comparisons tests in (B), (D), and (G) and mixed effects model in (C), (F) and (H).

Figure S7

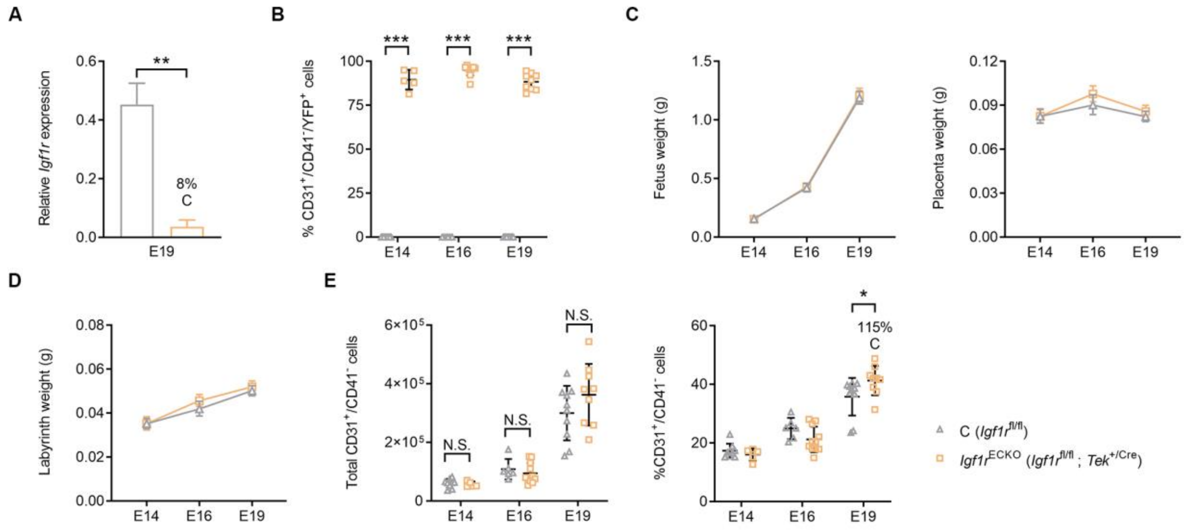

Figure S7. Conditional deletion of *Igf1r* from endothelium using *Tek*^{Cre}. Related to Figure 7.

(**A**) qRT-PCR analysis of *Igf1r* mRNA levels in primary FPEC isolated by FACS from E19 Lz of *Igf1r*^{ECKO} (*Igf1r*$^{fl/fl}$; *Tek*$^{+/Cre}$) mutants versus *Igf1r*$^{fl/fl}$ controls. (**B**) Flow cytometry analysis showing that the majority (>80%) of *Igf1r*^{ECKO} mutant FPEC express YFP, demonstrating good efficiency of *Tek2*^{Cre} in these samples (n=5–11 per genotype). Fetal, placental (**C**) and Lz (**D**) growth kinetics are not altered in *Igf1r*^{ECKO} mutants compared to controls (n=6–18 conceptuses from n=3-7 litters for each developmental stage). (**E**) Total numbers and proportions of FPEC/Lz measured by flow cytometry (n=5–11 per genotype). For all graphs, data is shown as individual values or averages ± SD in (A), (B) and (E) or 95%CI in (C) and (D); N.S. – non-significant, * $P < 0.05$, ** $P < 0.01$, *** $P < 0.001$ by Mann-Whitney tests in (A) or two-way ANOVA plus Sidak's multiple comparisons tests in (B) and (E) or mixed effects model in (C) and (D).

Table S4. Angiostatic and pro-angiogenic factors produced by feto-placental endothelial cells under the control of fetus-derived IGF2. Related to Figure 5.

Protein	Expression change	Function (cellular compartment)	Role in angiogenesis	PMID
CXCL10	Up-regulated	Cytokine (extracellular space)	angiostatic	7537965, 7540647, 8611715, 9064358, 10914483
IL15	Up-regulated	Cytokine (extracellular space)	angiostatic	28379958
THBS1	Up-regulated		angiostatic	22553494
ADAMTS1	Up-regulated	Peptidase (extracellular space)	angiostatic	12716911, 12814950, 17082774, 22776012
APCDD1	Up-regulated	Membrane-bound glycoprotein (cellular membrane)	angiostatic	29154126
IFIT2	Up-regulated	Interferon-induced protein (cytoplasm)	angiostatic	26515391
IFI16	Up-regulated	Transcription regulator (nucleus)	angiostatic	14729471, 21488755
CCL2	Up-regulated	Cytokine (extracellular space)	pro-angiogenic/ angiostatic if prolonged expression	15516694, 16888027, 23329645
EGR1	Up-regulated	Transcription regulator (nucleus)	pro-angiogenic/ angiostatic if prolonged expression	10339488, 12872165, 16818645, 27041221
KLF4	Up-regulated	Transcription regulator (nucleus)	pro-angiogenic/ angiostatic if prolonged expression	24599951, 27431648, 26823670
GDF15	Up-regulated	Growth factor (extracellular space)	pro-angiogenic	21773947, 28831101
HBEGF	Up-regulated	Growth factor (extracellular space)	pro-angiogenic	15289334, 18925469
SERPINE1	Up-regulated	Protease inhibitor (extracellular space)	pro-angiogenic	26180080
PLAT	Up-regulated	Peptidase (extracellular space)	pro-angiogenic	24601228
ISG20	Up-regulated	Exonuclease (nucleus)	pro-angiogenic	29195126
HEY2	Down-regulated	Transcription regulator (nucleus)	pro-angiogenic	15107403, 16219802, 22421041

Table S5: Mouse strains and crosses. Related to STAR Methods.

Paternal genotype	Maternal genotype	Offspring/embryo genotypes	Related to Figure
Igf2$^{+/+}$	*Igf2*$^{+/+}$	*Igf2*$^{+/+}$	Figure 1<br>Figure 2I<br>Figure 6<br>Figures S5A, S5B, S5D
Igf2$^{fl/fl}$;<br>*Rosa26YFP*-stop$^{fl/fl}$	*Meox2*$^{+/Cre}$	C (*Igf2*$^{+/fl}$;<br>*Rosa26YFP*-stop$^{fl/fl}$;<br>*Meox2*$^{+/+}$)	Figures 2B, 2C, 2D, 2J<br>Figure 3<br>Figure 4A<br>Figures 5A, 5C<br>Figures S1C, S1D, S1E, S1F, S1G, S1H<br>Figure S2A<br>Figure S4<br>Figure S5C
		Igf2^{EpiKO} (*Igf2*$^{+/fl}$;<br>*Rosa26YFP*-stop$^{+/fl}$;<br>Meox2$^{Cre/+}$)	
Igf2$^{fl/fl}$;<br>*Rosa26YFP*-stop$^{fl/fl}$	*Tek*$^{+/Cre}$	C (*Igf2*$^{+/fl}$;<br>*Rosa26YFP*-stop$^{fl/fl}$;<br>*Tek*$^{+/+}$)	Figures 2F, 2G, 2H, 2J<br>Figure 4B<br>Figure 5D<br>Figure S2B<br>Figures S3B, S3C, S3D
		Igf2^{ECKO} (*Igf2*$^{+/fl}$;<br>*Rosa26YFP*-stop$^{+/fl}$;<br>Tek$^{Cre/+}$)	
Igf2$^{fl/fl}$;<br>*Rosa26YFP*-stop$^{fl/fl}$	*Vav*$^{+/iCre}$	C (*Igf2*$^{+/fl}$;<br>*Rosa26YFP*-stop$^{fl/fl}$;<br>*Vav*$^{+/+}$)	Figures S3F, S3G, S3H, S3I
		Igf2^{HCKO} (*Igf2*$^{+/fl}$;<br>*Rosa26YFP*-stop$^{+/fl}$;<br>*Vav*$^{iCre/+}$)	
Igf2$^{+/+}$	*Meox2*$^{+/Cre}$	*Igf2*$^{+/+}$; *Meox2*$^{Cre/+}$	Figure S1I
		Igf2$^{+/+}$; *Meox2*$^{+/+}$	
Ai9(RCL-tdT)$^{fl/fl}$	*Meox2*$^{+/Cre}$	*Ai9(RCL-tdT)*$^{+/fl}$; *Meox2*$^{Cre/+}$	Figure S1B
		Ai9(RCL-tdT)$^{+/fl}$; *Meox2*$^{+/+}$	
Ai9(RCL-tdT)$^{fl/fl}$	*Tek*$^{+/Cre}$	*Ai9(RCL-tdT)*$^{+/fl}$; *Tek*$^{Cre/+}$	Figures 2E, S3A
		Ai9(RCL-tdT)$^{+/fl}$; *Tek*$^{+/+}$	
Igf2$^{+/+}$	*Tek*$^{+/Cre}$	*Igf2*$^{+/+}$; *Tek*$^{Cre/+}$	Figure S3E
		Igf2$^{+/+}$; *Tek*$^{+/+}$	
Igf2$^{fl/fl}$;<br>*Rosa26YFP*-stop$^{fl/fl}$	*Cyp19*$^{+/Cre}$	C (*Igf2*$^{+/fl}$;<br>*Rosa26YFP*-stop$^{+/fl}$<br>*Cyp19*$^{+/+}$)	Figure 4C<br>Figures S6B, S6C, S6D, S6E
		Igf2^{TrKO} (*Igf2*$^{+/fl}$;<br>*Rosa26YFP*-stop$^{+/fl}$<br>*Cyp19*$^{Cre/+}$)	
Ai9(RCL-tdT)$^{fl/fl}$	*Cyp19*$^{+/Cre}$	*Ai9(RCL-tdT)*$^{+/fl}$; *Cyp19*$^{Cre/+}$	Figure S6A
		Ai9(RCL-tdT)$^{+/fl}$; *Cyp19*$^{+/+}$	
Igf2$^{fl/fl}$;<br>*Rosa26YFP*-stop$^{fl/fl}$	*CMV*$^{+/Cre}$	C (*Igf2*$^{+/fl}$;<br>*Rosa26YFP*-stop$^{+/fl}$<br>*CMV*$^{+/+}$)	Figure 4D<br>Figure S6F
		Igf2^{UbKO} (*Igf2*$^{+/fl}$;	

		Rosa26YFP-stop$^{+/fl}$<br>*CMV*$^{Cre/+}$)	
Meox2$^{+/Cre}$	*H19-DMD*$^{fl/fl}$;<br>*Rosa26YFP*-stop$^{fl/fl}$	C (*H19-DMD*$^{fl/+}$; *Rosa26YFP*-stop$^{fl/+}$; *Meox2*$^{+/+}$)	Figure 4E<br>Figures S6G, S6H
		H19-DMD^{EpiKO} (*H19-DMD*$^{fl/+}$; *Rosa26YFP*-stop$^{fl/+}$; *Meox2*$^{+/Cre}$)	
Tek$^{+/Cre}$	*Igf2r*$^{fl/fl}$;<br>*Rosa26YFP*-stop$^{fl/fl}$	C (*Igf2r*$^{fl/+}$;<br>*Rosa26YFP*-stop$^{fl/+}$; *Tek*$^{+/+}$)	Figures 7A, 7B, 7C, 7D, 7E, 7F, 7G
		Igf2r^{ECKO} (*Igf2r*$^{fl/+}$;<br>*Rosa26YFP*-stop$^{fl/+}$; *Tek*$^{+/Cre}$)	
Igf1r$^{+/fl}$;<br>*Rosa26YFP*-stop$^{+/fl}$;<br>*Tek*$^{Cre/+}$	*Igf1r*$^{fl/fl}$;<br>*Rosa26YFP*-stop$^{fl/fl}$	C (*Igf1r*$^{fl/fl}$;<br>*Rosa26YFP*-stop$^{fl/fl\ or\ +/fl}$;<br>*Tek*$^{+/+}$)	Figure S7
		Igf1r^{ECKO} (*Igf1r*$^{fl/fl}$;<br>*Rosa26YFP*-stop$^{fl/fl\ or\ +/fl}$;<br>*Tek*$^{+/Cre}$)	
		Het deletions (*Igf1r*$^{+/fl}$;<br>*Rosa26YFP*-stop$^{fl/fl\ or\ +/fl}$;<br>*Tek*$^{+/Cre}$) – not used	

Table S6. Primers used for genotyping or qRT-PCR. Related to STAR Methods.

Primers used for genotyping by PCR					
**Strain**	**Primer**	**Sequence**	**Primer**	**Sequence**	**Amplicon (bp)**
Igf2$^{fl/fl}$	F	TTACAGTTCAAAGCCACCACG	RW<br>RD	GCCAAAGAGATGAGAAGCACC<br>GCCAAACACAGTAAAAAGAAATGC	WT: 324<br>fl: 449<br>del: 384
Rosa26 ^{fl}*STOP*^{fl}*YFP*	F	TGTTATCAGTAAGGGAGCT	R-WT<br>R-fl	CACACCAGGTTAGCCTTTA<br>AAGACCGCGAAGAGTTTGT	WT: 239<br>fl: 301
Meox2-Cre	F	GGACCACCTTCTTTTGGCTTC	R-WT<br>R-Cre	AAGATGTGGAGAGTACGGGGTAG<br>CAGATCCTCCTCAGAAATCAGC	WT: 410<br>Cre: 311
Tek-Cre	F	TGTAAACAAGAGCGAGTGGA	R-WT<br>R-Cre	AGAGAATGGCGAGAAGTCAC<br>TGAGTGAACGAACCTGGTCG	WT: 240<br>Cre: 610
Vav-iCre	F-WT<br>F-Vav	ATGTCTCCAATCCTTGAACACTG<br>GACTACCTCCTGTACCTGCAAG	R-WT<br>R-Vav	GCAGTGGGAGAAATCAGAACC<br>ACTCTGATTCTGGCAATTTCGG	WT: 254<br>Cre: 329
Cyp19-Cre	F	GACCTTGCTGAGATTAGATC	R	AGAGAGAAGCATGTTTAGCTGG	Cre: 545
CMV-Cre	F	CGAGTGATGAGGTTCGCAAG	R	TGAGTGAACGAACCTGGTCG	Cre: 390
H19-DMD$^{fl/fl}$	F	CAGGCCTGTCCTCACCTGAAC	R	GCCAGCTTGCCTTGGCAACCCCTT	WT: 387<br>fl: 520
Igf2r$^{fl/fl}$	F	CCTTCCCTCCAGGCCGTTAC	R	GGTGAGGTCTCCATCTGAGTACC	WT: 225<br>fl: 259
Igf1r$^{fl/fl}$	F	CTTCCCAGCTTGCTACTCTAGG	R	CAGGCTTGCAATGAGACATGGG	WT: 124<br>fl: 220
**Primers used for qRT-PCR**					
**Gene**	**Primer**	**Sequence**	**Primer**	**Sequence**	**Amplicon (bp)**
Igf2	F	AGTCCGAGAGGGACGTGTCTA	R	CGGACTGTCTCCAGGTGTCAT	102
Angpt1	F	GAAGCAACTTCTCAACAGACA	R	TTCTTTGTGTTTTCCCTCCATT	100
Angpt2	F	CTTCTACCTCGCTGGTGAAGAG	R	GCTAAAATCACTTCCTGGTTGG	106
Tek	F	GGAGTGGAGTGAAGAACTAGG	R	GTGGAGTCAGTGATGTTGGAGA	93
Fas	F	CTGCAGACATGCTGTGGATCT	R	GCCTCCTCAGCTTTAAACTCTC	114
Ctss	F	AGAGACCCTACCCTGGACTACC	R	GATTCTTTTCCCAGATGAGACG	109
Spp1	F	ACCATGAGATTGGCAGTGATTT	R	GAGCTGCCAGAATCAGTCACTT	83
Tnnc1	F	GATCTCTTCCGCATGTTTGAC	R	TCAATGTCATCTTCCGTAATGG	107
Myocd	F	ATTCCTGTGCACACTGCTGTAA	R	GAGCTTCTTCACCTTTGGTTTG	96
Apobec1	F	GCACACCTGAGGAAACAAAGTC	R	CAGAGTGGGATCAACAGCTACA	134
Cd72	F	CCAAGGAGAACCTGAAAACTGA	R	GCACCTTTCCTGATATGGAATC	146

Dusp14	F	CTCCCTGGAAATCCTTAGCAC	R	ACCTCTGGAGCTCATGAAGATG	133
Bmp10	F	CTCTACAACAAATTCGCCACAG	R	GAGCCCATTAAAAGTGACTGGT	108
Igfbp3	F	CAGGCAGCCTAAGCACCTAC	R	GGAACTTGGAATCGGTCACTC	135
Adgre1	F	TAGCTGCTCTTCTGATACCCTC	R	CCAACATTCATCTTGTCCCCTC	145
Gcm1	F	CCGCAAGATTTACCTGAGACC	R	GAATAAGCTTCAGGGGTCCATT	98
Syna	F	AGCCCTCTCTGGACAATATTCA	R	CAAGGTGGGAGAAGATATTTGG	89
Synb	F	CAGCTGACACCCTCATTAAACA	R	ATCCAGAAATGGGAATGAAGTG	122
Slc16a1	F	TCGCAGCTTCTTTCTGTAACAC	R	TCATAGTCAGAGCTGGGTTCAA	102
Slc16a3	F	TGCAGAAGCATTATCCAGATCTAC	R	GTATCGATTGAGCATGATGAGG	99
Ly6e	F	ACATGAGAGTCTTCCTGCCTGT	R	TTCTGATCGGTACATGAGAAGC	91
Adamts1	F	CAAAGGACAGGTGCAAGCTC	R	TTGCACACAGACAGAGGTAGAG	119
Cxcl10	F	CGTCATTTTCTGCCTCATCCTG	R	TGATTTCAAGCTTCCCTATGGC	134
Thbs1	F	ATGTACCCATCCAGAGCATCTT	R	GGTTCCAAAGACAAACCTCACA	125
Edn1	F	GACATCATCTGGGTCAACACTC	R	AAGTCTTTCAAGGAACGCTTGG	86
Iigp1	F	ATGATTTGCCCTCCAGCTTTAC	R	ACTGAATATTCCCTTTTCTCATCCT	117
Cdkn1a	F	GAACATCTCAGGGCCGAAAAC	R	CACTTCAGGGTTTTCTCTTGCA	96
Hey2	F	CTGCCAAGTTAGAAAAGGCTGA	R	CTCATGAAGTCTGTGGCAAGAG	118
Igf1r	F	GTTATCCACGACGATGAGTGC	R	AGTCACCGAATCGATGGTTTTC	150
Igf2r	F	GGAAGACACCAGAACCAGACA	R	TGACACTCATCCTCTGGAAGC	103
Insr	F	GAGAGGATGTGAGACGACGG	R	AGCAGTTCTCCAGCTCATGTAG	149
Gapdh	F	ACAACTCACTCAAGATTGTCAGCA	R	ATGGCATGGACTGTGGTCAT	121
Sdha	F	TTCCGTGTGGGGAGTGTATTG	R	ATTCTGCAGCTCCAGGGTCTC	135
Pmm1	F	ATCCGGGAGAAGTTTGTGGAA	R	GCTGTCTTCATCCAGGCTGTC	144
Ppia	F	AAGGGTTCCTCCTTTCACAGAA	R	GATGCCAGGACCTGTATGCTT	146

Table S7. Conditions used for immunostainings. Related to STAR Methods.

Staining	Antigen retrieval	Blocking	Primary antibody	Secondary antibody
IGF2	Digestion with 1% pronase (Protease from Streptomyces griseus, Sigma – P6911) in 1xPBS for 10 min at 37°C	15% Donkey serum (Sigma – D9663) in PBS	Goat anti-human IGF2 (1:50, R&D systems AF-292) overnight at 4°C	AF488 Donkey anti-goat (1:200, Jackson ImmunoResearch – 705-546-147), one hour at room temperature (RT)
IGF2R	Boiling for 20 min in Tris-EDTA buffer (10 mM Tris, 1 mM EDTA, 0.05% Tween 20, pH 9.0,)	Animal-free blocking solution (Vector – SP-5030)	Rabbit anti-IGF2R (1:400, Cell Signaling 14364) overnight at 4°C	AF594 Donkey anti-rabbit (1:200, Jackson ImmunoResearch 711-546-152), one hour at RT
YFP	Autoclaving for 15 min at 121°C in citric acid buffer (10 mM citric acid, pH 6.0, 0.05% Tween 20)	5% Donkey serum (Sigma – D9663) in PBS	Goat anti-GFP (1:200, Abcam – ab6673) overnight at 4°C	AF488 Donkey anti-goat (1:200, Jackson ImmunoResearch – 705-546-147), one hour at RT
CD31 (immune-histochemistry)	Boiling for 30 min in citric acid buffer (10 mM citric acid, pH 6.0, 0.05% Tween 20)	- 3% H2O2 solution (peroxidase inactivation) 30 min at RT;<br>- 10% Goat serum (Sigma – G9023) and 1% BSA in PBS	Rabbit anti-CD31 (1:50, Abcam – ab28364) overnight at 4°C	Goat anti-Rabbit IgG, biotinylated (1:1000, Abcam – ab6720), one hour at room temperature, then Streptavidin-horse radish peroxidase (1:250 Rockland S000-03), one hour at room temperature, then DAB (Dako – K3468), 3-20 minutes at RT
CD31 (immune-fluorescence – assay 1)	Boiling for 30 min in citric acid buffer (10 mM citric acid, pH 6.0, 0.05% Tween 20)	15% Donkey serum (Sigma – D9663) in PBS	Rabbit anti-CD31 (1:50, Abcam – ab28364) overnight at 4°C	AF594 Donkey anti-rabbit (1:200, Jackson ImmunoResearch 711-546-152), one hour at RT
CD31 (immune-fluorescence – assay 2)	Boiling for 20 min in Tris-EDTA buffer (10 mM Tris, 1 mM EDTA, 0.05% Tween 20, pH 9.0,)	Animal-free blocking solution (Vector – SP-5030)	Goat anti-CD31 (1:20, R&D – AF3628) overnight at 4°C	NL557-conjugated Donkey Anti-Goat (1:200, R&D – NL001), one hour at RT
F4/80	Heat-induced antigen retrieval in Target Retrieval Solution (pH=6) – Dako S236984-2	- Bloxall (peroxidase) Blocking Solution – Vector Labs SP-6000;<br>- Animal-Free Blocker - Vector Labs SP-5030	Rat anti-Mouse F4/80 (1:20, [CI:A3-1] – Bio-Rad MCA497) 1 hour at RT	- Rabbit anti-Rat IgG (H+L) (1:250, Bethyl A110-322A) 1 hour at room temperature;<br>- Anti-Rabbit HRP (ImmPress – Vector Labs MP-7451) 30 min at RT;<br>- DAB (ImmPact DAB Kit - Vector Labs SK-4105)
MCT1	Proteinase K digestion (Dako – S3020) for 3 minutes at room temperature	15% Donkey serum (Sigma – D9663) in PBS	Chicken anti-MCT1 (1:200, Merk Millipore – AB1286-I) overnight at 4°C	AF488 Donkey anti-chicken (1:200, Jackson ImmunoResearch – 703-546-155), one hour at RT

MCT4	Proteinase K digestion (Dako – S3020) for 3 minutes at room temperature	15% Donkey serum (Sigma – D9663) in PBS	Rabbit anti-MCT4 (1:500, Merck Millipore – AB3314P) overnight at 4°C	AF488 Donkey anti-rabbit (1:200, Jackson ImmunoResearch – 711-546-152), one hour at RT
Laminin	Proteinase K digestion (Dako – S3020) for 3 minutes at room temperature	15% Donkey serum (Sigma – D9663) in PBS	Rabbit anti-laminin (1:500, Dako – Z0097) overnight at 4°C	AF488 Donkey anti-rabbit (1:200, Jackson ImmunoResearch – 711-546-152), one hour at RT
Lectin	Digestion with 0.04% pepsin (Sigma – 10108057001) in 0.01M HCl, for 10min at 37°C	- 3% methanol (peroxidase inactivation) for 10min at RT;<br>- 2% bovine serum albumin, 1% skimmed dry milk and 0.1% Tween20, for 15min at RT	biotinylated lectin (1:250 isolectin B4, B-1205, Vector Laboratories) for 90min at 37°C	horseradish peroxide-conjugated streptavidin (1:500 Rockland Immunochemicals S000-03 for 60min at RT) followed by DAB (Sigma D3939) for 10min at RT
Cytokeratin	Digestion with 0.04% pepsin (Sigma – 10108057001) in 0.01M HCl, for 10min at 37°C	- 3% methanol (peroxidase inactivation) for 10min at RT;<br>- 2% bovine serum albumin, 1% skimmed dry milk and 0.1% Tween20, for 15min at RT	Rabbit anti-pan cytokeratin (1:75 Novus Biologicals nb600-579) overnight at 4°C	alkaline phosphatase-conjugated goat anti-rabbit (1:500 Abcam ab6722) for 60min at RT followed by NBT/BCIP containing levimasole to block endogenous phosphatase (Thermo Fisher Scientific 34070) for 10min at RT
EPCAM (frozen sections)	None	15% Donkey serum (Sigma – D9663) in PBS	Rat anti-mouse CD326/Epcam Clone G8.8 (1:50, BD Biosciences 552370) overnight at 4°C	AF594 Donkey anti-rat (1:250, Thermo Fisher Scientific A-21209) one hour at room temperature
