# Supplementary material for: The imprinted Igf2-Igf2r axis is critical for matching placental microvasculature expansion to fetal growth
Source: Dev Cell. 2022 Jan 10;57(1):63–79.e8. doi: 10.1016/j.devcel.2021.12.005 (PMC8751640; doi:10.1016/j.devcel.2021.12.005)
Supplement: Document S2. Article plus supplemental information [file mmc6.pdf]

Developmental Cell

Article

The imprinted *Igf2-Igf2r* axis is critical for matching placental microvasculature expansion to fetal growth

Graphical abstract

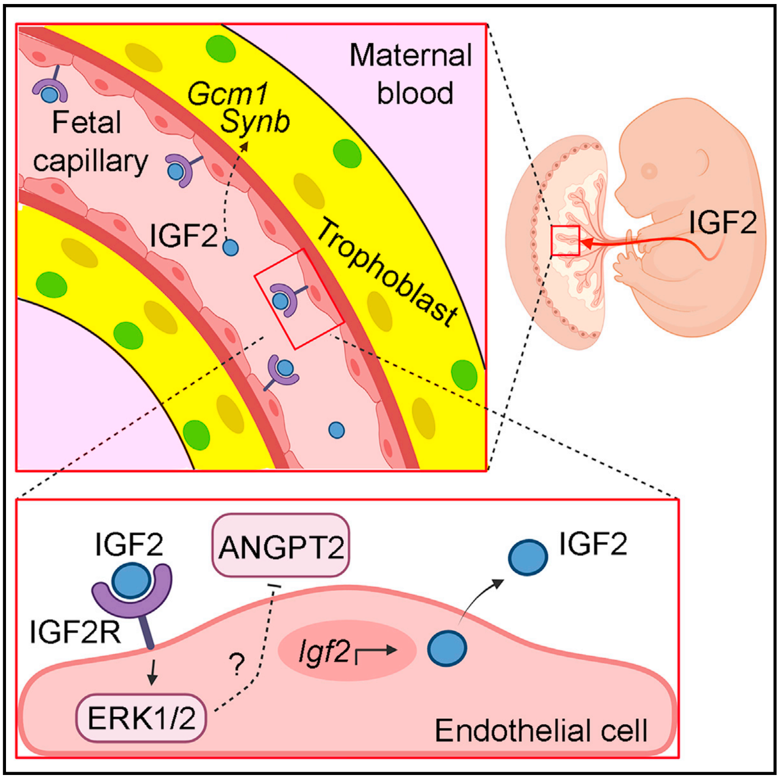

Highlights

- Fetus-derived IGF2 controls placental microvasculature expansion in late gestation
- The angiocrine effects of IGF2 are mediated via angiopoietins/Tek and IGF2R-ERK1/2
- Fetus-derived IGF2 also regulates trophoblast morphogenesis via *Gcm1* and *Synb*
- The imprinted *Igf2-Igf2r* axis matches placental development to fetal demand

Authors

Ionel Sandovici,
Aikaterini Georgopoulou,
Vicente Pérez-García, ...,
Cristina M. Branco,
Amanda N. Sferruzzi-Perri,
Miguel Constância

Correspondence

is299@cam.ac.uk (I.S.),
jmasmc2@cam.ac.uk (M.C.)

In brief

The fetal signals that coordinate placental development to the nutritional needs of the growing fetus are currently unknown. Using genetic manipulations in the mouse, Sandovici et al. show that IGF2 produced by the fetus is a key signal that controls the expansion of the placental vascular tree in late pregnancy.

Sandovici et al., 2022, Developmental Cell *57*, 63–79
January 10, 2022 © 2021 The Authors. Published by Elsevier Inc.
https://doi.org/10.1016/j.devcel.2021.12.005

CellPress

Developmental Cell

CellPress
OPEN ACCESS

Article

The imprinted *Igf2-Igf2r* axis is critical for matching placental microvasculature expansion to fetal growth

Ionel Sandovici,[1,2,3,*] Aikaterini Georgopoulou,[1,3,8] Vicente Pérez-García,[3,4,5,8] Antonia Hufnagel,[1] Jorge López-Tello,[3] Brian Y.H. Lam,[2] Samira N. Schiefer,[1] Chelsea Gaudreau,[2] Fátima Santos,[3,4] Katharina Hoelle,[1] Giles S.H. Yeo,[2] Keith Burling,[2] Moritz Reiterer,[6,7] Abigail L. Fowden,[3] Graham J. Burton,[3] Cristina M. Branco,[6,7,9] Amanda N. Sferruzzi-Perri,[3,9] and Miguel Constância[1,2,3,10,*]
[1]Department of Obstetrics and Gynaecology and National Institute for Health Research Cambridge Biomedical Research Centre, University of Cambridge, Cambridge CB2 0SW, UK
[2]Wellcome-MRC Institute of Metabolic Science and Medical Research Council Metabolic Diseases Unit, University of Cambridge, Cambridge CB2 0QQ, UK
[3]Centre for Trophoblast Research, Department of Physiology, Development and Neuroscience, University of Cambridge, Cambridge CB2 3EG, UK
[4]Epigenetics Programme, Babraham Institute, Cambridge CB22 3AT, UK
[5]Centro de Investigación Príncipe Felipe, Eduardo Primo Yúfera, 46012 Valencia, Spain
[6]Physiological Laboratory, Department of Physiology, Development and Neuroscience, University of Cambridge, Cambridge CB2 3EG, UK
[7]Center for Cancer Research and Cell Biology, Queen's University Belfast, Belfast BT9 7AE, UK
[8]These authors contributed equally
[9]These authors contributed equally
[10]Lead contact
*Correspondence: is299@cam.ac.uk (I.S.), jmasmc2@cam.ac.uk (M.C.)
https://doi.org/10.1016/j.devcel.2021.12.005

SUMMARY

In all eutherian mammals, growth of the fetus is dependent upon a functional placenta, but whether and how the latter adapts to putative fetal signals is currently unknown. Here, we demonstrate, through fetal, endothelial, hematopoietic, and trophoblast-specific genetic manipulations in the mouse, that endothelial and fetus-derived IGF2 is required for the continuous expansion of the feto-placental microvasculature in late pregnancy. The angiocrine effects of IGF2 on placental microvasculature expansion are mediated, in part, through IGF2R and angiopoietin-Tie2/TEK signaling. Additionally, IGF2 exerts IGF2R-ERK1/2-dependent pro-proliferative and angiogenic effects on primary feto-placental endothelial cells *ex vivo*. Endothelial and fetus-derived IGF2 also plays an important role in trophoblast morphogenesis, acting through *Gcm1* and *Synb*. Thus, our study reveals a direct role for the imprinted *Igf2-Igf2r* axis on matching placental development to fetal growth and establishes the principle that hormone-like signals from the fetus play important roles in controlling placental microvasculature and trophoblast morphogenesis.

INTRODUCTION

The mammalian fetus is dependent upon the placenta for nutrients and oxygen. Little is known, however, about how placental functional capacity adapts to meet fetal demands for growth. As gestation progresses, the increase in fetal size requires higher levels of nutrients via the placenta. Depending on the species, placental surface area for nutrient exchange increases 5- to 15-fold between mid and late gestation (Fowden et al., 2006). This remarkable adaptation is likely to occur, at least in part, in response to fetus-derived signals, but this important principle remains untested.

Imprinted genes play central roles in the fetal demand for, and the placental supply of, maternal nutrients (Constância et al., 2002, 2005; Coan et al., 2008; Angiolini et al., 2011). The insulin-like growth factor 2 (*Igf2*) gene encodes a polypeptide that is highly abundant in both fetal tissues and the fetal circulation. It is one of the most potent embryonic growth factors, affecting the metabolism, proliferation, survival, and differentiation of a wide variety of cell types (DeChiara et al., 1991; Baker et al., 1993; Gardner et al., 1999; Burns and Hassan, 2001). In the mouse, homozygous mutants are indistinguishable from growth-deficient littermates with deletion of paternal *Igf2* allele, while mutants with a disrupted maternal *Igf2* allele are phenotypically normal (DeChiara et al., 1991). In humans, reduced *IGF2* expression contributes to the fetal growth restriction in patients with Silver-Russell syndrome (SRS) (Azzi et al., 2014). Conversely, bi-allelic *IGF2* expression caused by loss of

This is an open access article under the CC BY license (http://creativecommons.org/licenses/by/4.0/).

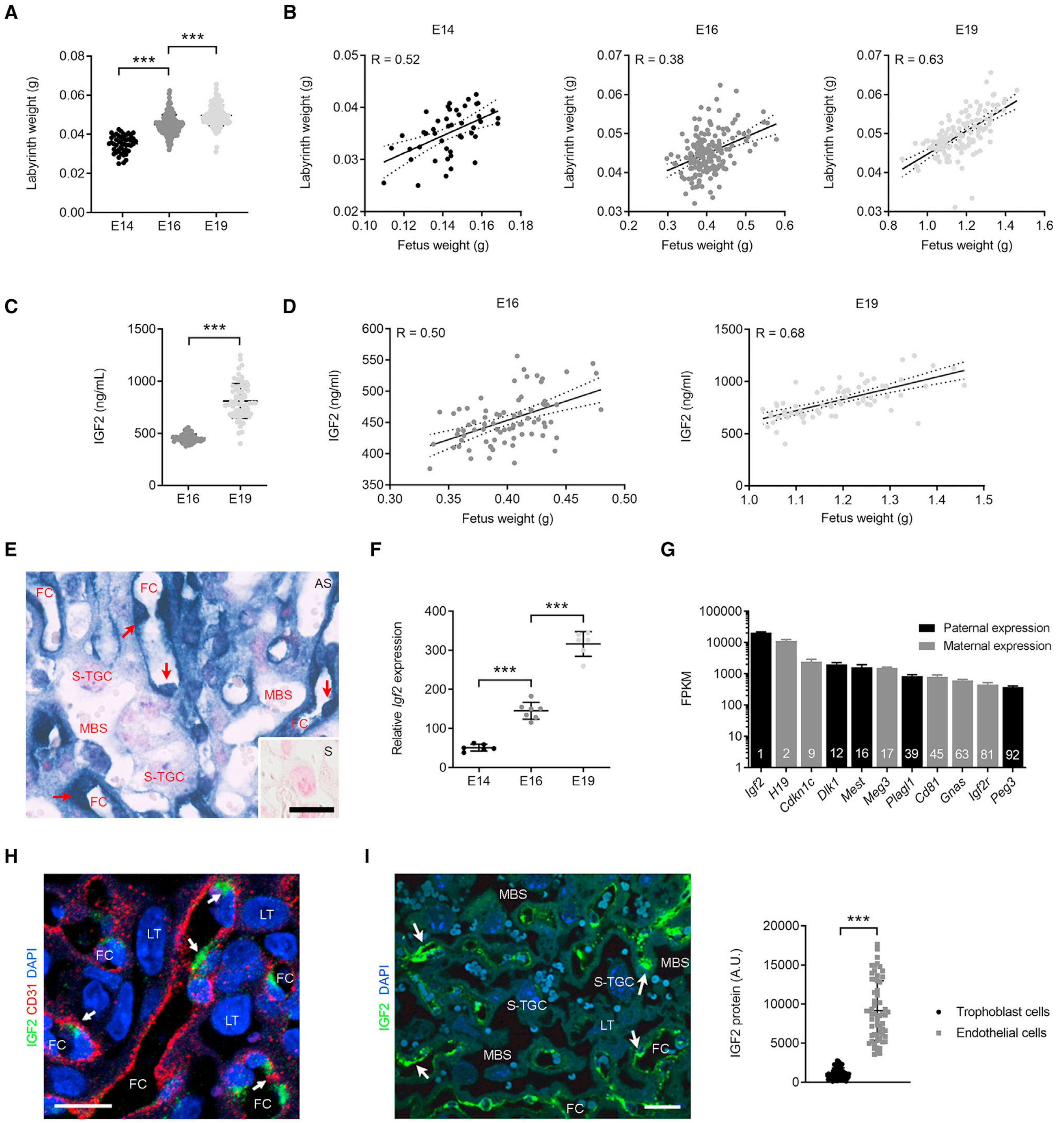

Figure 1. Lz expansion is associated with increasing levels of circulating and endothelial IGF2
(A) Weights of micro-dissected Lz.
(B) Linear correlation analyses between fetal and Lz weights: p = 0.002 (E14), p < 0.0001 (E16), and p < 0.0001 (E19) (n = 46–189 placentae from n > 10 L per group in [A] and [B]).
(C) Levels of IGF2 (ng/mL) in plasma of wild-type fetuses.
(D) Linear correlation analyses between fetal weights and circulating IGF2: p < 0.0001 (E16 and E19) (n = 70–79 per group in [C] and [D]).
(E) *Igf2* mRNA *in situ* hybridization (blue) in E14 wild-type Lz (red arrows—FPEC, feto-placental endothelial cells; AS, antisense probe; inset with S, sense probe; scale bars, 20 μm).
(F) Relative *Igf2* mRNA expression levels measured by qRT-PCR in FPEC from wild-type Lz (n = 6–7 per group).
(G) Imprinted genes that rank within top 100 expressed genes in E16 wild-type FPEC (FPKM, fragments per kilobase million; n = 4).

(legend continued on next page)

CellPress
OPEN ACCESS

imprinting is observed in Beckwith-Wiedemann patients (BWS), a syndrome characterized by somatic overgrowth and increased predisposition to tumors (Azzi et al., 2014).

IGF2 exerts its effects by binding to several IGF/INS (insulin) receptors (IGF1R, INSR, IGF1/INSR hybrids, and IGF2R) (Sferruzzi-Perri et al., 2017). IGF2 binds to IGF2R with the highest affinity, which leads to either IGF2 degradation in the lysosomes or signaling via G proteins (Okamoto et al., 1990; Maeng et al., 2009; Harris and Westwood, 2012). Additionally, IGF2R has further functions as a mannose 6-phosphate receptor (M6PR) and is also involved in the activation of latent transforming growth factor (TGF)-β1 (Ghosh et al., 2003). In the mouse, *Igf2r* is imprinted, being expressed only from the maternal chromosome (Barlow et al., 1991). Inactivation of the maternal *Igf2r* allele leads to body overgrowth and perinatal lethality (Lau et al., 1994). This phenotype is largely caused by an excess of extracellular IGF2, as shown by the rescue of overgrowth with the introduction of an *Igf2* null allele (Wang et al., 1994). More recently, an IGF2-binding mutant allele (*Igf2r*^{I1565A}) was also shown to result in overgrowth and lethality (Hughes et al., 2019). Imprinting of *IGF2R* in the human is a polymorphic trait, with a minority of cases showing evidence for maternal expression in fetal and/or placental tissues (Xu et al., 1993; Oudejans et al., 2001; Monk et al., 2006).

Here, we apply genetic approaches to define the signaling mechanisms that allow communication between the fetus and placenta, by creating mouse models with a growth mismatch between the two. Our work demonstrates that fetus and endothelial-derived IGF2 is essential for the appropriate expansion of the feto-placental microvasculature and the underlying trophoblast in late gestation. The interaction of circulating IGF2 and endothelial IGF2 with the trophoblast is essential for matching the placental surface area for nutrient exchange to the growth rate of fetal tissues.

RESULTS

Expansion of placental labyrinthine zone coincides with elevated levels of circulating and endothelial IGF2

The gas and nutrient exchange layer of the mouse placenta (labyrinthine zone [Lz]) increased in size with advancing gestational age (Figure 1A), matching the gain in fetal weight (Figure 1B). At E14 and E16, the positive correlation between Lz and fetal weights is weak-moderate, with a strong positive correlation at E19 (Figure 1B). This is a specific effect of the Lz layer, as placental weight in mice decreases at the very end of gestation (Coan et al., 2004). Concomitantly, fetal plasma IGF2 increased around 2-fold between E16 and E19 (Figure 1C). At these two developmental stages, we observed a moderate-strong positive correlation between fetal plasma IGF2 and fetal weights (Figure 1D). Within the Lz, *Igf2* expression was highest in feto-placental endothelial cells (FPEC) (Figure 1E), and its mRNA levels increased about 6-fold between E14 and E19 (Figure 1F). *Igf2* ranked as the highest expressed gene in FPEC RNA-seq transcriptome at E16, and several other known imprinted genes (Wei et al., 2014) ranked in the top 100 out of ~14,000 genes detected (Figure 1G; Table S1). IGF2 protein was highly expressed in FPEC (Figure 1H) and significantly higher than in the surrounding trophoblast cells (Figure 1I).

Fetal and endothelial IGF2 control Lz expansion

To explore whether fetus-derived IGF2 plays a direct role in placental development, we first used a conditional allele (*Igf2*$^{+/fl}$) to delete *Igf2* in the epiblast lineage using the *Meox2*^{Cre} line (Tallquist and Soriano, 2000; Figures 2A and S1). The deletion of the paternally inherited *Igf2* allele from embryonic organs and FPEC, but not extra-embryonic tissues, led to placental growth restriction from E14 onward (Figure 2B). Stereological analyzes indicated that only the placental compartments containing embryonic-derived structures (i.e., Lz and the chorionic plate [Cp]) were smaller in the *Meox2*$^{Cre/+}$; *Igf2*$^{+/fl}$ mutants (referred subsequently as *Igf2*^{EpiKO}) (Figure 2C). The continuous expansion of the Lz, measured as volume increase between E14 and E19 was severely compromised in mutants (Figure 2C). The overall volume, surface area, and total length of fetal capillaries (FCs) were normal at E14 but became abnormal from E16 onward (Figures 2D and S2A). All other components of the Lz, not originating from the embryonic lineage, i.e., labyrinthine trophoblast (LT) and maternal blood spaces (MBSs), were also reduced in volume to a similar extent as the FC (Figure 2D). These findings provide evidence for a role of fetus-derived IGF2 on the expansion of Lz in late gestation.

IGF2 is highly expressed in FPEC, as previously shown (Figures 1E–1I). Therefore, we next tested whether endothelial-derived IGF2 plays a role in placental development. Paternal *Igf2* allele deletion in the fetal endothelium, including FPEC, using the *Tek*^{Cre} line (Kisanuki et al., 2001; Figures 2E and S3A–S3E) led to a moderate, but significant fetal and placental growth restriction, evident from E16 onward (Figure 2F). Mutant *Tek*$^{Cre/+}$; *Igf2*$^{+/fl}$ (referred subsequently as *Igf2*^{ECKO}) placentae had reduced volumes of Cp and Lz at both E16 and E19 (Figure 2G) but less striking when compared with *Igf2*^{EpiKO} mutants (Figure 2C). Within the Lz, the LT was reduced at both E16 and E19, while the MBSs and FCs were comparable to controls at E16 but significantly reduced at E19 (Figures 2H and S2B).

We next tested if IGF2 derived from hematopoietic cells (HCs) contributed to the *Igf2*^{ECKO} phenotype and deleted the paternal *Igf2* allele in the hematopoietic lineage using the *Vav*^{iCre} line (de Boer et al., 2003). Efficient deletion of *Igf2* in HC (referred to subsequently as *Igf2*^{HCKO}, Figures S3F and S3G) did not have any significant impact on fetal and placental growth or on Lz expansion between E14 and E19 (Figure S3H). Additionally, *Igf2*^{HCKO}

(H) Double immunostaining for IGF2 and CD31 in E19 wild-type placenta. Endothelial cells are very thin and hard to detect except where the cytoplasm is more voluminous around the nucleus, with intense IGF2 stain (white arrows). Transmembrane glycoprotein CD31 immunostaining is in the membrane and largely marks endothelial intercellular junctions (scale bars, 20 μm).

(I) Semi-quantitative measurement of IGF2 protein in FPEC versus trophoblast cells (E19 wild-type Lz, n = 60 cells per group from two placentae). White arrows—endothelial cells; scale bars, 50 μm. For (E), (H), and (I): FC, fetal capillaries; MBS, maternal blood spaces; LT, labyrinthine trophoblast cells; S-TGC, sinusoidal trophoblast giant cells. Data in (A), (C), (F), (G), and (I) are presented as averages ± standard deviation (SD); ***p < 0.001 calculated by one-way ANOVA plus Tukey's multiple comparisons test in (A) and (F) or by unpaired t test with Welch's correction in (C) and (I). See also Table S1.

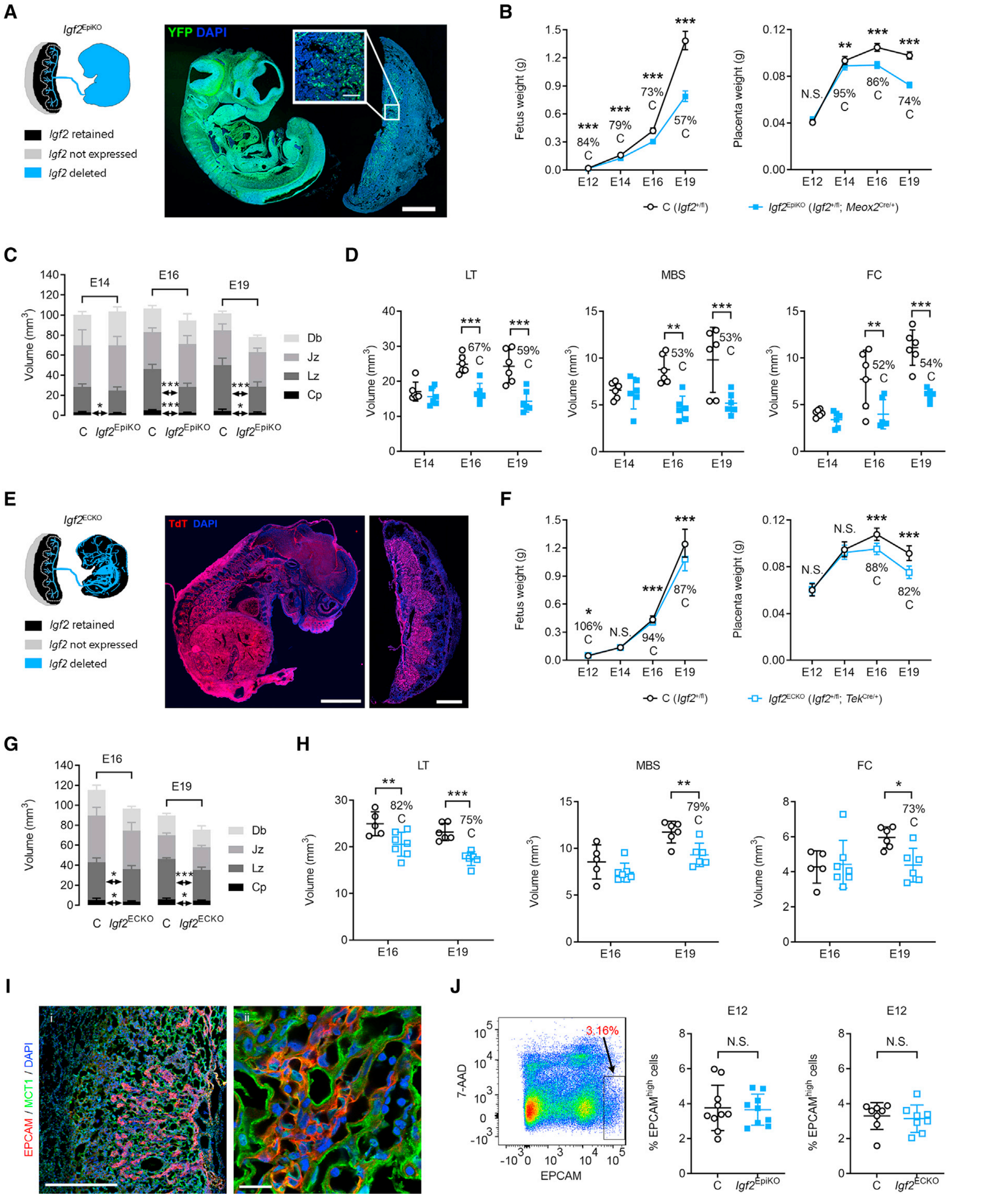

Figure 2. Deletion of *Igf2* in the epiblast or endothelium impairs Lz expansion

(A) Left: schematic of *Igf2* expression in conceptuses with conditional deletion driven by *Meox2*^{Cre}. Right: immunostaining for YFP (green) in a representative fetus and placenta paraffin section at E12 of gestation, double transgenic for *Meox2*^{Cre} and *Rosa26*^{fl}STOP^{fl}YFP^{10} reporter. YFP expression in the placenta is localized to the Lz and Cp (high magnification, inset). Blue—DAPI stain for nuclei; scale bars: 1 mm (low magnification) and 100 μm (high magnification).

(B) Fetal and placental growth kinetics, measured as average wet-weights for each genotype per litter (E12: n = 10 L [n = 41 controls {C} and n = 32 *Igf2*^{EpiKO}]; E14: n = 25 L [n = 114 C and n = 88 *Igf2*^{EpiKO}]; E16: n = 37 L [n = 154 C and n = 127 *Igf2*^{EpiKO}]; E19: n = 37 L [n = 164 C and n = 121 *Igf2*^{EpiKO}]).

(C) Absolute volumes of the placental layers (Db, decidua basalis; Jz, junctional zone; Lz, labyrinthine zone; Cp, chorionic plate), measured by stereology (n = 6 per group).

(legend continued on next page)

mutant placentae had FC densities similar to that of controls at E19 (Figure S3I).

Although the "small" Lz phenotype is observed in *Igf2*^{EpiKO} and *Igf2*^{ECKO} mutants only in later gestation, it could originate as result of a reduced pool of multipotent labyrinth trophoblast progenitor (LaTP) cells. LaTP cells are detected as clusters of EPCAM^{high}-positive cells between E9.5 and E12.5 (Ueno et al., 2013; Figure 2I). Using flow cytometry analysis at E12, we found no significant difference in the percentage of EPCAM^{high}-positive cells between *Igf2*^{EpiKO} and *Igf2*^{ECKO} mutants and their corresponding littermate controls (Figure 2J).

We conclude that the Lz phenotype observed in *Igf2*^{EpiKO} and *Igf2*^{ECKO} mutants is not the consequence of a reduced pool of multipotent LaTP cells due to defective IGF2 signaling in early placental development. However, our data cannot exclude defects in the differentiation potential of the multipotent LaTP cells. The more severe impact on Lz growth observed in *Igf2*^{EpiKO} mutants compared with *Igf2*^{ECKO} mutants, and the normal Lz growth in *Igf2*^{HCKO} mutants suggests that full Lz expansion in late gestation requires both fetus-derived and endothelial-derived IGF2, but not hematopoietic-cell derived IGF2.

Fetus-derived IGF2 is essential for placental morphogenesis and microvasculature expansion

To uncover molecular signatures associated with the defective Lz expansion in *Igf2*^{EpiKO} mutants, we performed an expression microarray analysis in micro-dissected Lz samples at E19, when the Lz expansion and fetal demand for nutrients reach their maximum in absolute terms. Differentially expressed genes (DEGs) were enriched in genes implicated in vasculature development and immune responses (Figures 3A, S4A, and S4B). We identified a classic molecular signature of impaired angiogenesis—reduced angiopoietin-Tie2/TEK (tyrosine kinase, endothelial) signaling (Augustin et al., 2009; Figure 3B; Table S2). Lower levels of *Angpt1* and *Tek*, and increased expression of *Angpt2* were validated by qRT-PCR in an independent set of biological samples at E19 and E16, but not at E14 (Figure 3B). Consistent with the roles of the angiopoietin-Tie2/TEK signaling in endothelial cell survival and proliferation (Augustin et al., 2009), TUNEL staining revealed a 6-fold increase in apoptotic cell frequency in mutants at E16, specifically in the Lz (Figure 3C). We then co-stained E16 *Igf2*^{EpiKO} mutant placentae for TUNEL and laminin, a marker for the fetal capillary basement membrane (Milner et al., 1998). Our analysis revealed that the majority of TUNEL^{+} cells (86.8% ± 4.25%) co-express laminin (Figure 3D), indicating that a large proportion of the apoptotic cells are FPEC. Furthermore, endothelial cell proliferation measured by flow cytometry was significantly reduced at E16 (Figures 3E and S4C), a finding confirmed by immunofluorescence (Figure S4D).

In addition to vascular pathways (which, besides *Angpt1*, *Angpt2*, and *Tek*, also include DEGs such as *Dll4*, *Egfl6*, *Fzd4*, *Pdgfc*, and *Slit2*, see Table S2), the expression microarrays identified transcriptional upregulation of genes related to immune responses and leukocyte migration (Figure 3A). Among these was *Adgre1*, a gene that encodes the glycoprotein F4/80, a highly specific cell-surface marker for murine macrophages (Austyn and Gordon, 1981). The upregulation of *Adgre1* was confirmed by qRT-PCR in Lz also at E16 (Figure 3F). Immunostaining for F4/80 showed that the total number of macrophages in Lz was significantly higher in mutants than controls (Figure 3G). Additionally, clusters of macrophages surrounding feto-placental capillaries were found exclusively in mutants (Figure 3G). Next, we assessed the impact of the increased cell death, reduced cell proliferation, and macrophage infiltration on capillary remodeling across gestation by CD31 immunostaining (marking endothelial cells). The density of FC was dramatically reduced at E16 and E19 (Figure 3H). CD31-stained or methylene blue-stained resin sections also revealed small areas of feto-placental capillaries with accumulations of leucocytes but lacking endothelial cells or obstructed and thrombotic capillaries surrounded by disorganized and fragmented endothelial cells in late gestation (Figure S4E). Using electron microscopy, we did not observe evidence for feto-maternal barrier interruption that would allow for mixing between maternal and fetal blood, even in areas with disorganized FPECs (Figures S4F and S4G).

Importantly, the array data indicated downregulation of key genes involved in syncytiotrophoblast differentiation (i.e., *Gcm1* and *Synb*—which are expressed specifically in layer II of the syncytiotrophoblast [SynT-II], which is closest to FC; see Table S2). To validate these observations, we performed qRT-PCR and confirmed significant transcriptional reductions across late gestation of SynT-II-specific genes (Rawn and Cross, 2008;

(D) Absolute volumes of Lz components, measured by stereology (LT, labyrinthine trophoblast; MBSs, maternal blood spaces; FCs, fetal capillaries) (n = 6 per group).

(E) Left: schematic representation of *Igf2* expression in conceptuses with conditional deletion driven by *Tek*^{Cre}. Right: representative confocal microscopy of frozen sections from a fetus and its corresponding placenta, double transgenic for *Tek*^{Cre} and Ai9(RCL-tdT) reporter at E16 of gestation. Scale bars: 2 mm (fetus) and 1 mm (placenta).

(F) Fetal and placental growth kinetics (E12: n = 5 L [n = 17 C and n = 16 *Igf2*^{ECKO}]; E14: n = 8 L [n = 26 C and n = 34 *Igf2*^{ECKO}]; E16: n = 13 L [n = 60 C and n = 46 *Igf2*^{ECKO}]; E19: n = 7 L [n = 31 C and n = 27 *Igf2*^{ECKO}]).

(G) Absolute volumes of the placental layers measured by stereology (n = 5–7 per group).

(H) Absolute volumes of Lz components, measured by stereology (n = 5–7 per group).

(I) Double immunostaining for EPCAM (epithelial cell adhesion molecule) (red) and MCT1 (monocarboxylate transporter 1) (green) in a representative frozen placental section at E12 of gestation. EPCAM expression is observed as clusters of positive cells within the Lz placenta. Blue—DAPI (4′,6-diamidino-2-phenylindole) stain for nuclei; scale bars: 500 μm (left panel) and 20 μm (right panel).

(J) Analysis of EPCAM^{high}-positive cells by flow cytometry. Left panel: example of gating used to identify EPCAM^{high}-positive cells (the viability dye 7-amino-actinomycin D [7-AAD] was used to exclude dead cells). Right: quantification of placental EPCAM^{high}-positive cells at E12 in conceptuses with conditional *Igf2* deletion driven by *Meox2*^{Cre} (n = 10 C and n = 9 *Igf2*^{EpiKO} from 2 L) or *Tek*^{Cre} (n = 8 C and n = 8 *Igf2*^{ECKO} from 2 L). For all graphs data are shown as averages; error bars represent SD in (C), (D), (G), (H), and (J) or 95% confidence intervals (95% CI) in (B) and (F); N.S.—statistically not significant; *$p < 0.05$; **$p < 0.01$; ***$p < 0.001$ calculated by a mixed effects model in (B) and (F) (see STAR Methods), two-way ANOVA plus Sidak's multiple comparisons tests in (D) and (H) or unpaired t tests in (C), (G), and (J). See also Figures S1–S3.

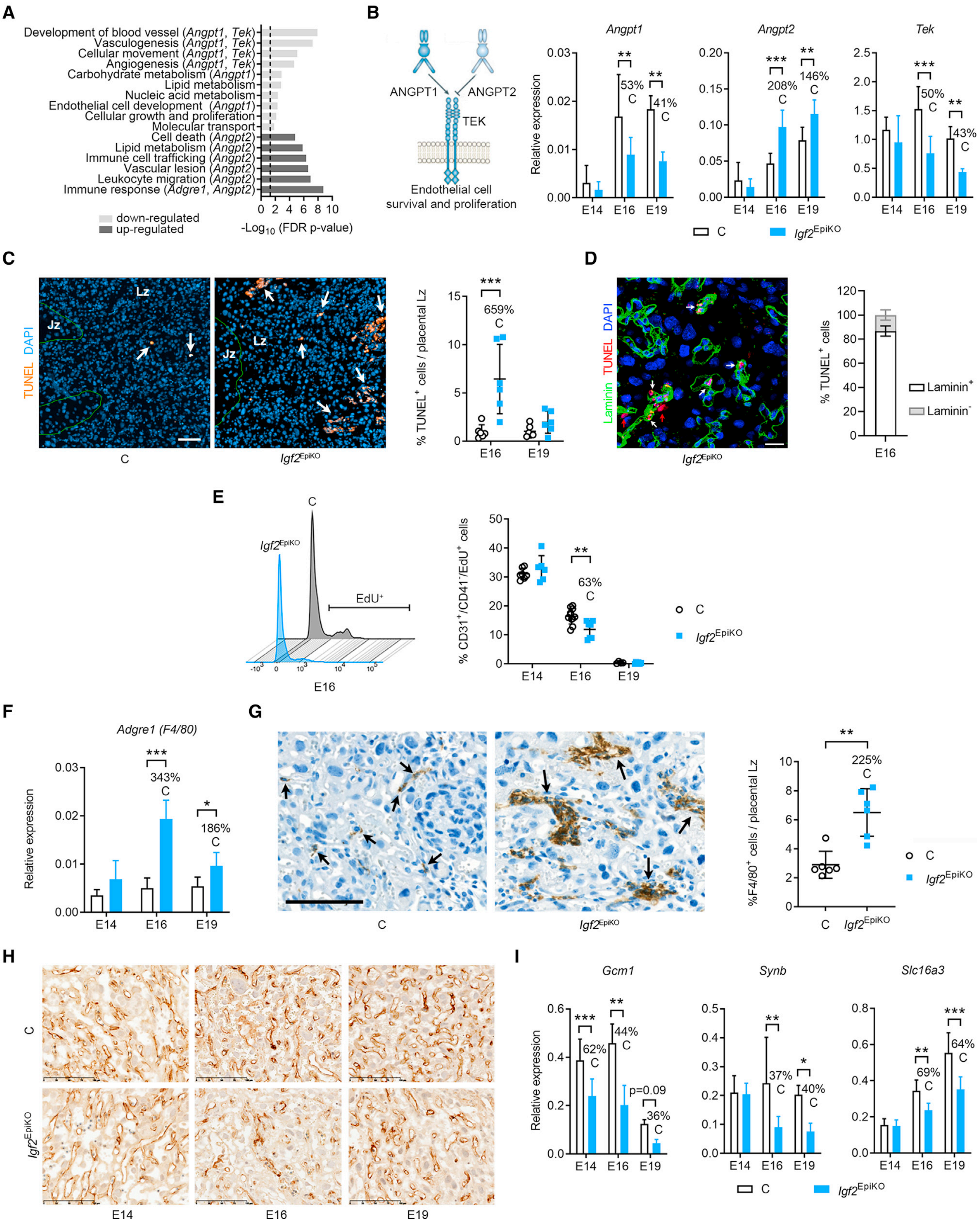

(legend on next page)

CellPress
OPEN ACCESS

Nagai et al., 2010) *Gcm1*, *Synb*, and *Slc16a3* (Figure 3I). However, only the SynT-I specific gene *Slc16a1* (Rawn and Cross, 2008; Nagai et al., 2010; Hughes et al., 2013) was modestly downregulated, but not *Ly6e* and *Syna* (Figure S4H).

Together, our data show that lack of fetus-derived IGF2 triggers dysregulation of angiopoietin-Tie2/TEK signaling in late gestation, with consequent reduced FPEC proliferation and excessive cell death with associated placental macrophage infiltration. It also highlights that fetus-derived IGF2 supports normal development of the trophoblast cells, particularly the SynT-II layer, in a paracrine/endocrine manner, with a knock-on effect on the development of MBS.

Endocrine IGF2 is a fetus-derived signal that matches placental nutrient supply capacity to fetal demands for growth

To provide further insights into the roles of fetus-derived IGF2 in matching placental and fetal growth, we analyzed five genetic models with either deletion of the paternal *Igf2* allele in fetal tissues, endothelium, trophoblast or ubiquitously, or overexpression of *Igf2* achieved through loss of imprinting in fetal tissues (Figure 4). For these models, we used flow cytometry to count FPEC, defined as $CD31^{+}/CD41^{-}$ cells (Rhodes et al., 2008; Figures S5A–S5C), and measured Lz weight and circulating IGF2 levels. In $Igf2^{EpiKO}$ mutants, as expected from the immunostainings shown in Figure 3H, we observed a severe deficit in the total number and the proportion of FPEC at E16 and E19, but normal values at E14 (Figure 4A). The linear Lz expansion expected with gestational age was not observed in this model, matching the severe reductions in FPEC numbers and circulating IGF2 (Figure 4A). In contrast, in $Igf2^{ECKO}$ mutants lacking endothelial *Igf2*, circulating levels of IGF2 were only moderately reduced and total numbers of FPEC, but not relative numbers, were only significantly reduced at E19 (Figure 4B). Lz expansion in this model was only blunted at the end of gestation (Figure 4B). A deletion of *Igf2* specifically in the trophoblast cells of the placenta using $Cyp19^{Cre}$ (Wenzel and Leone, 2007) ($Igf2^{+/fl}$; $Cyp^{Cre/+}$ referred subsequently as $Igf2^{TrKO}$) (Figures 4C and S6A–S6E) did not result in changes in FPEC numbers and circulating IGF2, demonstrating that FPEC expansion is independent of trophoblast-derived IGF2. The rate of Lz expansion was normal in this model (Figure 4C). Ubiquitous deletion of *Igf2* in embryo and trophoblast using CMV^{Cre} (Schwenk et al., 1995) ($Igf2^{+/fl}$; $CMV^{Cre/+}$ referred subsequently as $Igf2^{UbKO}$) (Figures 4D and S6F) led to a loss of FPEC similar to that observed in the $Igf2^{EpiKO}$ mutants, further demonstrating that trophoblast-derived IGF2 does not contribute significantly to FPEC expansion. Lz weight was severely reduced from E14, in line with the near complete absence of IGF2 in fetal circulation (Figure 4D). Conversely, reactivating the transcriptionally silent maternal *Igf2* allele in $H19DMD^{fl/+}$; $Meox2^{+/Cre}$ mutants (Srivastava et al., 2000) (referred subsequently as *H19*-DMD^{EpiKO}) (Figures 4E, S6G, and S6H), which led to increased levels of circulating IGF2, was associated with an increase of Lz weight and higher numbers of FPEC at E16 and E19 (Figure 4E). Taken together, these results show that IGF2 produced by fetal organs and secreted into the fetal circulation stimulates the expansion of Lz, matching FPEC numbers to the fetal demand for growth.

IGF2 signaling controls expression of FPEC-derived angiogenic factors

To provide further molecular insights into the angiocrine roles of IGF2 on microvasculature expansion, we carried out RNA-seq analysis on FACS-isolated endothelial cells from E16 Lz of $Igf2^{EpiKO}$ and $Igf2^{ECKO}$ mutants and their corresponding controls (Figures 5, S5C, and S5D). Gene ontology (GO) analysis of DEGs in $Igf2^{EpiKO}$ mutants showed enrichment of biological processes related to immune responses, cell migration, impaired cell proliferation and angiogenesis, extracellular matrix organization, and response to hypoxia (Figures 5A and 5B; Table S3). We validated representative DEGs using qRT-PCR in independent biological samples, including genes encoding proteins secreted by the endothelial cells into the extracellular space that have known anti-angiogenic effects (e.g., *Angpt2* [Augustin et al., 2009], *Adamts1* [Lee et al., 2006], *Cxcl10* [Angiolillo et al., 1995], and *Thbs1* [Lawler and Lawler, 2012]), factors implicated in cell migration and response to hypoxia (*Edn1*, Lankhorst et al., 2016), an interferon-response gene (*Iigp1*, Uthaiah et al., 2003), an inhibitor of cell proliferation (*Cdkn1a*, Vidal and Koff, 2000), and a regulator of embryonic vascular development (*Hey2*, Fisher et al., 2004) (Figure 5C). With the notable exception of *Hey2*, these DEGs were also identified as DEGs in $Igf2^{ECKO}$ mutants, including the upregulation of *Angpt2*, suggesting that these transcriptional changes are the outcome of autocrine

Figure 3. Lack of fetus-derived IGF2 reduces the expansion of feto-placental microvasculature in late gestation

(A) Functions enriched in DEGs at E19.

(B) qRT-PCR analysis of angiopoietin-Tie2/TEK signaling components in Lz (n = 6–8 per group).

(C) TUNEL (terminal deoxynucleotidyl transferase dUTP nick end labeling) staining in E16 Lz (arrows point to apoptotic cells) and data quantification (n = 6 samples per group); scale bars, 50 μm.

(D) Left: representative double immunostaining for TUNEL (red) and laminin (green, marker of feto-placental capillaries) in the Lz of an E16 $Igf2^{EpiKO}$ mutant placenta (DAPI, blue marks the nuclei; white and red arrows indicate $TUNEL^{+}$ FPECs and LT, respectively; scale bars, 25 μm). Right: quantification of $TUNEL^{+}$ cells that are positive or negative for laminin (n = 6 $Igf2^{EpiKO}$ mutant placentae).

(E) Feto-placental endothelial cell (FPEC) proliferation measured by flow cytometry (left—representative histograms at E16; right—data quantification; n = 4–11 per group).

(F) qRT-PCR analysis of *Adgre1* in Lz.

(G) Representative F4/80 immunostainings in E16 Lz (arrows indicate macrophages). Scale bars, 100 μm. Right: percentage of macrophages/Lz at E16 (n = 6–8 samples per group).

(H) Representative CD31 immunostaining in Lz (scale bars, 100 μm).

(I) qRT-PCR analysis for SynT-II (syncytiotrophoblast layer II) marker genes. For all graphs, data are presented as averages or individual values; error bars are SD; *p < 0.05, **p < 0.01, ***p < 0.001 by two-way ANOVA plus Sidak's multiple comparisons tests in (B), (C), (E), (F), and (I) or Mann-Whitney tests in (G). See also Figure S4 and Table S2.

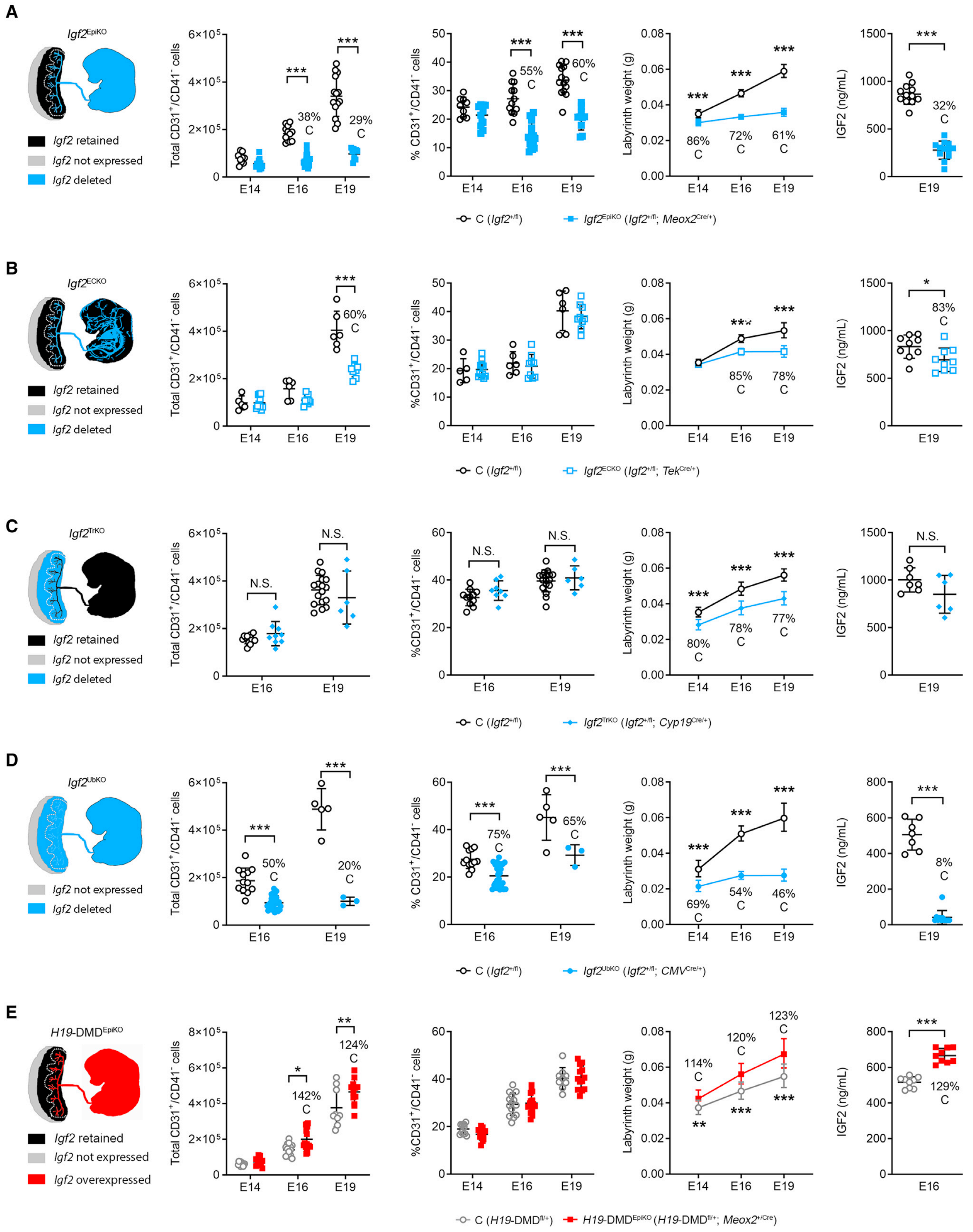

(legend on next page)

IGF2 actions on FPECs (Figure 5D; Table S3). Next, we searched for transcription factor (TF)-binding motifs enriched within the promoters of all DEGs found in the *Igf2*^{EpiKO} model. This analysis identified significant enrichments for binding sites of four TFs encoded by DEGs—KLF4, EGR1, IRF7, and HEY2 (Figure 5E; Table S3). Significantly, the four TFs control the expression of several proteins involved in angiogenesis (labeled with * in Figure 5F and further presented in Table S4), some of which are secreted by the endothelial cells into the extracellular space (Table S4). This analysis also highlighted several chemokines that were upregulated in FPEC (such as CCL2 [Gregory et al., 2006] and IL15 [Fehniger and Caligiuri, 2001]) that are likely involved in attracting and modulating the activity of macrophages that surround the feto-placental capillaries (as shown in Figure 3G). Thus, based on our data, we propose that IGF2 signaling is necessary for proliferation and survival of FPECs.

IGF2 signaling on FPEC is mediated by IGF2R *in vitro* and *in vivo*

To further investigate the role of IGF2 in fetal capillary remodeling and identify the receptors that mediate its effects on endothelial cells, we isolated FPEC from E16 wild-type Lz and cultured them *ex vivo* (Figures 6A–6C). Only *Igf1r* and *Igf2r* receptors were expressed in FPEC both *in vivo* (Figure 6D) and *ex vivo* (Figure 6E). When cultured *ex vivo* for 10 days (passage one) FPEC switch off *Igf2* transcription, which differs from FPEC freshly isolated by FACS (Figure 6F). Exposure of cultured FPEC to exogenous IGF2 significantly increased their ability to form capillary-like tube structures when seeded on matrigel (Figures 6G and 6H), demonstrating that IGF2 exerts direct angiogenic effects on FPEC. We also exposed cultured FPEC to IGF2^{Leu27}, an analog previously shown to bind to IGF2R with high selectivity (Beukers et al., 1991), which stimulated capillary-like tube formation although to a lesser extent compared with IGF2 (Figures 6G and 6H). When FPEC were treated with IGF2 and picropodophyllin (PPP), a small molecule that inhibits phosphorylation of IGF1R without interfering with INSR activity (Girnita et al., 2004), their ability to form capillary-like tube structures was very similar to that of cells treated with IGF2 alone (Figures 6G and 6H). Thus, IGF2 exerts direct angiogenic effects on primary FPEC, which are mediated by IGF2R and are independent of IGF1R.

To investigate further the observed effects of IGF2R in mediating IGF2 actions on primary FPECs, we performed *Igf2r* knockdown using siRNA and investigated the impact on cell proliferation and intracellular signaling with or without exogenous IGF2 stimulation (Figures 6I–6L). Upon IGF2 stimulation, efficient *Igf2r* knockdown led to reduced FPEC proliferation, demonstrating that the pro-proliferative actions of IGF2 on FPEC require IGF2R (Figures 6I and 6J). *Igf2r* knockdown also resulted in reduced FPEC proliferation, even in the absence of IGF2 stimulation, suggesting that IGF2R is required for normal FPEC proliferation, independent of IGF2 (Figure 6J). Additionally, FPEC stimulated with IGF2 for 96 h, but lacking IGF2R showed significant upregulation of *Angpt2* mRNA levels (Figure 6K). Acute IGF2 stimulation did not activate AKT, a key signaling node downstream of IGF1R (Figure 6L). AKT phosphorylation was not affected by the IGF2R knockdown (Figure 6K). However, we observed a significant delay in pERK1/2 phosphorylation upon acute stimulation of FPEC with IGF2 (Figure 6K). These data demonstrate both IGF2-independent and IGF2-dependent actions of IGF2R in controlling FPEC proliferation and highlight the role of ERK pathway in mediating the actions of IGF2 on FPEC via IGF2R.

We further confirmed these *in vitro* findings using conditional deletions of IGF1R and IGF2R receptors *in vivo*. Accordingly, efficient homozygous deletion of *Igf1r* from the endothelium (*Igf1r*^{ECKO}) did not have any significant impact on fetal, whole placenta or Lz growth kinetics, nor did it alter the total and relative numbers of FPEC/Lz, apart from a slight increase in the percentage of FPEC at E19 (Figure S7). Strikingly, the deletion of maternally expressed *Igf2r* allele from the endothelium (*Igf2r*^{ECKO}—see Figures 7A–7C) resulted in a reduction in the percentage of FPEC/Lz at both E16 and E19 (Figure 7D), further confirmed by a reduced density of CD31^{+} cells by immunofluorescence staining (Figure 7E). The total number of FPEC/Lz was also significantly reduced at E16 but became normal at E19 (Figure 7D). Notably, the overall placenta and Lz were overgrown, from E16 onward (Figures 7C and 7F), coincident with an increase in levels of circulating IGF2 in plasma (Figure 7G). Together, our *in vitro* and *in vivo* experiments demonstrate that IGF2R mediates, at least partially, the angiocrine actions of IGF2 on FPEC.

DISCUSSION

In this study, we identify the imprinted *Igf2-Igf2r* axis as a key pathway that controls the expansion of the placental vascular tree in late gestation and demonstrate that fetus-derived signals are important regulators of placental development and function. Although a vast number of genetic pathways have been discovered that are important for the development of different cell types in the placenta and the fetus, there are no functional genetic investigations to date on how the fetus signals its nutrient requirements to the placenta and how the placenta matches these demands. We tackled these questions with an experimental design based on the manipulation of the growth rate of fetal tissues independent of the placenta, and vice versa, in the mouse. We used conditional targeting of imprinted genes with

Figure 4. Genetic models of mismatched placental and fetal growth reveal circulating IGF2 as a major endocrine regulator of FPEC and Lz expansion

(A–E) Column 1: schematic diagrams of the genetic models: *Igf2*^{EpiKO} (A), *Igf2*^{ECKO} (B), *Igf2*^{TrKO} (C), *Igf2*^{UbKO} (D), and *H19*-DMD^{EpiKO} (E). Columns 2 and 3: total numbers (column 2) and proportion of FPEC/Lz (column 3), measured by flow cytometry (n conceptuses per group: *Igf2*^{EpiKO}: n = 9–18; *Igf2*^{ECKO}: n = 5–11; *Igf2*^{TrKO}: n = 6–17; *Igf2*^{UbKO}: n = 3–26; *H19*-DMD^{EpiKO}: n = 9–15). Column 4: Lz growth kinetics (*Igf2*^{EpiKO}: n = 9–20 L; *Igf2*^{ECKO}: n = 3–9 L; *Igf2*^{TrKO}: n = 4–9 L; *Igf2*^{UbKO}: n = 3–8 L; *H19*-DMD^{EpiKO}: n = 3–4 L). Column 5: IGF2 levels (ng/mL) in plasma (n per group: *Igf2*^{EpiKO}: n = 12; *Igf2*^{ECKO}: n = 9; *Igf2*^{TrKO}: n = 6–7; *Igf2*^{UbKO}: n = 7–11; *H19*-DMD^{EpiKO}: n = 9). Data are shown as averages or individual values and error bars are SD (columns 2, 3, and 5) and 95% CI (column 4). N.S.—not significant; *$p < 0.05$; **$p < 0.01$; ***$p < 0.001$ calculated by two-way ANOVA plus Sidak's multiple comparisons tests (second and third columns), mixed effects model (fourth column) or Mann-Whitney tests (fifth column). See also Figures S5A, S5B, and S6.

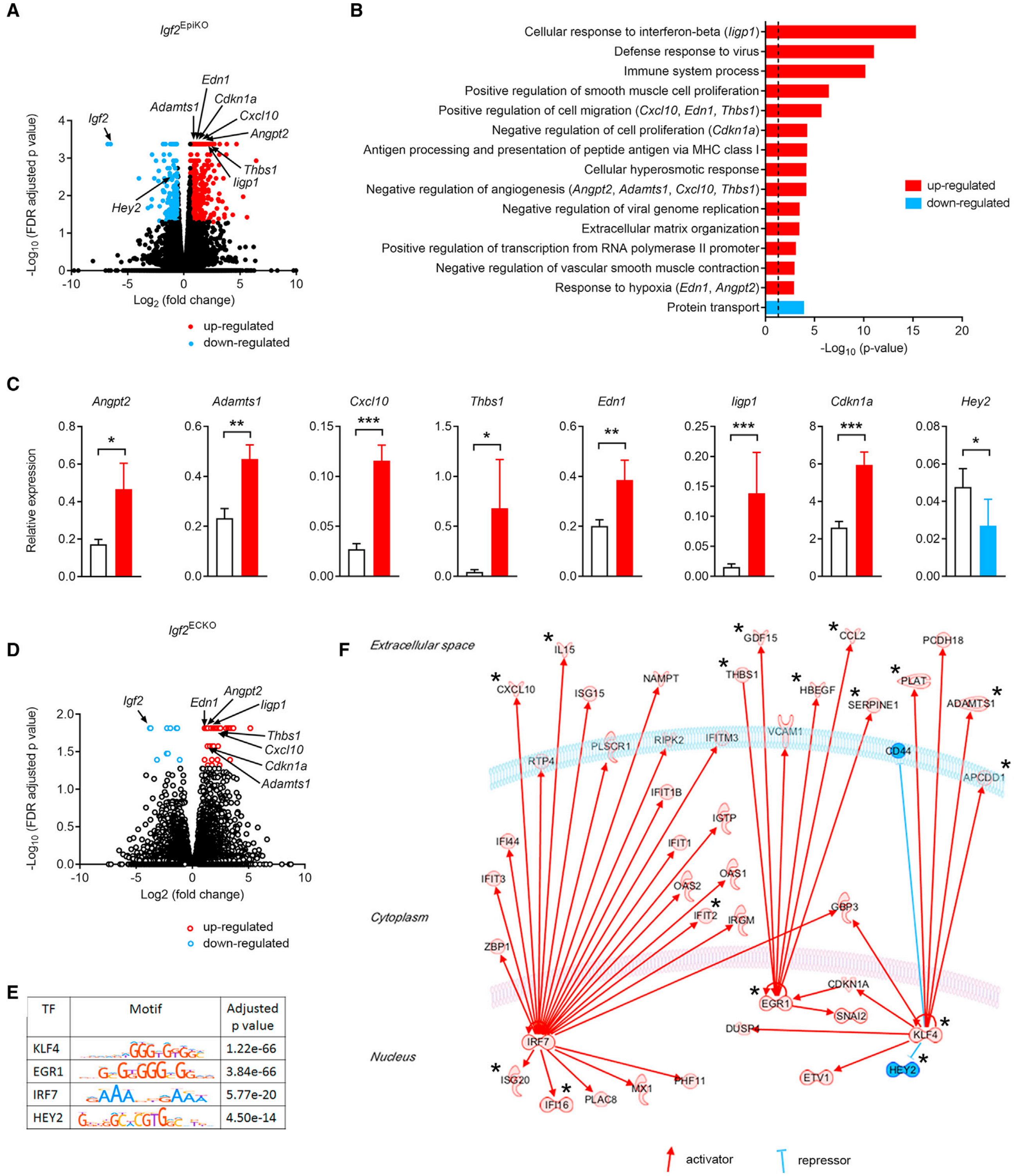

Figure 5. IGF2 signaling regulates angiogenic properties of endothelial cells

(A) Volcano plot representation of DEGs identified by RNA-seq in E16 FPEC (*Igf2*^{EpiKO} versus controls). Significant upregulated and downregulated DEGs (false discovery rate [FDR] < 0.05) are shown with red and blue, respectively.

(B) Top scoring biological processes enriched in DEGs. Biologically validated DEGS are listed in parentheses. The dotted line corresponds to FDR-corrected p value of 0.05.

(C) Biological validation. Data are shown as averages (n = 11–12 samples per group); error bars are SEM; * $p < 0.05$, ** $p < 0.01$, *** $p < 0.001$ calculated by Mann-Whitney tests.

(legend continued on next page)

well-established growth functions (*Igf2*, *Igf2r*, and *H19*) as model systems (importantly, due to imprinting, the mother is phenotypically normal). The analysis of these models of mismatch between fetal and placental growth allowed us to establish a number of mechanistic principles that regulate the cooperative signaling between the fetus and the placenta and, consequently, the control of maternal resources.

First, we found that circulating IGF2 reflects the growth rate of fetal tissues and the demand for nutrients. Mice with a severe decrease in levels of circulating/fetal IGF2 showed a disproportionate loss of FPECs. This severe placental angiogenesis phenotype was associated with reduced endothelial cell proliferation and increased apoptosis, altered differentiation of the underlying trophoblast and reduced density of MBS, ultimately leading to a failure in the expansion of the Lz and surface area for nutrient transport. Conversely, we show that greater fetal demands for growth, driven by bi-allelic *Igf2* expression, signals enhanced placental growth. Second, we also found that FPECs are a significant source of IGF2, with levels increasing with gestational age. Endothelial *Igf2*-deficient mice show impaired expansion of the microvasculature and Lz, but no disproportionate reduction in number of FPECs. These findings suggest that hormone-like signals from the fetus, such as IGF2, are also required for the normal expansion of the Lz and surface area of the placenta. We ruled out the hypothesis that failure in expansion is due to a reduction in the number of LaTP. IGF2 has been reported to be an essential component of maintenance of stem cell niches in other organs (Ferron et al., 2015; Ziegler et al., 2019). Our data suggest that endothelial IGF2 and circulating IGF2 are not required for the proliferation and maintenance of LaTP pools, but rather their differentiation.

We propose a model (Figure 7H) in which fetus-derived IGF2, from multiple tissues, is the signal that allows matching placental supply capacity to the fetal demands for growth. At the placental interface, circulating IGF2 directly stimulates endothelial cell proliferation and survival, and capillary branching in part through IGF2R. Circulating IGF2 may also directly control the growth and differentiation of the underlying trophoblast, as it can cross the capillary walls or permeate through the fenestrated endothelium (Bach, 2015). We suggest that the feto-placental endothelium is a large reservoir of IGF2, boosting further IGF2 signaling and acting in a paracrine and autocrine manner to control the growth and remodeling of FC, with a "secondary effect" on trophoblast morphogenesis. HCs that originate from precursors common to FPECs (Rhodes et al., 2008) do not play any significant role in Lz expansion. Importantly, the effect of IGF2 signaling on feto-placental microvascular remodeling seems specifically driven by fetus-derived IGF2, as we did not find evidence that IGF2 produced by the trophoblast has a direct role on vascularization, being instead required for trophoblast morphogenesis. We therefore suggest that the key role of circulating IGF2 is to provide fetus-derived angiogenic signals to promote the vascular tree expansion in later gestation, in conjunction with local IGF2, derived from the fetal endothelium of the placenta. Mechanistically, likely molecular triggers of fetus-derived IGF2 signaling on microvasculature expansion and trophoblast morphogenesis are IGF2R-ERK1/2-angiopoietin-Tie2/TEK signaling and the key trophoblast differentiation genes *Gcm1* and *Synb*, respectively. Activation of ERK1/2 signaling pathway by IGF2 via IGF2R has been observed *in vitro* in previous studies (El-Shewy et al., 2006, 2007). Additionally, primary aortic endothelial cells isolated from *Erk1*/*Erk2* double knockout mice associated transcriptional upregulation of several DEGs identified in the *Igf2*^{EpiKO} FPECs, including *Angpt2*, *Adamts1*, *Igtp*, and *Ifit1* (Srinivasan et al., 2009) (see Figure 5). Although the detailed mechanisms by which ERK1/2 signaling leads to changes in *Angpt2* expression remain to be elucidated, these observations are compatible with a model in which angiocrine IGF2 binds to IGF2R to activate ERK1/2 signaling pathway, leading to lower *Angpt2* expression, as well as other angiostatic factors (described in Figure 5). We found no evidence for de-regulation of known controllers of placenta angiogenesis, such as VEGF and PGF (Aplin et al., 2020) in our mouse models. Instead, the late gestation angiogenesis defects are related to angiopoietins, although the contribution of other pathways cannot be ruled out. Importantly, angiopoietin-Tie2/TEK signaling has also been implicated in trophoblast morphogenesis, independent of their vascular actions (Kappou et al., 2015) and therefore may be an important link between vascular effects and trophoblast in these models. Thus, our findings highlight the impact of the vasculature on trophoblast morphogenesis acting in late gestation.

Our study provides insights into the complex interplay between trophoblast branching morphogenesis and placental vascularization. We propose that IGF2 is a fetus-derived hormone-like molecule that signals to the placenta and adapts the expansion of feto-placental microvasculature and trophoblast morphogenesis to the embryo size. Matching placental supply capacity to fetal demand for growth also involves IGF2R—the other imprinted member of the IGF family (Constância et al., 2004). The imprinting of the IGF system is thus likely to have played a key evolutionary role in the origins of the expansion of the feto-placental microvasculature and surface area for nutrient transport throughout pregnancy—a fundamental biological process that is observed in all eutherian species (Fowden et al., 2006). In humans, circulating levels of IGF2 in the umbilical cord progressively increase between 29 weeks of gestation and term, similarly to our findings in the mouse (Gohlke et al., 2004). Additionally, large-for-gestational age and small-for-gestational age babies have been reported to show increased and reduced levels of IGF2 in the umbilical cord, respectively (Verhaeghe et al., 1993; Tzschoppe et al., 2015). Moreover, placenta obtained from imprinting growth syndrome patients with disrupted IGF2 signaling are often associated with placentomegaly in BWS cases, due to hypervascularization and hyperplasia (Aoki et al., 2011; Armes et al., 2012), but also small hypoplastic placentas in SRS cases (Yamazawa et al.,

(D) Volcano plot representation of DEGs identified by RNA-seq in E16 FPEC (*Igf2*^{ECKO} versus controls). Significant upregulated and downregulated DEGs (FDR < 0.05) are shown with red and blue, respectively.
(E) Transcription factors (TFs) identified by analysis of motif enrichment (AME).
(F) IPA regulatory network built with the four TFs identified using AME analysis. Proteins labeled with a star are known regulators of angiogenesis (angiostatic or pro-angiogenic factors) and key references are listed in Table S4. See also Figures S5C and S5D and Tables S3 and S4.

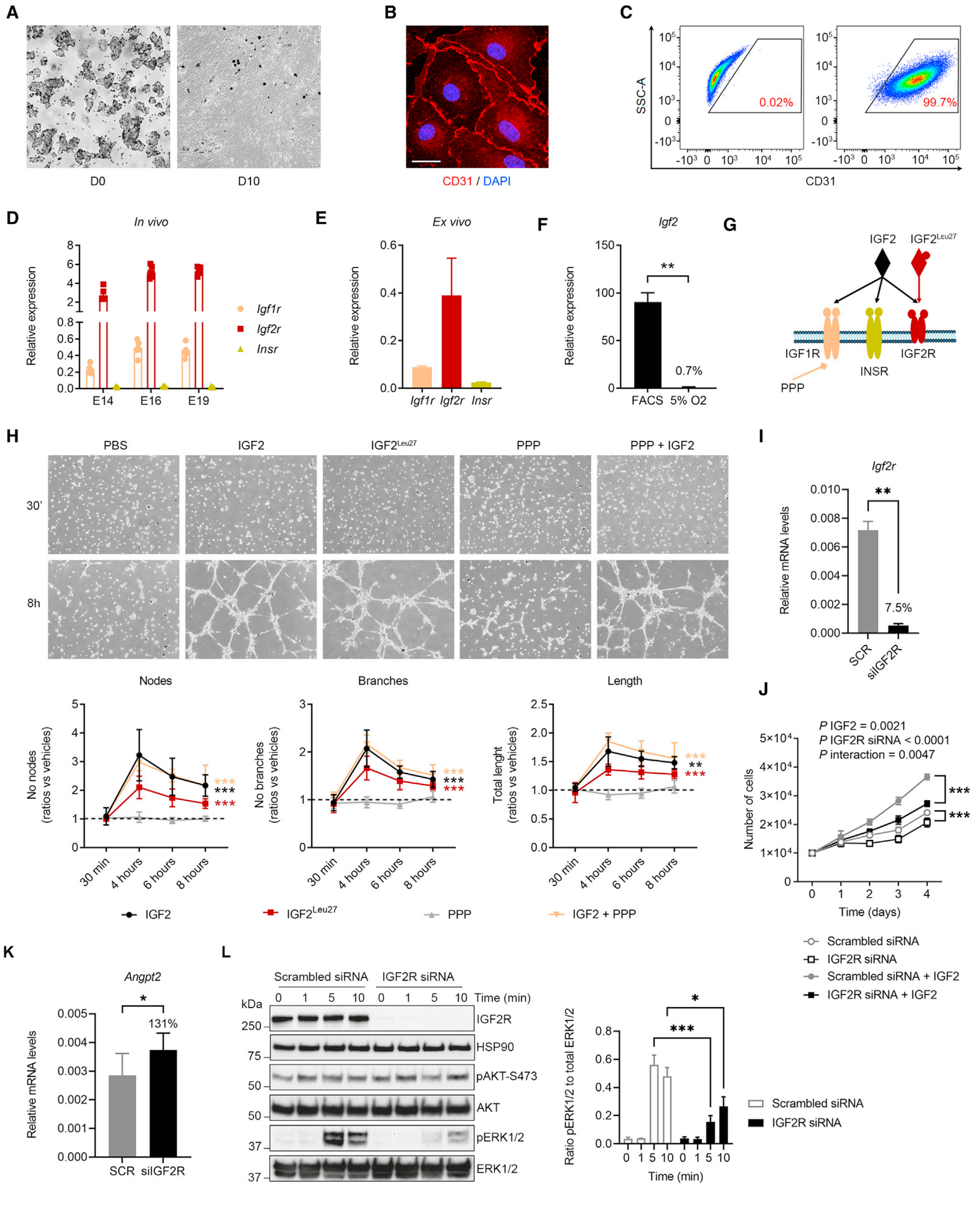

Figure 6. IGF2 Acts on FPECs via IGF2R-ERK signaling *ex vivo*

(A) Primary FPEC isolated from E16 Lz: D0—freshly isolated cells; D10—FPEC at passage one (P1, 10 days of culture).

(B) Confocal imaging of passage one FPEC, stained for CD31 (scale bars, 20 μm).

(C) Flow cytometry analysis of P1 FPEC stained for CD31, demonstrating that these are almost exclusively CD31$^+$.

(legend continued on next page)

2008), showing striking similarities to our mouse studies. Importantly, most cases of poor placentation in fetal growth restriction (FGR) reported so far were related to placental malperfusion from the maternal side and in response to a perturbed maternal environment (Mayhew et al., 2004). Our findings suggest that poor placentation in humans could be caused by deficient microvasculature expansion due to reduced fetus-derived IGF2 signaling, with important clinical implications.

Limitations of the study

One limitation is the inability to specifically target circulating levels of IGF2 without impacting on the size of the fetus. The source of circulating IGF2 is likely to be multi-organ, with a deletion of mesodermal *Igf2* enhancers resulting in reductions in circulating IGF2 by ~50% and severe FGR (Davies et al., 2002). Another limitation is the lack of Cre-lines to perform specific genetic manipulations in the placental endothelium. The *H19*-DMD^{EpiKO} and *Igf2r*^{ECKO} models are not without their limitations. Small contributions to the phenotypes due to the actions of the *H19*-encoded *miR-675* and the M6PR roles of IGF2R/activation of TGF-β1 cannot be completely ruled out. In our study, we also observed IGF2-independent effects of IGF2R *ex vivo*. However, endothelial-specific deletion of *Igf1r* is not associated with a phenotype, and *Insr*, and therefore INSR-IGF1R hybrids, are expressed at very low levels in endothelial cells and also unlikely to be functional. A major target of *mir-675* is IGF1R (Keniry et al., 2012), which is therefore unlikely to be of functional relevance in this context. Partial redundancy between actions of IGF1R and IGF2R in FPECs cannot be completely ruled out based on this study and warrants future experiments involving dual conditional deletions of *Igf1r* and *Igf2r* driven by *Tek*-Cre.

STAR★METHODS

Detailed methods are provided in the online version of this paper and include the following:

- KEY RESOURCES TABLE
- RESOURCE AVAILABILITY
  - Lead contact
  - Materials availability
  - Data and code availability
- EXPERIMENTAL MODEL AND SUBJECT DETAILS
  - Mice
- METHOD DETAILS
  - Plasma IGF2 measurements
  - *Igf2* mRNA *in situ* hybridization
  - Western blot analysis
  - Placenta stereology
  - Transmission electron microscopy
  - Immunostainings
  - qRT-PCR analysis
  - Expression microarray analysis
  - Flow cytometry analyses
  - Flow cytometry analysis of FPEC proliferation
  - FPEC and HC isolation by FACS
  - Primary FPEC isolation, culture, and tube formation assay
  - siRNA knockdown of *Igf2r*
  - RNA-sequencing and data analysis
- QUANTIFICATION AND STATISTICAL ANALYSIS

SUPPLEMENTAL INFORMATION

Supplemental information can be found online at https://doi.org/10.1016/j.devcel.2021.12.005.

AUTHOR CONTRIBUTIONS

I.S. and A.G. performed all of the *in vivo* experimental work, with contributions from A.H., J.L.-T., S.N.S., F.S., K.H., and A.N.S.-P. I.S., B.Y.H.L., and G.S.H.Y. performed bioinformatics analyses. I.S., M.R., and C.M.B. performed the *in vitro* tube formation assays. V.P.-G. performed the *in vitro Igf2r* knockdown experiments on primary placental endothelial cells. K.B. developed and performed the assay for IGF2 measurements in fetal plasma. I.S. and M.C. designed the project, and G.J.B., A.L.F., A.N.S-P., and C.M.B. assisted with the experimental design and data analysis and interpretation. I.S., G.J.B., and M.C. wrote the manuscript, with important contributions from A.L.F., C.M.B., and A.N.S.-P. All other authors discussed the results and edited the manuscript. M.C. managed and supervised all aspects of the study.

ACKNOWLEDGMENTS

We thank Matt Castle and Wendy Cooper for help with statistical analyses; Jeremy Skepper, Nuala Daw, Barbara Villela, and Bliss Anderson for technical assistance with placental stereology and transmission electron microscopy analyses; Adrian Wayman, Laura Hunter, and Edina Gulacsi for help with

(D) qRT-PCR analysis for *Igf1r*, *Igf2r*, and *Insr* in FPECs isolated by FACS (n = 6–7 per group).
(E) Relative expression of the three IGF receptors in P1 FPEC.
(F) qRT-PCR analysis of *Igf2* mRNA levels in P1 FPEC cultured in 5% O_2 versus primary FPEC isolated from E16 Lz by FACS.
(G) Schematic representation of IGF2 and IGF receptors. IGF2^{Leu27} analog acts specifically on IGF2R and picropodophyllin (PPP) inhibits phosphorylation of IGF1R.
(H) Representative images of capillary-like tube formation assay in primary FPEC seeded on matrigel and exposed to exogenous IGF2, IGF2^{Leu27}, PPP, or PPP+IGF2 (equal seeding of cell numbers at 30 min and tube formation at 8 h), and quantification of number of nodes, branches, and total length (n = 5–6 independent experiments).
(I) qRT-PCR analysis of *Igf2r* mRNA levels in primary FPECs upon knockdown by siRNA (n = 8 samples/group).
(J) Proliferation assay of primary FPEC with or without IGF2R siRNA knockdown, in presence or absence of IGF2, on 4 consecutive days after plating. Cells with IGF2R siRNA knockdown exhibit significant proliferation defects that are further accentuated upon IGF2 treatment (n = 5 biological replicates per group).
(K) qRT-PCR analysis of *Angpt2* mRNA levels in primary FPECs transfected with scrambled siRNA or IGF2R siRNA, upon 4 days of treatment with 50 ng/mL mouse recombinant IGF2 (n = 8 samples/group).
(L) Left side: identification of delayed ERK1/2 phosphorylation in FPECs with IGF2R siRNA knockdown upon acute treatment with 50 ng/mL mouse recombinant IGF2. HSP90 was used as internal control for protein loading. Right side: quantification of ratios pERK1/2 to total ERK1/2 for n = 3 independent biological replicates. For all graphs, data are presented as averages or individual values and error bars represent SEM. *p < 0.05, **p < 0.01, and ***p < 0.001 calculated by a Mann-Whitney test in (F), two-way ANOVA tests with Sidak's multiple comparisons test in (H), (J), and (L), Wilcoxon matched-pairs signed rank test in (I) and paired Student's t test in (K).

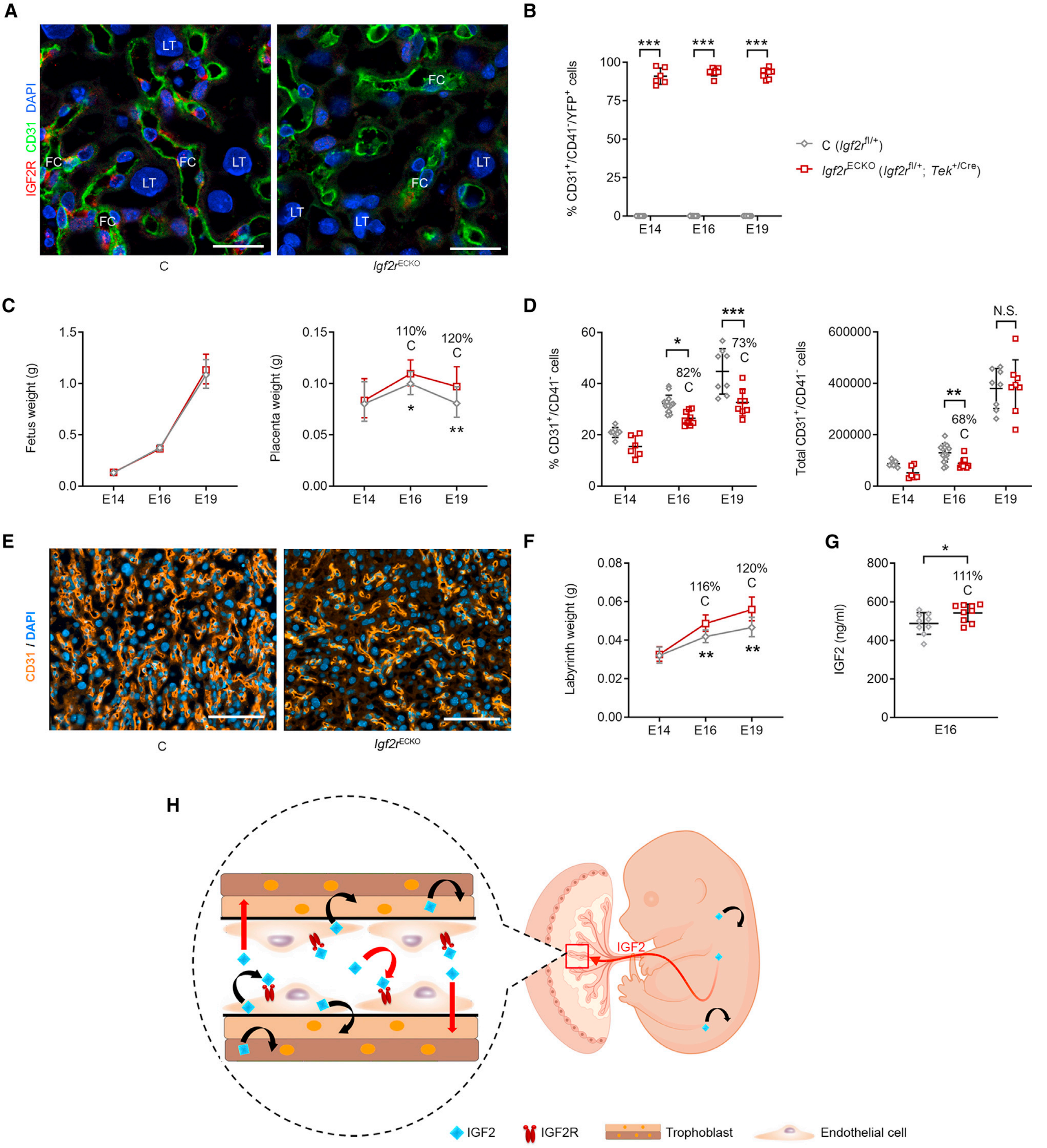

Figure 7. IGF2 acts on FPECs via IGF2R *in vivo*

(A) Representative double immunostaining for IGF2R (red) and CD31 (green) in *Igf2r*^{ECKO} mutant and control Lz at E16 (DAPI, blue; scale bars, 25 μm).

(B) Flow cytometry analysis showing that the majority (>80%) of *Igf2r*^{ECKO} mutant feto-placental endothelial cells (FPECs) express YFP (n = 6–14 per genotype).

(C) Fetal and placental growth kinetics in *Igf2r*^{ECKO} (*Igf2r*$^{fl/+}$; *Tek*$^{+/Cre}$) mutants compared with *Igf2r*$^{fl/+}$ controls (n = 8–28 conceptuses from n = 3–8 L for each developmental stage).

(D) Proportion and total numbers of FPEC/Lz measured by flow cytometry (n = 6–14 per group).

(E) Representative CD31 staining in E16 Lz (scale bars, 100 μm).

(F) Lz growth kinetics: *Igf2r*^{ECKO} (n = 8–16 conceptuses per group).

(legend continued on next page)

mouse husbandry; Keli Phillips and James Warner for help with preparing tissue samples for histology and F4/80 staining; Gregory Strachan for help with TUNEL+ and F4/80+ cells counting using HALO; Marcella Ma for help with preparing the RNA-seq libraries; Evgeniya Shmeleva and Francesco Colucci for advice regarding flow cytometry analyses and providing aliquots of several antibodies used for flow cytometry and FACS; Natalia Savinykh and Esther Perez for help with flow cytometry cell sorting. Schematic representations shown in the graphical abstract and in Figure 7H were generated using BioRender (Biorender.com). Funding: This work was supported by the Biotechnology and Biological Sciences Research Council (grant BB/H003312/1 to M.C.), the Medical Research Council (MRC_MC_UU_12012/4 to M.C.; MRC_MC_UU_12012/5 to the MRC Metabolic Diseases Unit; MR/R022690/1 to A.N.S.-P.), the Spanish Ministry of Science and Innovation (RYC-2019-026956 and PID2020-114459RA-I00 to V.P.-G.), the Wellcome Trust (Sir Henry Wellcome Postdoctoral Fellowship 220456/Z/20/Z to J.L.-T.), the Royal Society (Dorothy Hodgkin Research Fellowship grant DH130036 to A.N.S.-P.), the Centre for Trophoblast Research, and the NIHR Cambridge BRC Cell Phenotyping Hub.

DECLARATION OF INTERESTS

The authors declare no competing interests.

Received: December 20, 2019
Revised: September 30, 2021
Accepted: December 3, 2021
Published: December 27, 2021

REFERENCES

Angiolillo, A.L., Sgadari, C., Taub, D.D., Liao, F., Farber, J.M., Maheshwari, S., Kleinman, H.K., Reaman, G.H., and Tosato, G. (1995). Human interferon-inducible protein 10 is a potent inhibitor of angiogenesis *in vivo*. J. Exp. Med. *182*, 155–162. https://doi.org/10.1084/jem.182.1.155.

Angiolini, E., Coan, P.M., Sandovici, I., Iwajomo, O.H., Peck, G., Burton, G.J., Sibley, C.P., Reik, W., Fowden, A.L., and Constância, M. (2011). Developmental adaptations to increased fetal nutrient demand in mouse genetic models of Igf2-mediated overgrowth. FASEB J. *25*, 1737–1745. https://doi.org/10.1096/fj.10-175273.

Aoki, A., Shiozaki, A., Sameshima, A., Higashimoto, K., Soejima, H., and Saito, S. (2011). Beckwith-Wiedemann syndrome with placental chorangioma due to H19-differentially methylated region hypermethylation: a case report. J. Obstet. Gynaecol. Res. *37*, 1872–1876. https://doi.org/10.1111/j.1447-0756.2011.01654.x.

Aplin, J.D., Myers, J.E., Timms, K., and Westwood, M. (2020). Tracking placental development in health and disease. Nat. Rev. Endocrinol. *16*, 479–494. https://doi.org/10.1038/s41574-020-0372-6.

Armes, J.E., McGown, I., Williams, M., Broomfield, A., Gough, K., Lehane, F., and Lourie, R. (2012). The placenta in Beckwith-Wiedemann syndrome: genotype-phenotype associations, excessive extravillous trophoblast and placental mesenchymal dysplasia. Pathology *44*, 519–527. https://doi.org/10.1097/PAT.0b013e3283559c94.

Augustin, H.G., Koh, G.Y., Thurston, G., and Alitalo, K. (2009). Control of vascular morphogenesis and homeostasis through the angiopoietin-Tie system. Nat. Rev. Mol. Cell Biol. *10*, 165–177. https://doi.org/10.1038/nrm2639.

Austyn, J.M., and Gordon, S. (1981). F4/80, a monoclonal antibody directed specifically against the mouse macrophage. Eur. J. Immunol. *11*, 805–815. https://doi.org/10.1002/eji.1830111013.

Azzi, S., Abi Habib, W., and Netchine, I. (2014). Beckwith-Wiedemann and Russell-Silver Syndromes: from new molecular insights to the comprehension of imprinting regulation. Curr. Opin. Endocrinol. Diabetes Obes. *21*, 30–38. https://doi.org/10.1097/MED.0000000000000037.

Bach, L.A. (2015). Endothelial cells and the IGF system. J. Mol. Endocrinol. *54*, R1–R13. https://doi.org/10.1530/JME-14-0215.

Baker, J., Liu, J.P., Robertson, E.J., and Efstratiadis, A. (1993). Role of insulin-like growth factors in embryonic and postnatal growth. Cell *75*, 73–82. https://doi.org/10.1016/S0092-8674(05)80085-6.

Barlow, D.P., Stöger, R., Herrmann, B.G., Saito, K., and Schweifer, N. (1991). The mouse insulin-like growth factor type-2 receptor is imprinted and closely linked to the Tme locus. Nature *349*, 84–87. https://doi.org/10.1038/349084a0.

Beukers, M.W., Oh, Y., Zhang, H., Ling, N., and Rosenfeld, R.G. (1991). insulin-like growth factor II is highly selective for the type-II IGF receptor in binding, cross-linking and thymidine incorporation experiments. Endocrinology *128*, 1201–1203. https://doi.org/10.1210/endo-128-2-1201.

Branco-Price, C., Zhang, N., Schnelle, M., Evans, C., Katschinski, D.M., Liao, D., Ellies, L., and Johnson, R.S. (2012). Endothelial cell HIF-1α and HIF-2α differentially regulate metastatic success. Cancer Cell *21*, 52–65. https://doi.org/10.1016/j.ccr.2011.11.017.

Burns, J.L., and Hassan, A.B. (2001). Cell survival and proliferation are modified by insulin-like growth factor 2 between days 9 and 10 of mouse gestation. Development *128*, 3819–3830. https://doi.org/10.1242/dev.128.19.3819.

Coan, P.M., Ferguson-Smith, A.C., and Burton, G.J. (2004). Developmental dynamics of the definitive mouse placenta assessed by stereology. Biol. Reprod. *70*, 1806–1813. https://doi.org/10.1095/biolreprod.103.024166.

Coan, P.M., Ferguson-Smith, A.C., and Burton, G.J. (2005). Ultrastructural changes in the interhaemal membrane and junctional zone of the murine chorioallantoic placenta across gestation. J. Anat. *207*, 783–796. https://doi.org/10.1111/j.1469-7580.2005.00488.x.

Coan, P.M., Fowden, A.L., Constância, M., Ferguson-Smith, A.C., Burton, G.J., and Sibley, C.P. (2008). Disproportional effects of Igf2 knockout on placental morphology and diffusional exchange characteristics in the mouse. J. Physiol. *586*, 5023–5032. https://doi.org/10.1113/jphysiol.2008.157313.

Constância, M., Angiolini, E., Sandovici, I., Smith, P., Smith, R., Kelsey, G., Dean, W., Ferguson-Smith, A., Sibley, C.P., Reik, W., and Fowden, A. (2005). Adaptation of nutrient supply to fetal demand in the mouse involves interaction between the Igf2 gene and placental transporter systems. Proc. Natl. Acad. Sci. USA *102*, 19219–19224. https://doi.org/10.1073/pnas.0504468103.

Constância, M., Hemberger, M., Hughes, J., Dean, W., Ferguson-Smith, A., Fundele, R., Stewart, F., Kelsey, G., Fowden, A., Sibley, C., and Reik, W. (2002). Placental-specific IGF-II is a major modulator of placental and fetal growth. Nature *417*, 945–948. https://doi.org/10.1038/nature00819.

Constância, M., Kelsey, G., and Reik, W. (2004). Resourceful imprinting. Nature *432*, 53–57.

Davies, K., Bowden, L., Smith, P., Dean, W., Hill, D., Furuumi, H., Sasaki, H., Cattanach, B., and Reik, W. (2002). Disruption of mesodermal enhancers for Igf2 in the minute mutant. Development *129*, 1657–1668. https://doi.org/10.1242/dev.129.7.1657.

de Boer, J., Williams, A., Skavdis, G., Harker, N., Coles, M., Tolaini, M., Norton, T., Williams, K., Roderick, K., Potocnik, A.J., and Kioussis, D. (2003). Transgenic mice with hematopoietic and lymphoid specific expression of Cre. Eur. J. Immunol. *33*, 314–325. https://doi.org/10.1002/immu.200310005.

De Clercq, K., Lopez-Tello, J., Vriens, J., and Sferruzzi-Perri, A.N. (2020). Double-label immunohistochemistry to assess labyrinth structure of the mouse placenta with stereology. Placenta *94*, 44–47. https://doi.org/10.1016/j.placenta.2020.03.014.

(G) IGF2 levels (ng/mL) in plasma at E16 (n = 9 per group).
(H) Model summarizing the proposed actions of fetus-, endothelial-, and trophoblast-derived IGF2. For all graphs, data are presented as averages or individual values and error bars represent SD in (B), (D), and (G), or 95% CI in (C) and (F). N.S.— not significant; *p < 0.05; **p < 0.01; ***p < 0.001 calculated by two-way ANOVA tests in (B) and (D), mixed effects model in (C) and (F) and Mann-Whitney tests in (G). See also Figure S7 and Video S1.

DeChiara, T.M., Robertson, E.J., and Efstratiadis, A. (1991). Parental imprinting of the mouse insulin-like growth factor II gene. Cell *64*, 849–859. https://doi.org/10.1016/0092-8674(91)90513-x.

Dietrich, P., Dragatsis, I., Xuan, S., Zeitlin, S., and Efstratiadis, A. (2000). Conditional mutagenesis in mice with heat shock promoter-driven cre transgenes. Mamm. Genome *11*, 196–205. https://doi.org/10.1007/s003350010037.

El-Shewy, H.M., Johnson, K.R., Lee, M.H., Jaffa, A.A., Obeid, L.M., and Luttrell, L.M. (2006). Insulin-like growth factors mediate heterotrimeric G protein-dependent ERK1/2 activation by transactivating sphingosine 1-phosphate receptors. J. Biol. Chem. *281*, 31399–31407. https://doi.org/10.1074/jbc.M605339200.

El-Shewy, H.M., Lee, M.H., Obeid, L.M., Jaffa, A.A., and Luttrell, L.M. (2007). The insulin-like growth factor type 1 and insulin-like growth factor type 2/mannose-6-phosphate receptors independently regulate ERK1/2 activity in HEK293 cells. J. Biol. Chem. *282*, 26150–26157. https://doi.org/10.1074/jbc.M703276200.

Fehniger, T.A., and Caligiuri, M.A. (2001). Interleukin 15: biology and relevance to human disease. Blood *97*, 14–32. https://doi.org/10.1182/blood.v97.1.14.

Ferrón, S.R., Radford, E.J., Domingo-Muelas, A., Kleine, I., Ramme, A., Gray, D., Sandovici, I., Constancia, M., Ward, A., Menheniott, T.R., and Ferguson-Smith, A.C. (2015). Differential genomic imprinting regulates paracrine and autocrine roles of IGF2 in mouse adult neurogenesis. Nat. Commun. *6*, 8265. https://doi.org/10.1038/ncomms9265.

Fischer, A., Schumacher, N., Maier, M., Sendtner, M., and Gessler, M. (2004). The Notch target genes Hey1 and Hey2 are required for embryonic vascular development. Genes Dev. *18*, 901–911. https://doi.org/10.1101/gad.291004.

Fowden, A.L., Ward, J.W., Wooding, F.P., Forhead, A.J., and Constância, M. (2006). Programming placental nutrient transport capacity. J. Physiol. *572*, 5–15. https://doi.org/10.1113/jphysiol.2005.104141.

Gardner, R.L., Squire, S., Zaina, S., Hills, S., and Graham, C.F. (1999). Insulin-like growth factor-2 regulation of conceptus composition: effects of the trophectoderm and inner cell mass genotypes in the mouse. Biol. Reprod. *60*, 190–195. https://doi.org/10.1095/biolreprod60.1.190.

Ghosh, P., Dahms, N.M., and Kornfeld, S. (2003). Mannose 6-phosphate receptors: new twists in the tale. Nat. Rev. Mol. Cell Biol. *4*, 202–212. https://doi.org/10.1038/nrm1050.

Girnita, A., Girnita, L., del Prete, F., Bartolazzi, A., Larsson, O., and Axelson, M. (2004). Cyclolignans as inhibitors of the insulin-like growth factor-1 receptor and malignant cell growth. Cancer Res. *64*, 236–242. https://doi.org/10.1158/0008-5472.can-03-2522.

Gohlke, B.C., Fahnenstich, H., Dame, C., and Albers, N. (2004). Longitudinal data for intrauterine levels of fetal IGF-I and IGF-II. Horm. Res. *61*, 200–204. https://doi.org/10.1159/000076552.

Gregory, J.L., Morand, E.F., McKeown, S.J., Ralph, J.A., Hall, P., Yang, Y.H., McColl, S.R., and Hickey, M.J. (2006). Macrophage migration inhibitory factor induces macrophage recruitment via CC chemokine ligand 2. J. Immunol. *177*, 8072–8079. https://doi.org/10.4049/jimmunol.177.11.8072.

Hammerle, C.M., Sandovici, I., Brierley, G.V., Smith, N.M., Zimmer, W.E., Zvetkova, I., Prosser, H.M., Sekita, Y., Lam, B.Y.H., Ma, M., et al. (2020). Mesenchyme-derived IGF2 is a major paracrine regulator of pancreatic growth and function. PLoS Genet *16*, e1009069. https://doi.org/10.1371/journal.pgen.1009069.

Harris, L.K., and Westwood, M. (2012). Biology and significance of signalling pathways activated by IGF-II. Growth Factors *30*, 1–12. https://doi.org/10.3109/08977194.2011.640325.

Hughes, J., Surakhy, M., Can, S., Ducker, M., Davies, N., Szele, F., Bühnemann, C., Carter, E., Trikin, R., Crump, M.P., et al. (2019). Maternal transmission of an Igf2r domain 11: IGF2 binding mutant allele (Igf2rI1565A) results in partial lethality, overgrowth and intestinal adenoma progression. Sci. Rep. *9*, 11388. https://doi.org/10.1038/s41598-019-47827-9.

Hughes, M., Natale, B.V., Simmons, D.G., and Natale, D.R. (2013). Ly6e expression is restricted to syncytiotrophoblast cells of the mouse placenta. Placenta *34*, 831–835. https://doi.org/10.1016/j.placenta.2013.05.011.

Kappou, D., Sifakis, S., Konstantinidou, A., Papantoniou, N., and Spandidos, D.A. (2015). Role of the angiopoietin/Tie system in pregnancy (Review). Exp. Ther. Med. *9*, 1091–1096. https://doi.org/10.3892/etm.2015.2280.

Keniry, A., Oxley, D., Monnier, P., Kyba, M., Dandolo, L., Smits, G., and Reik, W. (2012). The H19 lincRNA is a developmental reservoir of miR-675 that suppresses growth and Igf1r. Nat. Cell Biol. *14*, 659–665. https://doi.org/10.1038/ncb2521.

Kim, D., Pertea, G., Trapnell, C., Pimentel, H., Kelley, R., and Salzberg, S.L. (2013). TopHat2: accurate alignment of transcriptomes in the presence of insertions, deletions and gene fusions. Genome Biol. *14*, R36. https://doi.org/10.1186/gb-2013-14-4-r36.

Kisanuki, Y.Y., Hammer, R.E., Miyazaki, J., Williams, S.C., Richardson, J.A., and Yanagisawa, M. (2001). Tie2-Cre transgenic mice: a new model for endothelial cell-lineage analysis *in vivo*. Dev. Biol. *230*, 230–242. https://doi.org/10.1006/dbio.2000.0106.

Lankhorst, S., Danser, A.H., and van den Meiracker, A.H. (2016). Endothelin-1 and antiangiogenesis. Am. J. Physiol. Regul. Integr. Comp. Physiol. *310*, R230–R234. https://doi.org/10.1152/ajpregu.00373.2015.

Lau, M.M., Stewart, C.E., Liu, Z., Bhatt, H., Rotwein, P., and Stewart, C.L. (1994). Loss of the imprinted IGF2/cation-independent mannose 6-phosphate receptor results in fetal overgrowth and perinatal lethality. Genes Dev. *8*, 2953–2963. https://doi.org/10.1101/gad.8.24.2953.

Lawler, P.R., and Lawler, J. (2012). Molecular basis for the regulation of angiogenesis by thrombospondin-1 and -2. Cold Spring Harb. Perspect. Med. *2*, a006627. https://doi.org/10.1101/cshperspect.a006627.

Lee, N.V., Sato, M., Annis, D.S., Loo, J.A., Wu, L., Mosher, D.F., and Iruela-Arispe, M.L. (2006). ADAMTS1 mediates the release of antiangiogenic polypeptides from TSP1 and 2. EMBO J. *25*, 5270–5283. https://doi.org/10.1038/sj.emboj.7601400.

Livak, K.J., and Schmittgen, T.D. (2001). Analysis of relative gene expression data using real-time quantitative PCR and the 2(-Delta Delta C(T)) method. Methods *25*, 402–408. https://doi.org/10.1006/meth.2001.1262.

Madisen, L., Zwingman, T.A., Sunkin, S.M., Oh, S.W., Zariwala, H.A., Gu, H., Ng, L.L., Palmiter, R.D., Hawrylycz, M.J., Jones, A.R., et al. (2010). A robust and high-throughput Cre reporting and characterization system for the whole mouse brain. Nat. Neurosci. *13*, 133–140. https://doi.org/10.1038/nn.2467.

Maeng, Y.-S., Choi, H.-J., Kwon, J.-Y., Park, Y.-W., Choi, K.-S., Min, J.-K., Kim, Y.-H., Suh, P.-G., Kang, K.-S., Won, M.-H., et al. (2009). Endothelial progenitor cell homing: prominent role of the IGF2-IGF2R-PLCbeta2 axis. Blood *113*, 233–243. https://doi.org/10.1182/blood-2008-06-162891.

Mayhew, T.M., Charnock-Jones, D.S., and Kaufmann, P. (2004). Aspects of human fetoplacental vasculogenesis and angiogenesis. III. Changes in complicated pregnancies. Placenta *25*, 127–139. https://doi.org/10.1016/j.placenta.2003.10.010.

Miner, J.H., Cunningham, J., and Sanes, J.R. (1998). Roles for laminin in embryogenesis: exencephaly, syndactyly, and placentopathy in mice lacking the laminin α5 chain. J. Cell Biol. *143*, 1713–1723. https://doi.org/10.1083/jcb.143.6.1713.

Monk, D., Arnaud, P., Apostolidou, S., Hills, F.A., Kelsey, G., Stanier, P., Feil, R., and Moore, G.E. (2006). Limited evolutionary conservation of imprinting in the human placenta. Proc. Natl. Acad. Sci. USA *103*, 6623–6628. https://doi.org/10.1073/pnas.0511031103.

Nagai, A., Takebe, K., Nio-Kobayashi, J., Takahashi-Iwanaga, H., and Iwanaga, T. (2010). Cellular expression of the monocarboxylate transporter (MCT) family in the placenta of mice. Placenta *31*, 126–133. https://doi.org/10.1016/j.placenta.2009.11.013.

Okamoto, T., Katada, T., Murayama, Y., Ui, M., Ogata, E., and Nishimoto, I. (1990). A simple structure encodes G protein-activating function of the IGF-II/mannose 6-phosphate receptor. Cell *62*, 709–717. https://doi.org/10.1016/0092-8674(90)90116-V.

Oudejans, C.B., Westerman, B., Wouters, D., Gooyer, S., Leegwater, P.A., van Wijk, I.J., and Sleutels, F. (2001). Allelic IGF2R repression does not correlate with expression of antisense RNA in human extraembryonic tissues. Genomics *73*, 331–337. https://doi.org/10.1006/geno.2001.6522.

Pérez-García, V., Redondo-Muñoz, J., Kumar, A., and Carrera, A.C. (2014). Cell activation-induced phosphoinositide 3-kinase alpha/beta dimerization regulates PTEN activity. Mol. Cell. Biol. *34*, 3359–3373. https://doi.org/10.1128/MCB.00167-14.

Rawn, S.M., and Cross, J.C. (2008). The evolution, regulation, and function of placenta-specific genes. Annu. Rev. Cell Dev. Biol. *24*, 159–181. https://doi.org/10.1146/annurev.cellbio.24.110707.175418.

Rhodes, K.E., Gekas, C., Wang, Y., Lux, C.T., Francis, C.S., Chan, D.N., Conway, S., Orkin, S.H., Yoder, M.C., and Mikkola, H.K. (2008). The emergence of hematopoietic stem cells is initiated in the placental vasculature in the absence of circulation. Cell Stem Cell *2*, 252–263. https://doi.org/10.1016/j.stem.2008.01.001.

Schneider, C.A., Rasband, W.S., and Eliceiri, K.W. (2012). NIH Image to ImageJ: 25 years of image analysis. Nat. Methods *9*, 671–675. https://doi.org/10.1038/nmeth.2089.

Schwenk, F., Baron, U., and Rajewsky, K. (1995). A cre-transgenic mouse strain for the ubiquitous deletion of loxP-flanked gene segments including deletion in germ cells. Nucleic Acids Res. *23*, 5080–5081. https://doi.org/10.1093/nar/23.24.5080.

Sferruzzi-Perri, A.N., Sandovici, I., Constância, M., and Fowden, A.L. (2017). Placental phenotype and the insulin-like growth factors: resource allocation to fetal growth. J. Physiol. *595*, 5057–5093. https://doi.org/10.1113/JP273330.

Simmons, D.G., Rawn, S., Davies, A., Hughes, M., and Cross, J.C. (2008). Spatial and temporal expression of the 23 murine Prolactin/Placental Lactogen-related genes is not associated with their position in the locus. BMC Genomics *9*, 352. https://doi.org/10.1186/1471-2164-9-352.

Srinivas, S., Watanabe, T., Lin, C.S., William, C.M., Tanabe, Y., Jessell, T.M., and Costantini, F. (2001). Cre reporter strains produced by targeted insertion of EYFP and ECFP into the ROSA26 locus. BMC Dev. Biol. *1*, 4. https://doi.org/10.1186/1471-213x-1-4.

Srinivasan, R., Zabuawala, T., Huang, H., Zhang, J., Gulati, P., Fernandez, S., Karlo, J.C., Landreth, G.E., Leone, G., and Ostrowski, M.C. (2009). Erk1 and Erk2 regulate endothelial cell proliferation and migration during mouse embryonic angiogenesis. PLoS One *4*, e8283. https://doi.org/10.1371/journal.pone.0008283.

Srivastava, M., Hsieh, S., Grinberg, A., Williams-Simons, L., Huang, S.P., and Pfeifer, K. (2000). H19 and Igf2 monoallelic expression is regulated in two distinct ways by a shared cis acting regulatory region upstream of H19. Genes Dev. *14*, 1186–1195. https://doi.org/10.1101/gad.14.10.1186.

Supek, F., Bošnjak, M., Škunca, N., and Šmuc, T. (2011). REVIGO summarizes and visualizes long lists of gene ontology terms. PLoS One *6*, e21800. https://doi.org/10.1371/journal.pone.0021800.

Tallquist, M.D., and Soriano, P. (2000). Epiblast-restricted Cre expression in MORE mice: a tool to distinguish embryonic vs. extra-embryonic gene function. Genesis *26*, 113–115. https://doi.org/10.1002/(sici)1526-968x(200002)26:2<113::aid-gene3>3.0.co;2-2.

Trapnell, C., Williams, B.A., Pertea, G., Mortazavi, A., Kwan, G., van Baren, M.J., Salzberg, S.L., Wold, B.J., and Pachter, L. (2010). Transcript assembly and quantification by RNA-seq reveals unannotated transcripts and isoform switching during cell differentiation. Nat. Biotechnol. *28*, 511–515. https://doi.org/10.1038/nbt.1621.

Tzschoppe, A., Riedel, C., von Kries, R., Struwe, E., Rascher, W., Dörr, H.G., Beckmann, M.W., Schild, R.L., Goecke, T.W., Flyvbjerg, A., et al. (2015). Differential effects of low birthweight and intrauterine growth restriction on umbilical cord blood insulin-like growth factor concentrations. Clin. Endocrinol. *83*, 739–745. https://doi.org/10.1111/cen.12844.

Ueno, M., Lee, L.K., Chhabra, A., Kim, Y.J., Sasidharan, R., Van Handel, B., Wang, Y., Kamata, M., Kamran, P., Sereti, K.-I., et al. (2013). c-Met-dependent multipotent labyrinth trophoblast progenitors establish placental exchange interface. Dev. Cell *27*, 373–386. https://doi.org/10.1016/j.devcel.2013.10.019.

Uthaiah, R.C., Praefcke, G.J., Howard, J.C., and Herrmann, C. (2003). IIGP1, an interferon-gamma-inducible 47-kDa GTPase of the mouse, showing cooperative enzymatic activity and GTP-dependent multimerization. J. Biol. Chem. *278*, 29336–29343. https://doi.org/10.1074/jbc.M211973200.

Verhaeghe, J., Van Bree, R., Van Herck, E., Laureys, J., Bouillon, R., and Van Assche, F.A. (1993). C-peptide, insulin-like growth factors I and II, and insulin-like growth factor binding protein-1 in umbilical cord serum: correlations with birth weight. Am. J. Obstet. Gynecol. *169*, 89–97. https://doi.org/10.1016/0002-9378(93)90137-8.

Vidal, A., and Koff, A. (2000). Cell-cycle inhibitors: three families united by a common cause. Gene *247*, 1–15. https://doi.org/10.1016/s0378-1119(00)00092-5.

Wang, Z.Q., Fung, M.R., Barlow, D.P., and Wagner, E.F. (1994). Regulation of embryonic growth and lysosomal targeting by the imprinted Igf2/Mpr gene. Nature *372*, 464–467. https://doi.org/10.1038/372464a0.

Wei, Y., Su, J., Liu, H., Lv, J., Wang, F., Yan, H., Wen, Y., Liu, H., Wu, Q., and Zhang, Y. (2014). MetaImprint: an information repository of mammalian imprinted genes. Development *141*, 2516–2523. https://doi.org/10.1242/dev.105320.

Wenzel, P.L., and Leone, G. (2007). Expression of Cre recombinase in early diploid trophoblast cells of the mouse placenta. Genesis *45*, 129–134. https://doi.org/10.1002/dvg.20276.

Woods, L., Perez-Garcia, V., Kieckbusch, J., Wang, X., DeMayo, F., Colucci, F., and Hemberger, M. (2017). Decidualisation and placentation defects are a major cause of age-related reproductive decline. Nat. Commun. *8*, 352. https://doi.org/10.1038/s41467-017-00308-x.

Wylie, A.A., Pulford, D.J., McVie-Wylie, A.J., Waterland, R.A., Evans, H.K., Chen, Y.T., Nolan, C.M., Orton, T.C., and Jirtle, R.L. (2003). Tissue-specific inactivation of murine M6P/IGF2R. Am. J. Pathol. *162*, 321–328. https://doi.org/10.1016/S0002-9440(10)63823-0.

Xu, Y., Goodyer, C.G., Deal, C., and Polychronakos, C. (1993). Functional polymorphism in the parental imprinting of the human IGF2R gene. Biochem. Biophys. Res. Commun. *197*, 747–754. https://doi.org/10.1006/bbrc.1993.2542.

Yamazawa, K., Kagami, M., Nagai, T., Kondoh, T., Onigata, K., Maeyama, K., Hasegawa, T., Hasegawa, Y., Yamazaki, T., Mizuno, S., et al. (2008). Molecular and clinical findings and their correlations in Silver-Russell syndrome: implications for a positive role of IGF2 in growth determination and differential imprinting regulation of the IGF2-H19 domain in bodies and placentas. J. Mol. Med. *86*, 1171–1181. https://doi.org/10.1007/s00109-008-0377-4.

Ziegler, A.N., Feng, Q., Chidambaram, S., Testai, J.M., Kumari, E., Rothbard, D.E., Constancia, M., Sandovici, I., Cominski, T., Pang, K., et al. (2019). Insulin-like growth factor II: an essential adult stem cell niche constituent in brain and intestine. Stem Cell Rep *12*, 816–830. https://doi.org/10.1016/j.stemcr.2019.02.011.

STAR★METHODS

KEY RESOURCES TABLE

Reagent or resource	Source	Identifier
Antibodies		
Rat anti-mouse IGF-II	R&D Systems	RRID: AB_2122524
Biotinylated goat anti-mouse IGF-II	R&D Systems	RRID: AB_2122525
Anti-DIG-AP antibody	Roche	RRID: AB_2734716
Goat anti-human IGF2	R&D Systems	RRID: AB_354449
Goat anti-mouse SOD1	R&D Systems	RRID: AB_2239724
Rabbit anti-goat IgG-HRP	Santa Cruz	RRID: AB_656964
Goat anti-GFP	Abcam	RRID: AB_305643
Rabbit anti-CD31	Abcam	RRID: AB_726362
Goat anti-CD31	R&D Systems	RRID: AB_2161028
Rat anti-mouse F4/80	Bio-Rad	RRID: AB_2098196
Chicken anti-MCT1	Merk Millipore	RRID: AB_90565
Rabbit anti-MCT4	Merk Millipore	RRID: AB_2286063
Rabbit anti-laminin	Dako	RRID: AB_2313665
Rabbit anti-pan cytokeratin	Novus Biologicals	RRID: AB_2296857
Rat anti-mouse CD326/Epcam	BD Biosciences	RRID: AB_394370
Donkey anti-goat-AF488	Jackson ImmunoResearch	RRID: AB_2340430
Donkey anti-rabbit-AF594	Jackson ImmunoResearch	RRID: AB_2340619
Goat anti-rabbit, biotinylated	Abcam	RRID: AB_954902
NL557-conjugated donkey anti-goat	R&D Systems	RRID: AB_663766)
Rabbit anti-rat	Bethyl	RRID: AB_10681533
Anti-rabbit HRP	Vector Labs	RRID: AB_2631198
Donkey anti-chicken-AF488	Jackson ImmunoResearch	RRID: AB_2340376
Goat anti-rabbit-AP	Abcam	RRID: AB_954595
Donkey anti-rat-AF594	Thermo Fisher Scientific	RRID: AB_2535795
Rat anti-CD16/32	BioLegend	RRID: AB_1574975
Rat anti-mouse CD41-PE	BioLegend	RRID: AB_2129745
Rat anti-mouse CD31-AF647	BioLegend	RRID: AB_2161029
Rat anti-mouse CD326-AF647	BioLegend	RRID: AB_1134101
Rat anti-mouse CD41-BV421	BioLegend	RRID: AB_10960744
Rat anti-mouse CD117-PE	BioLegend	RRID: AB_313217
Rat anti-mouse Ly-6A/E-BV510	BioLegend	RRID: AB_2561593
Rat anti-lineage cocktail-BV421	BioLegend	RRID: AB_11203535
Rat anti-mouse CD45-V500	BD Biosciences	RRID: AB_10697046
Rat anti-mouse CD34-AF700	Thermo Fisher Scientific	RRID: AB_493998
Rabbit anti IGF2R	Cell Signaling	RRID: AB_2798462
Mouse anti-Phospho-p44/42 MAPK (Erk1/2) (Thr202/Tyr204)	Cell Signaling	RRID: AB_331768
Rabbit anti-p44/42 MAPK (Erk1/2)	Cell Signaling	RRID: AB_330744
Rabbit anti-Phospho-Akt (Ser473)	Cell Signaling	RRID: AB_329825
Rabbit anti-Akt	Cell Signaling	RRID: AB_329827
Rabbit anti-HSP90	Cell Signaling	RRID: AB_2233307
Chemicals, peptides, and recombinant proteins		
RIPA buffer	Sigma	R0278

(*Continued on next page*)

Continued

Reagent or resource	Source	Identifier
Streptavidin Sulpho-TAG	MSD	R32AD-1
Biotinylated lectin	Vector Laboratories	RRID: AB_2314661
pCR2.1-TOPO plasmid	Thermo Fisher Scientific	K450002
Digoxigenin	Roche	11175025910
Blocking reagent	Roche	1096176001
BCIP/NBT mix	Promega	S3771
Pierce BCA Assay Protein kit	Thermo Fisher Scientific	23225
12-well NuPAGE Novex 4–12% Bis-Tris precast gels	Thermo Fisher Scientific	NP0322BOX
Novex Sharp protein standard	Invitrogen	LC5800
iBlot Transfer Stacks	Invitrogen	IB3010-01
Clarity ECL Western Blotting Substrate	Bio-Rad	1705060
Stripping buffer	Thermo Fisher Scientific	21059
DAPI	Sigma	D9542
RNeasy Plus Micro Kit	Qiagen	74034
RNeasy Plus Mini Kit	Qiagen	74134
RNeasy Midi Kits	Qiagen	75144
RNA 6000 Pico Kit	Agilent	5067-1513
RNA 6000 Nano Kit	Agilent	5067-1511
RevertAid RT Reverse Transcription Kit	Thermo Fisher Scientific	K1622
SYBR Green JumpStart Taq Ready Mix	Sigma	S4438
RBC lysis buffer	BioLegend	420301
Staining buffer	BioLegend	420201
7-Aminoactinomycin	Invitrogen	A1310
5-ethynyl-2′-deoxyuridine	Thermo Fisher Scientific	A10044
Red LIVE/DEAD Fixable Dead Cell Stain	Thermo Fisher Scientific	L23102
Collagenase type I	Sigma	SCR103
Endothelial mitogens	Sigma	E2759
Serum replacement media	Sigma	S0638
Picropodophyllotoxin	Sigma	T9576
Accutase	Sigma	A6964
Angiogenesis μ-Slides	Ibidi	81506
Matrigel	BD Biosciences	354234
Critical commercial assays		
Mouse IGF-II DuoSet ELISA kit	R&D Systems	RRID:AB_2884002
In Situ Cell Death Detection Kit, TMR red	Sigma	012156792910
TUNEL Assay Kit – BrdU-Red	Abcam	ab66110
Click-iT EdU Alexa Fluor 488 Imaging Kit	Invitrogen	C10337
Mouse Gene 1.0 ST Array	Affymetrix	901171
Click-iT EdU Alexa Fluor 488 Flow Cytometry Assay Kit	Thermo Fisher Scientific	C10420
Mouse recombinant IGF2	R&D Systems	792-MG-050
Human IGF2^{Leu27}	GroPep	TU100
Complete endothelial growth medium	Cell Biologics	M1168
Basal endothelial growth medium	Cell Biologics	M1168b
Stealth siRNA for *Igf2r*	Thermo Fisher Scientific	1320003
Stealth scrambled siRNA	Thermo Fisher Scientific	12935100
Lipofectamine RNAiMax transfection reagent	Thermo Fisher Scientific	13778075

(*Continued on next page*)

Continued		
Reagent or resource	Source	Identifier
Deposited data		
Processed gene expression data from expression microarray and RNA-seq obtained in the *Igf2*^{EpiKO} model	This paper	GSE125434
Processed gene expression data from RNA-seq obtained in the *Igf2*^{ECKO} model	This paper	GSE179549
Experimental models: Cell lines		
primary placental microvascular endothelial cells isolated from C57BL/6J mice	Cell Biologics	C57-6056
Experimental models: Organisms/strains		
Mouse *Igf2*$^{fl/fl}$	Hammerle et al., 2020	N/A
Mouse *Meox2*^{Cre}	Tallquist and Soriano, 2000	The Jackson Laboratory (Stock No: 003755)
Mouse *Tek*^{Cre}	Kisanuki et al., 2001	The Jackson Laboratory (Stock No: 008863)
Mouse *Cyp19*^{Cre}	Wenzel and Leone, 2007	N/A
Mouse *CMV*^{Cre}	Schwenk et al., 1995	The Jackson Laboratory (Stock No: 006054)
Mouse *Vav*^{iCre}	de Boer et al., 2003	The Jackson Laboratory (Stock No: 008610)
Mouse *Rosa26* ^{fl}STOP^{fl}YFP	Srinivas et al., 2001	The Jackson Laboratory (Stock No: 006148)
Mouse Ai9(RCL-tdT)	Madisen et al., 2010	The Jackson Laboratory (Stock No: 007909)
Mouse *H19*-DMD$^{fl/fl}$	Srivastava et al., 2000	N/A
Mouse *Igf1r*$^{fl/fl}$	Dietrich et al., 2000	The Jackson Laboratory (Stock No: 012251)
Mouse *Igf2r*$^{fl/fl}$	Wylie et al., 2003	N/A
Mouse: C57BL/6J	Charles River Laboratories	Charles River Laboratories (Strain Code 632)
Oligonucleotides		
Primers used for genotyping or qRT-PCR, see Table S6	This paper	Sigma Aldrich (Merck)
Software and algorithms		
GraphPad Prism 8 software	GraphPad	https://www.graphpad.com/
R3.3	R Foundation	https://www.r-project.org/
MSD Workbench Software	MSD	https://www.mesoscale.com
ImageLab	Bio-Rad	https://www.bio-rad.com
NewCAST	Visiopharm	https://visiopharm.com
ZEN 2009	Carl Zeiss	https://www.zeiss.com/microscopy
Volocity 6.3	Improvision	https://www.perkinelmer.com
HALO	PerkinElmer	https://www.perkinelmer.com
GeneSpring GX 12.1	Agilent	https://www.agilent.com
Ingenuity Pathway Analysis	Qiagen	https://digitalinsights.qiagen.com
FlowJo v.10	TreeStar	https://www.flowjo.com
Angiogenesis Analyzer	Schneider et al., 2012	https://imagej.nih.gov/ij/
TopHat 2.0.11	Kim et al., 2013	https://ccb.jhu.edu/software/tophat
Cufflinks 2.2.1	Trapnell et al., 2010	https://github.com/cole-trapnell-lab/cufflinks
DAVID v6.8	LHRI	https://david.ncifcrf.gov/
REViGO	Supek et al., 2011	http://revigo.irb.hr
EPD	Swiss Institute of Bioinformatics	https://epd.vital-it.ch/index.php
AME v4.12.0	MEME Suite	http://meme-suite.org

RESOURCE AVAILABILITY

Lead contact

Further information and requests for resources and reagents should be directed to and will be fulfilled by the lead contact, Miguel Constância (jmasmc2@cam.ac.uk).

Materials availability

All materials generated in this study are available from the lead contact without restriction.

Data and code availability

- Processed gene expression data from expression microarray and RNA-seq comparisons are available in Tables S1, S2, and S3 and the corresponding raw data have been deposited in the Gene Expression Omnibus (GEO) under the accession numbers GSE125434 and GSE179549.
- This paper does not report original code.
- Any additional information required to reanalyze the data reported in this work paper is available from the lead contact upon request.

EXPERIMENTAL MODEL AND SUBJECT DETAILS

Mice

Mice were bred, maintained, and mated under pathogen-free conditions at the University of Cambridge Phenomics Unit (West Forvie), in accordance with the University of Cambridge Animal Welfare and Ethical Review Body and the United Kingdom Home Office Regulations. The morning of the copulation plug discovery was counted as embryonic day 1 (E1).

The *Igf2*$^{fl/fl}$ mice were generated in our laboratory (Hammerle et al., 2020). *Meox2*^{Cre} mice (Tallquist and Soriano, 2000), *Tek*^{Cre} mice (Kisanuki et al., 2001), *CMV*^{Cre} mice (Schwenk et al., 1995) and *Igf1r*$^{fl/fl}$ mice (Dietrich et al., 2000) were imported from the Jackson Laboratory (Maine, USA). *Meox2*^{Cre} is active starting at E5 in the epiblast, which gives rise to the entire embryo proper and FPEC (Tallquist and Soriano, 2000). *Tek*^{Cre} (also known as *Tie2*^{Cre}) activity starts at E7.5 in the endothelial cell lineage, including FPEC (Kisanuki et al., 2001). *CMV*^{Cre} activity starts soon after fertilization and induces ubiquitous deletion of floxed alleles in all tissues, including the germline (Schwenk et al., 1995). *Cyp19*^{Cre} mice (Wenzel and Leone, 2007) were kindly provided by Prof. Gustavo Leone (Medical University of South Carolina). *Cyp19*^{Cre} is active from E6.5 in the early diploid trophoblast cells that give rise to spongiotrophoblast, giant cells, and labyrinthine trophoblast cells (Wenzel and Leone, 2007). *Vav*^{iCre} mice, in which expression of an optimized variant of Cre is expressed in all hematopoietic cells but not in endothelial cells (de Boer et al., 2003), were kindly provided by Dr. Bidesh Mahata (University of Cambridge). (*Rosa26* ^{fl}STOP^{fl}YFP mice (Srinivas et al., 2001) were kindly provided by Dr. Martin Turner (The Babraham Institute, Cambridge), Ai9(RCL-tdT) mice (Madisen et al., 2010) by Prof. William Colledge (University of Cambridge), *H19*-DMD$^{fl/fl}$ mice (Srivastava et al., 2000) and *Igf2r*$^{fl/fl}$ mice (Wylie et al., 2003) by Prof. Bass Hassan (University of Oxford). Deletion of *H19*-DMD leads to reactivation of the silent maternal *Igf2* allele, as well as down-regulation of *H19* mRNA levels (Srivastava et al., 2000).

All strains were bred into an inbred C57BL/6J genetic background for >10 generations, with the exception of *Vav*^{iCre} strain that was maintained on an inbred C57BL/6N genetic background. For all crosses (Table S5), the parent transmitting the floxed allele was also homozygous for the *Rosa26* ^{fl}STOP^{fl}YFP allele. Thus, YFP expression provided an internal control for efficiency of Cre deletion (see Figures S1, S3, S6, and S7). For all crosses fl/+ and +/fl as superscripts mean that the offspring has inherited the floxed allele from the mother and father, respectively; Cre/+ and +/Cre as superscripts mean that the offspring has inherited the Cre recombinase from the mother and father, respectively; combination of fl/+, +/Cre means deletion of maternal floxed allele and combination of +/fl, Cre/+ means deletion of paternal floxed allele (see Table S5). Genotyping was performed by standard PCR using DNA extracted from ear biopsies (adult mice) or tail DNA (fetuses). PCR was performed using the Red Taq Ready PCR system (Sigma) (see list of primers in Table S6), followed by separation of PCR amplicons by agarose gel electrophoresis.

METHOD DETAILS

Plasma IGF2 measurements

IGF2 measurements were performed with the Mouse IGF-II DuoSet ELISA kit (R&D Systems – DY792), using an assay adapted for the MesoScale Discovery electrochemiluminescence immunoassay platform (MSD). Briefly, MSD standard-bind microtitre plates were first coated with 30μl capture antibody (Rat Anti-Mouse IGF-II, R&D Systems – 840962) diluted to 7.2 μg/ml in PBS, sealed, and incubated overnight at 4°C. After three washes with MSD wash (0.1% Tween 20 in PBS), the plates were loaded with 20μl ELISA Diluent RD5-38 per well, plus 10μl standard or plasma (diluted 50 fold in RIPA buffer, Sigma – R0278). The plates were then sealed and incubated for 2 h at room temperature on a plate shaker. After three washes with MSD wash, the wells were plated with 25μl detection antibody (Biotinylated Goat Anti-Mouse IGF-II, R&D Systems – 840963), diluted to 0.72 μg/ml in PBS, sealed, and incubated for 1 h at room temperature on a plate shaker. Following three additional washes with MSD wash, the wells were plated with 25μl MesoScale Discovery Streptavidin Sulpho-TAG (MSD – R32AD-1), diluted 1:1000 in the MSD Diluent 100, sealed and incubated for 30 min at room temperature on a plate shaker. After three final washes with MSD wash, the wells were plated with 150μl of MSD Read Buffer T (1x) and the reading was performed on the MSD s600 analyser. Each sample was measured in duplicate and the results were calculated against the standard curve, using the MSD Workbench Software.

Igf2 mRNA *in situ* hybridization

In situ hybridization was performed as described (Simmons et al., 2008), with minor modifications. Briefly, a region of 415bp spanning *Igf2* coding exons 4-6 was PCR amplified using primers: 5′-CACGCTTCAGTTTGTCTGTTCG-3′ and 5′-GCTGGACATCTCCGAAGAGG-3′ and E14 placental cDNA as template. The PCR amplicon was cloned into a pCR2.1-TOPO plasmid (Thermo Fisher Scientific – K450002). Sense (S) and antisense (AS) RNA probes were generated and labelled with Digoxigenin (DIG) by *in vitro* reverse transcription, according to manufacturer's instructions (Roche – 11175025910). E14 fetuses and placentae were collected in ice-cold PBS and fixed overnight in 4% paraformaldehyde in 0.1% diethylpyrocarbonate (DEPC)-PBS at 4°C. Tissues were then dehydrated and embedded in paraffin, using RNase-free conditions. Tissue sections (7μm thick) mounted on polysine slides were de-waxed, rehydrated in PBS, post-fixed in 4% paraformaldehyde for 10 min, digested with proteinase K (30μg/ml) for 10 min at room temperature, acetylated for 10 min (acetic anhydride, 0.25%) and hybridized overnight at 65°C in a humidified chamber with DIG-labeled probes diluted in hybridization buffer. Two 65°C post-hybridization washes (1 × SSC, 50% formamide, 0.1% tween-20) followed by two room temperature washes in 1 × MABT were followed by 30 min RNAse treatment. Sections were blocked for 1 h in 1 × MABT, 2% blocking reagent (Roche – 1096176001), 20% heat-inactivated goat serum and then incubated overnight with anti-DIG-AP antibody (Roche – 11093274910; 1:2,500 dilution) at 4°C. After 4x20 min washes in 1×MABT, slides were rinsed in 1 × NTMT and incubated with BCIP/NBT mix in NTMT buffer, according to manufacturer's instructions (Promega – S3771). Slides were counterstained with nuclear fast red, dehydrated, cleared in xylene and mounted in DPX mounting medium. Pictures were taken with an Olympus DP71 bright-field microscope fitted with a camera.

Western blot analysis

Tissues were lysed in ~10μl/mg tissue RIPA buffer (Sigma – R0278), then the lysates were spun at 3,000 RPM and 4°C for 15 min. The supernatants were transferred into new tubes and protein concentrations were quantified using the Pierce BCA Assay Protein kit (Thermo Fisher Scientific – 23225). 60μg total protein were mixed with SDS gel loading buffer, then denatured at 70°C for 10 min and loaded into 12-well NuPAGE Novex 4–12% Bis-Tris precast gels (Thermo Fisher Scientific – NP0322BOX). The pre-stained Novex Sharp protein standard (Invitrogen – LC5800) was used as protein marker. After electrophoresis for 40 min at 200V and 4°C, the proteins were transferred onto nitrocellulose membranes, using the iBlot Transfer Stacks (Invitrogen – IB3010-01) and the iBlot Gel Transfer Device set for 7 min at 20V. Blocking was performed for 1 h at 4°C in 5% semi-skimmed milk (Marvel) dissolved in TBS-T. The membranes were then incubated overnight at 4°C with the primary antibody dissolved in 0.5% milk in TBS-T (goat anti-human IGF2, 1:1,000, R&D Systems – AF292-NA or goat anti-mouse SOD1, 1:50,000, R&D Systems – AF3787). After 2 × 10 min washes with milliQ water and 2 × 10 min washes with TBS-T, the blots were incubated for 1 h at room temperature with the secondary antibody dissolved in TBS-T containing 3% semi-skimmed milk (rabbit anti-goat IgG-HRP, 1:2,500, Santa Cruz sc-2768). The blots were then washed as above, exposed to substrate (Clarity ECL Western Blotting Substrate, Bio-Rad – 1705060) for 5 min and imaged with the Bio-Rad GelDoc system. Stripping of antibodies was carried out using a stripping buffer (Thermo Fisher Scientific – 21059) for 15 min at room temperature. The band intensities were quantified using the ImageLab software (Bio-Rad) and expressed as IGF2/SOD1 ratios.

Placenta stereology

Placenta stereology analyses for the $Igf2^{EpiKO}$ and $Igf2^{ECKO}$ models were performed as described (Coan et al., 2004) in placentae (n=5–7) collected from three litters at each developmental stage. Briefly, the placentae were weighted, then halved, and each half placenta was weighted again. A half was fixed in 4% paraformaldehyde in PBS at 4°C overnight, then dehydrated and embedded in paraffin wax. The paraffin blocks were exhaustively sectioned using a microtome at 7μm thickness. Placental sections spaced 140 μm apart were hematoxylin-eosin stained and stereological measurements of placental layers were done using the NewCAST system (Visiopharm, Hoersholm, Denmark), using the point counting method (Coan et al., 2004).

The corresponding placental halves were fixed for 6 h with 4% glutaraldehyde in 0.1 M PIPES buffer, washed with 0.1 M PIPES buffer, and treated with 1% osmium tetroxide. The samples were then resin-embedded and 1μm thick sections, obtained close to the placental midline, were stained with methylene blue. Analysis of Lz components was done using the NewCAST system (Visiopharm) with meander sampling of ~25% of the Lz area.

Placental stereology for $Igf2^{HCKO}$ model was performed as described (De Clercq et al., 2020). Briefly, placental samples were embedded in paraffin as described above, sectioned, and then double-labelled for lectin and cytokeratin, which allows the identification of Lz constituents. The proportion of FC, MBS and LT was quantified using the NewCAST system and the point counting method, as described above.

Transmission electron microscopy

Analysis of E16 $Igf2^{EpiKO}$ mutant and control placentae by transmission electron microscopy was performed as previously described (Coan et al., 2005). Briefly, resin-embedded 1 μm thick sections, cut near placental midline and stained with methylene blue as described in the previous section (placenta stereology) were used to identify regions of interest. Thin sections (50 nm) were stained with uranyl acetate and lead citrate, and viewed using a Philips CM100 transmission electron microscope at 80 kV.

Immunostainings

Immunohistochemistry or immunofluorescence conditions are listed in Table S7. TUNEL staining was performed using the In Situ Cell Death Detection Kit, TMR red (Sigma – 012156792910), or the TUNEL Assay Kit – BrdU-Red (Abcam – ab66110) according

to manufacturer's protocols. EdU staining was done with the Click-iT EdU Alexa Fluor 488 Imaging Kit (Invitrogen – C10337), according to manufacturer's instructions. For all immunofluorescence stains, DAPI (Sigma – D9542) was used to label the nuclei. For all immunohistochemistry, images were taken with an Olympus DP71 bright-field microscope. Immunofluorescence image acquisition was performed using a LSM510 Meta confocal laser scanning microscope (Carl Zeiss, Jena, Germany) and the ZEN 2009 software or a SP8 laser-scanning confocal microscope (Leica, Mannheim, Germany). Fluorescence semi-quantification analysis was performed using Volocity 6.3 (Improvision). Counting of TUNEL$^+$ and F4/80$^+$ cells was performed using HALO image analysis software (PerkinElmer).

qRT-PCR analysis

Total RNA was extracted using RNeasy Plus Kits (Qiagen – 74134 and 74034). RNA concentration was measured by NanoDrop (Thermo Fisher Scientific) and quality was assessed in agarose gels. RNA extracted from FACS isolated cells was quantified and assessed for quality using the RNA 6000 Pico Kit (Agilent – 5067-1513) and an Agilent 2100 Bioanalyzer. Reverse transcription was performed using the RevertAid RT Reverse Transcription Kit (Thermo Fisher Scientific – K1622). qRT-PCR was performed with the SYBR Green JumpStart Taq Ready Mix (Sigma – S4438) and custom-made primers (Table S6) using an ABI Prism 7900 system (Applied Biosystems). For gene expression normalization, we used four housekeeping genes (*Gapdh*, *Sdha*, *Pmm1*, *Ppia*). Levels of expression were calculated using the $2^{-\Delta\Delta Ct}$ method (Livak and Schmittgen, 2001).

Expression microarray analysis

Total RNA was extracted from E19 male Lz using RNeasy Midi Kits (Qiagen – 75144) and quantity and quality were verified using RNA 6000 Nano Kit (Agilent – 5067-1511) and an Agilent 2100 Bioanalyzer. Only RNA samples with RNA integrity numbers (RIN) >9.0 were used. Array profiling was performed using the Mouse Gene 1.0 ST Array (Affymetrix – 901171) and the analysis of the data were performed using GeneSpring GX 12.1 (Agilent, Santa Clara, CA, USA), with two algorithms: RMA (Robust Multiarray Average) and PLIER (Probe Logarithmic Intensity Error). Only genes with $\log_2$ fold change >0.3 predicted by both algorithms were listed as DEGs. Pathway analysis was performed using Ingenuity Pathway Analysis (version 2012).

Flow cytometry analyses

For flow cytometry analyses of FPEC, Lz samples were micro-dissected in ice-cold PBS. Tissue dissociation into single cells was achieved by digestion at 37°C for 45 min with a 0.1% collagenase P solution, aided by mechanical dissociation with needles of decreasing diameter. The cells were then passed through 70-μm cell strainers and washed once in ice-cold PBS + 0.1% BSA. Erythrocytes were lysed using the RBC lysis buffer (BioLegend – 420301). Pelleted cells were then re-suspended in 100μl staining buffer (BioLegend – 420201), counted using the Cedex XS Analyser (Roche) and diluted at 1,000 cells/μl. Blocking of Fc receptors was performed by incubation at 4°C for 20 min with an unlabelled anti-CD16/32 (1 μg/million cells; BioLegend – 101320). The cells were then incubated for 1 h at 4°C in the dark with a mix of rat anti-mouse CD41 (labelled with Phycoerythrin, PE) (BioLegend – 133906; 0.25 μg per million cells), rat anti-mouse CD31 (labelled with AF647) (BioLegend – 102516; 0.25 μg per million cells) and rat anti-mouse CD45 (labelled with V500) (BD Horizon – 561487; 0.4 μg per million cells) in 200μl staining buffer. Stained cells were washed twice in 1ml staining buffer, re-suspended in PBS containing a viability marker (7-AAD – 7-Aminoactinomycin, Invitrogen – A1310), filtered again through 70-μm cell strainers and incubated on ice for 5 min. Flow cytometry analysis was performed with a BD FACSCantoII machine (BD Biosciences) and 100,000 events were recorded for each sample. FSC files were analysed with the FlowJo_V10 software, using single-cell discrimination and gating based on single-stained controls. FPEC were identified as 7AAD$^-$/CD31$^+$/CD41$^-$ cells.

For flow cytometry analyses of EPCAM^{high} positive cells, whole E12 placentae were dissociated into single cells as described above. After erythrocyte lysis, cell counting, and blocking of Fc receptors using an unlabelled anti-CD16/32 antibody, cells were incubated for 1 h at 4°C in the dark with AF647 rat anti-mouse CD326 (Epcam) antibody (BioLegend – 118212; 0.25 μg per million cells) in 200μl staining buffer. Stained cells were washed as above, incubated with the viability marker 7-AAD and filtered through 70-μm cell strainers. Flow cytometry analysis was performed with a BD FACSCantoII machine (BD Biosciences) and FSC files were analysed with the FlowJo_V10 software, using single-cell discrimination and gating based on single-stained controls.

Flow cytometry analysis of FPEC proliferation

Pregnant female mice received intraperitoneal (i.p.) injections with 50μg of 5-ethynyl-2′-deoxyuridine (EdU)/g body weight (Thermo Fisher Scientific – A10044), 16 h prior to tissue collection. Lz dissociation into single cells was performed as above. Cells re-suspended at a concentration of 1000 cells/μl were incubated for 30 min at 4°C with 1 μl Red LIVE/DEAD Fixable Dead Cell Stain (Thermo Fisher Scientific – L23102). After one wash in PBS, the cells were pre-incubated for 20 min at 4°C in the dark with unlabelled rat anti-mouse CD16/32 (BioLegend – 101320, 1 μg/million cells), then for 1 h at 4°C in the dark with a 1:1 mix of rat anti-mouse CD41 (labelled with BV421) (BioLegend – 133911; 0.25 μg per million cells) and rat anti-mouse CD31 (labelled with AF647) (BioLegend – 102516; 0.25 μg per million cells) in staining buffer. After two washes with staining buffer, the cells were stained using the Click-iT EdU Alexa Fluor 488 Flow Cytometry Assay Kit (Thermo Fisher Scientific – C10420), according to manufacturer's instructions. Flow cytometry analysis was performed using a BD LSRFortessa cell analyser (BD Biosciences). FSC files were analysed with the FlowJo_V10 software, using single-cell discrimination and gating based on single-stained controls. Proliferating FPEC were identified as viable EdU$^+$/CD31$^+$/CD41$^-$ cells.

FPEC and HC isolation by FACS

For sorting FPEC, single cell preparation and staining was performed as above. For sorting HC, entire placentae collected at E13 were used for single cell preparation as described above. Single cell suspensions were pre-incubated for 20 min at 4°C in the dark with unlabelled rat anti-mouse CD16/32 (BioLegend – 101320, 1 μg/million cells), then stained for 1 h at 4°C in the dark with a mix of rat anti-mouse CD117/c-kit (labelled with PE) (BioLegend – 105808; 0.4 μg per million cells), rat anti-mouse CD34 (labelled with AF700) (ThermoFisher Scientific – 56-0341-82; 1 μg per million cells), rat anti-mouse Ly-6A/E (Sca1) (labelled with BV510) (BioLegend – 108129; 5 μl per million cells) and rat anti-lineage cocktail (labelled with BV421) (BioLegend – 133311; 5 μl per million cells) in staining buffer and washed twice. FACS was done using an Aria-Fusion cell sorter (BD Bioscience), with exclusion of cell duplets and dying cells ($7AAD^+$). Cell fractions (endothelial, non-endothelial and hematopoietic cells) were then spun at 3,000 RPM and 4°C for 3 min, the excess of sorting liquid was removed and cell pellets were flash frozen in liquid nitrogen and stored at -80°C until used for RNA extraction.

Primary FPEC isolation, culture, and tube formation assay

Primary FPEC were isolated as previously described (Branco-Price et al., 2012) and adapted here to Lz (E16). Briefly, Lz were micro-dissected on ice in RPMI containing 1% penicillin/streptomycin. All samples from one litter were pooled, minced, and digested for 90 min at 37°C in 2 mg/ml collagenase type I (Sigma – SCR103) in HBSS containing 2mM CaCl2, 2mM MgSO4, and 20mM HEPES. The digests were filtered through 70μm nylon cell strainers and washed in HBSS. The cell pellets were then resuspended in PBS containing 0.1% BSA and incubated with anti-CD31-coated magnetic beads for 1 h at 4°C. Cells coated with beads were cultured in endothelial cell growth medium consisting of low glucose DMEM:F12 with 1% nonessential amino acids, 2mM sodium pyruvate, buffered with 20mM HEPES and supplemented with 20% FBS and 75μg/ml endothelial mitogens (Sigma – E2759). The cells were incubated at 37°C in 5% O2 and 5% CO_2. After four days, the dead cells were washed and new media was added, additionally supplemented with 20μg/ml Heparin. Sub-confluent cells (~80%) at passage one (around 10 days in culture) were washed and then cultured in 5% serum replacement media (Sigma – S0638) for ~40 h. From each litter we used cells at passage one for treatment with 50 ng/ml mouse recombinant IGF2 (R&D Systems, 792-MG-050; dissolved in PBS), 1000 ng/ml human $IGF2^{Leu27}$ (GroPep – TU100; dissolved in 10mM HCl), 500nM picropodophyllotoxin (PPP, Sigma – T9576; dissolved in DMSO) or 500nM PPP + 50 ng/ml IGF2, or appropriate vehicle control. The cells were harvested with Accutase (Sigma – A6964) and counted using the ADAM™ Automated cell counter (NanoEnTek Inc) and 3,000 cells were seeded into 15-well Angiogenesis μ-Slides (Ibidi – 81506) preloaded with 10μl matrigel/well (BD Biosciences – 354234). Photographs were taken at 30 min, 4, 6 and 8 h using an EVOS FL Cell Imaging system (Thermo Fisher Scientific). Each experiment was performed on 5–6 litters for every treatment. For each tube formation assay, we used five wells seeded with primary FPEC exposed to the treatment agent with equivalent numbers of the corresponding vehicle. Quantification of tubular network structures was performed using the Angiogenesis Analyzer software in ImageJ (Schneider et al., 2012).

siRNA knockdown of *Igf2r*

Small interfering RNA (siRNA) knockdown of *Igf2r* was performed on primary placental microvascular endothelial cells isolated from C57BL/6J mice (Cell Biologics, C57-6056) and grown in Cell Biologics' complete growth medium (M1168) under standard culture conditions (37°C in 21% O2 and 5% CO_2). Endothelial cells were transfected with stealth siRNA for *Igf2r* or scrambled siRNA (Thermo Fisher Scientific 1320003 and 12935100, respectively) using Lipofectamine RNAiMax transfection reagent (Thermo Fisher Scientific 13778075).

The impact of *Igf2r* knockdown on endothelial cell proliferation rates, with or without exogenous IGF2 stimulation, was analysed using a previously described protocol (Woods et al., 2017). In brief, 10,000 endothelial cells transfected with either scrambled or *Igf2r* siRNA were plated in basal medium (M1168b, which does not contain VEGF, ECGS, EGF and FBS) supplemented with hydrocortisone, heparin, and serum replacement (Sigma, S0638) in the presence or absence of 50 ng/ml recombinant mouse IGF2 (R&D Systems, 792-MG-050), and collected every 24 h over a period of four days. The number of viable cells was counted using the Countess 3 Automated Cell Counter (Thermo Fisher Scientific), according to manufacturer's instructions.

To study the impact of *Igf2r* knockdown on intracellular signalling pathways, following 48h of transfection, cells were starved in the basal medium (M1168b) for 20h and then stimulated with recombinant mouse IGF2 (50 ng/ml) and collected at the specific times (1, 5 and 10 min). Total cell extracts were prepared in radioimmunoprecipitation assay buffer (20 mM Tris-HCl, pH 8.0, 137 mM NaCl, 1 mM MgCl2, 1 mM CaCl2, 10% glycerol, 1% NP-40, 0.5% sodium deoxycholate, 0.1% sodium dodecyl sulphate), containing a protease inhibitor cocktail (Sigma, P2714), and incubated at 4°C for 1 h. Western blotting was performed as previously described (Pérez-García et al., 2014). Blots were probed with the following antibodies: rabbit anti IGF2R (Cell Signaling, 14364), mouse anti-Phospho-p44/42 MAPK (Erk1/2) (Thr202/Tyr204) (Cell Signaling, 9106), rabbit anti-p44/42 MAPK (Erk1/2) (Cell Signaling, 9102), rabbit anti-Phospho-Akt (Ser473) (Cell Signaling, 9271), rabbit anti-Akt (Cell Signaling, 9272), rabbit anti-HSP90 (Cell Signaling, 4877). Horseradish peroxidase-conjugated secondary antibodies were from Bio-Rad. Detection was carried out with enhanced chemiluminescence reaction (GE Healthcare, RPN2209) using standard X-ray films. Band intensities were quantified using ImageJ software.

RNA-sequencing and data analysis

Total RNA was extracted from sorted FPEC by FACS from E16 male placentae using RNeasy Plus Micro Kits (Qiagen – 74034). Quantity and quality were verified using the RNA 6000 Pico Kit (Agilent – 5067-1513) and an Agilent 2100 Bioanalyzer. Only RNA samples

with RNA integrity numbers (RIN) >9.0 were used. Total RNA (2 ng) was whole-transcriptome amplified using the Ovation RNA–Seq System V2 (NuGEN). To prepare the RNA–seq libraries the amplified cDNA (2μg per sample) was fragmented to 200bp using a Bioruptor Sonicator (Diagenode), end repaired and barcoded using the Ovation Rapid DR Library System (NuGEN). The libraries were combined and loaded onto an Illumina HiSeq 2500 system for single-end 50bp sequencing at the Genomics Core Facility, Cambridge Institute, CRUK. The reads were aligned onto the mouse GRCm38 genome using TopHat 2.0.11 (Kim et al., 2013). Gene abundance and differential expression were determined with Cufflinks 2.2.1 (Trapnell et al., 2010) and expressed in fragments per kilobase per million mapped reads (FPKM). The cut off for expression was set at $\geq$1 FPKM. Genes with a linear fold expression change greater than 1.5 and a Benjamini–Hochberg false discovery rate (FDR) <5% were considered differentially expressed.

Functional analysis was performed using DAVID (Database for Annotation, Visualization and Integrated Discovery; v6.8 https://david.ncifcrf.gov). Enriched gene ontology (GO) terms with FDR < 5% were considered significant. These terms were then clustered semantically using REViGO (Reduce and Visualize GO) (Supek et al., 2011), which removes redundancy, and ordered according to the $\log_{10}$ p values.

To search for enrichment of TF binding sites at the promoters of DEG, we used EPD (Eukaryotic Promoter Database – https://epd.vital-it.ch/index.php) to retrieve the DNA sequences from 1,000bp upstream to 100bp downstream of the transcriptional start site (TSS). These sequences were then analysed using AME (Analysis of Motif Enrichment v4.12.0 – http://meme-suite.org/tools/ame) by selecting *Mus musculus* and HOCOMOCO Mouse (v11 FULL) as motif database. Transcriptional network visualization was performed using the Ingenuity Pathway Analysis tool.

QUANTIFICATION AND STATISTICAL ANALYSIS

No statistical analysis was used to predetermine sample size. Randomization was not used in our animal studies. Placental stereology and histological EdU analyses were performed blinded to genotype. Statistical analyses for fetus, placenta and Lz growth kinetics were performed in R, using a mixed effects model, with litter as a random effect and genotype, developmental stage, and the interaction between genotype and developmental stage as fixed effects. Prior to these analyses, fetal, placental and Lz weights were log transformed. All other statistical analyses were performed using GraphPad Prism 8. Statistical significance between two groups was determined by Mann-Whitney tests or two-tailed unpaired t tests and statistical significance between multiple groups was performed using one-way ANOVA plus Tukey's multiple comparisons tests or two-way ANOVA plus Sidak's multiple comparisons tests, as appropriate. The numbers of samples or litters used for each experiment are indicated in figure legends.

Developmental Cell, Volume *57*

Supplemental information

The imprinted *Igf2-Igf2r* axis is critical for matching placental microvasculature expansion to fetal growth

Ionel Sandovici, Aikaterini Georgopoulou, Vicente Pérez-García, Antonia Hufnagel, Jorge López-Tello, Brian Y.H. Lam, Samira N. Schiefer, Chelsea Gaudreau, Fátima Santos, Katharina Hoelle, Giles S.H. Yeo, Keith Burling, Moritz Reiterer, Abigail L. Fowden, Graham J. Burton, Cristina M. Branco, Amanda N. Sferruzzi-Perri, and Miguel Constância

SUPPLEMENTAL INFORMATION

FIGURE S1

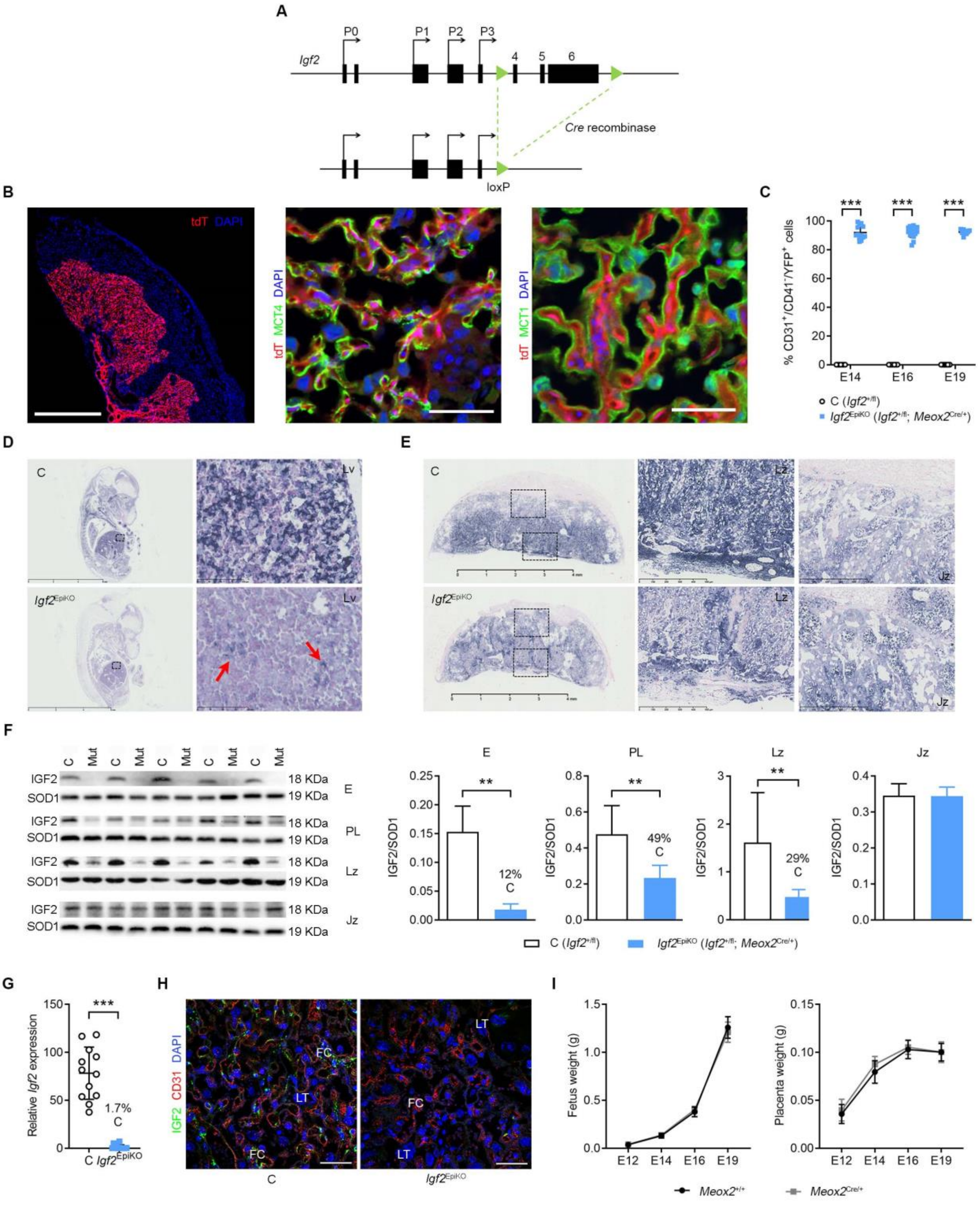

Figure S1. Specificity and efficiency of *Igf2* deletion in fetal tissues and feto-placental endothelial cells by *Meox2*^{Cre}. Related to Figure 2.

(**A**) Schematic representation of the floxed *Igf2* allele. P0-P3 are alternative promoters. Protein-coding exons (4–5), flanked by loxP sites (green triangles), are excised upon Cre-loxP mediated recombination. (**B**) Representative confocal microscopy of a placental frozen section at E16 of

gestation, double transgenic for *Meox2*^{Cre} and Ai9(RCL-tdT) reporter. The *Meox2*^{Cre} is not expressed in the syncytiotrophoblast layers, as demonstrated by the lack of immunostaining overlap between the tomato protein (red) and MCT4 (a marker of the syncytiotrophoblast layer II, facing the feto-placental capillaries) or MCT1 (marker of the syncytiotrophoblast layer I, facing the maternal blood spaces). Scale bars are 1 mm (left panel) and 50 µm (middle and right panels). (**C**) Flow cytometry analysis shows that the majority (>80%) of *Igf2*^{EpiKO} mutant FPEC (CD31^{+}/CD41^{-} cells) express YFP (activated by *Meox2*^{Cre} mediated deletion of the *Rosa26* ^{fl}STOP^{fl}YFP STOP cassette), thus demonstrating good efficiency of *Meox2*-Cre in these cells (n=9–18 per genotype). (**D**) *Igf2* mRNA in situ hybridization (ISH) in E14 control and mutant fetuses. Dark blue indicates *Igf2* mRNA, with nuclei marked in red. Insets illustrate efficient *Igf2* deletion in the liver (Lv); arrows – small pockets of cells with incomplete *Igf2* deletion (mosaic activity of *Meox2*^{Cre}). Scale bars are 6 mm (left) and 100 µm (right). (**E**) *Igf2* mRNA ISH in E14 control and mutant placentae. Insets show reduced *Igf2* mRNA signal in the placental labyrinthine zone (Lz) of mutants, due to its deletion from FPEC, while *Igf2* expression is unchanged in the junctional zone (Jz). Scale bars are 4 mm (left) and 500 µm (right). (**F**) Western blot analysis of pro-IGF2 (18 kDa) in cell lysates from whole fetuses (F), whole placenta (PL) micro-dissected Lz and Jz at E14, and corresponding data quantification shown as graphs (n=5 per genotype). SOD1 (19 kDa) was used as loading control. (**G**) Efficiency of *Igf2* deletion evaluated by qRT-PCR in fluorescence-activated sorted FPEC (n=12 per genotype). (**H**) Representative immunofluorescence confocal microscopy at E16 showing near complete absence of IGF2 protein within CD31+ feto-placental endothelial cells in *Igf2*^{EpiKO} mutants compared to littermate controls. FC – fetal capillaries, LT – labyrinthine trophoblast. Scale bars are 50µm. (**I**) Fetal and placental growth kinetics are not altered in *Meox2*$^{Cre/+}$ carriers (maternal inheritance) (n=8–30 conceptuses per genotype at each developmental stage). For all graphs, data is shown as individual values or averages ± SD; ** $P < 0.01$, *** $P < 0.001$ calculated by two-way ANOVA plus Sidak's multiple comparisons tests in (C) or Mann Whitney tests in (F) and (G).

Figure S2

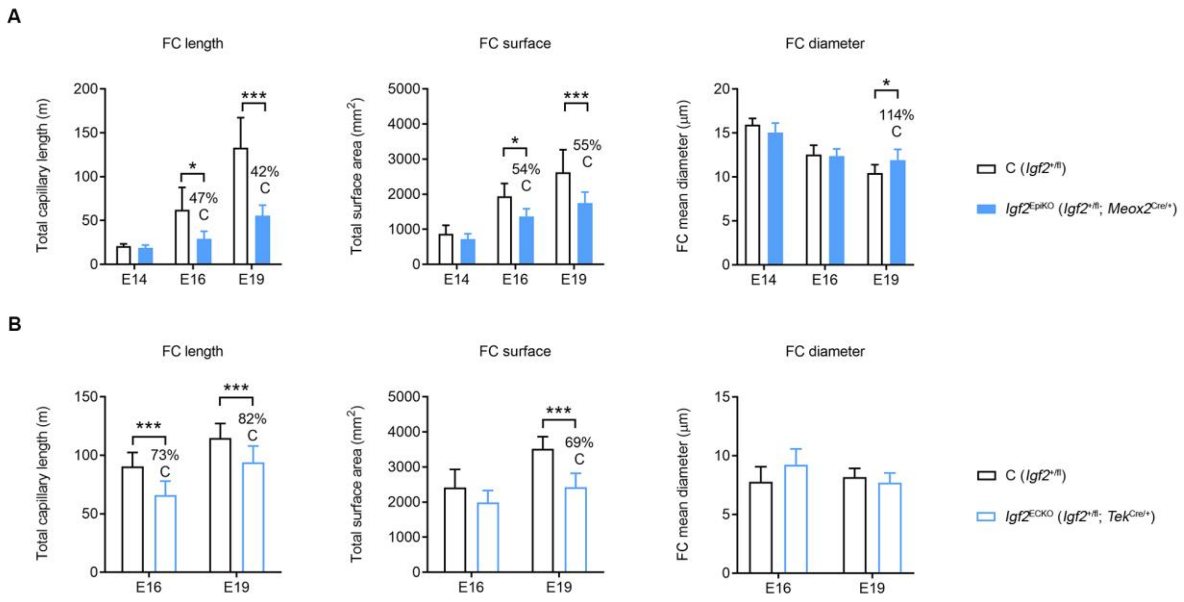

Figure S2. Impact of *Igf2*^{EpiKO} and *Igf2*^{ECKO} deletions on feto-placental capillary expansion during gestation. Related to Figure 2.

(**A**) Parameters of feto-placental capillaries (FC) measured by stereology in *Igf2*^{EpiKO} mutant (*Igf2*$^{+/fl}$; *Meox2*$^{Cre/+}$) versus control (C – *Igf2* $^{+/fl}$) placentae (n=6 per genotype at each developmental stage). (**B**) Parameters of feto-placental capillaries (FC) measured by stereology in *Igf2*^{ECKO} mutant (*Igf2*$^{+/fl}$; *Tek*$^{Cre/+}$) versus control (C – *Igf2* $^{+/fl}$) placentae (n=5–7 per genotype at each developmental stage). For all graphs, data is shown as averages ± SD; * $P < 0.05$, *** $P < 0.001$ calculated by two-way ANOVA plus Sidak's multiple comparisons tests.

Figure S3

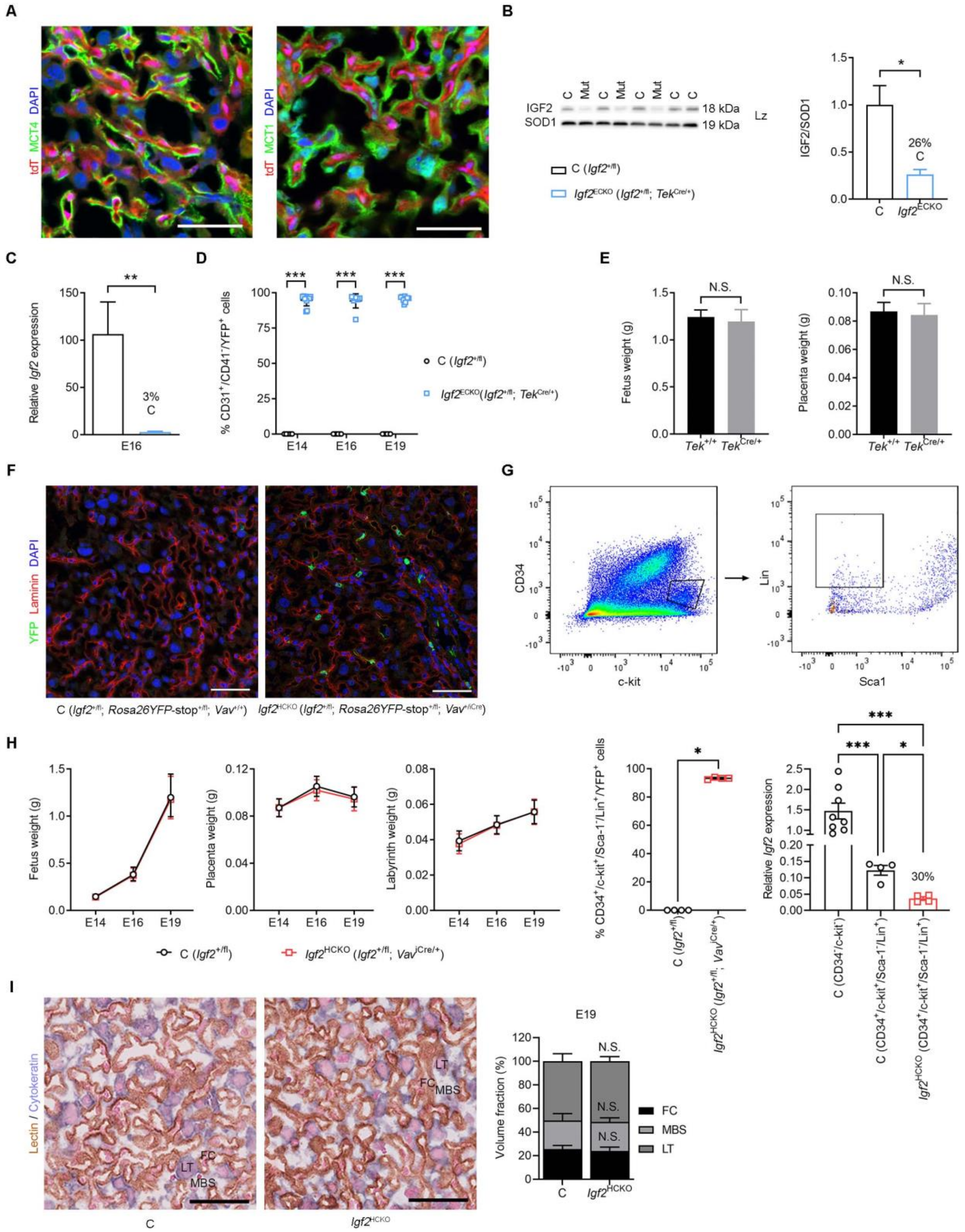

Figure S3. Specificity and efficiency of *Igf2* deletion in the endothelium by Tek^{Cre} and in the hematopoietic lineage by Vav^{iCre}. Related to Figure 2.

(**A**) Representative confocal microscopy of frozen placental sections from a double transgenic for TeK^{Cre} and Ai9(RCL-tdT) reporter at E16 of gestation. The TeK^{Cre} is not expressed in the syncytiotrophoblast layers, as demonstrated by the lack of immunostaining overlap between the

tomato protein (red) and MCT4 (Syn-TII layer) or MCT1 (Syn-TI layer). Scale bars are 50 µm. (**B**) Western blot analysis of pro-IGF2 (18 kDa) in cell lysates from Lz micro-dissected at E16 and corresponding data quantification (n=3 per genotype). SOD1 (19 kDa) was used as internal control for loading. (**C**) Efficiency of *Igf2* deletion evaluated by qRT-PCR in fluorescence-activated sorted FPEC (n=5–7 per genotype). (**D**) Flow cytometry analysis shows that the majority (>80%) of *Igf2*^{ECKO} mutant FPEC express YFP, thus demonstrating good efficiency of *TeK*^{Cre} in these cells (n=5–11 per genotype). (**E**) Fetal and placental growth kinetics are not altered in *TeK*$^{Cre/+}$ carriers (maternal inheritance) at E19 (n=13–15 conceptuses per genotype from 4 independent litters). (**F**) Representative double immunostainings for laminin (marking feto-placental capillaries) and YFP in E19 *Igf2*^{HCKO} mutant and littermate control (C) placentae. YFP expression is activated in cells of hematopoietic lineage by deletion of the floxed STOP cassette within the *Rosa26*^{fl}STOP^{fl}YFP reporter construct. Scale bars are 50µm. (**G**) Gating strategy used to isolate cells of hematopoietic lineage from E13 placentae by FACS (top) and efficiency of *Igf2* deletion evaluated by qRT-PCR (bottom). The CD34^{+}/c-kit^{+} population gated in the top left panel was subsequently used to isolate Lin^{+}/Sca1^{-} hematopoietic cells (top right panel). Bottom left: more than 90% of CD34^{+}/c-kit^{+}/Lin^{+}/Sca1^{-} cells are positive for YFP, indicating efficient activity of *Vav*^{iCre} in the hematopoietic cells. Bottom right: measurement of mRNA levels at E13 by qRT-PCR indicates overall low levels of *Igf2* expression in the hematopoietic lineage (approximately one order of magnitude lower than in CD34^{-}/c-kit^{-} cells), as well as efficient deletion of *Igf2* in hematopoietic cells of *Igf2*^{HCKO} mutants compared to littermate controls (n=4–8/group). (**H**) Fetal, placental, and Lz growth kinetics are not altered in *Igf2*^{HCKO} mutants compared to controls (n=11–25 conceptuses from n=5–6 litters for each developmental stage). (**I**) Left: representative double immunostaining for lectin (brown, marking feto-placental capillaries) and cytokeratin (blue, marking the labyrinthine trophoblast) in E19 placental sections of *Igf2*^{HCKO} mutants and littermate controls. Right: the relative volume fractions occupied by the three main labyrinthine constituents (FC – fetal capillaries, MBS – maternal blood spaces and LT – labyrinthine trophoblast) are not altered in *Igf2*^{HCKO} mutants compared to controls at E19 (n=4–7 samples/group). For all graphs, data is shown as individual values or averages ± SD in (B), (D), (E), (G) – bottom left and (I), SEM in (C) and (G) – bottom right, or 95% confidence intervals (95%CI) in (H); N.S. – statistically non-significant; * $P < 0.05$, ** $P < 0.01$ and *** $P < 0.001$ calculated by Mann Whitney tests in (B), (C), (E) and (G) – bottom left, two-way ANOVA plus Sidak's multiple comparisons tests in (D) and (I), Brown-Forsythe and Welch ANOVA tests in (G) – bottom right and mixed effects model in (H).

Figure S4

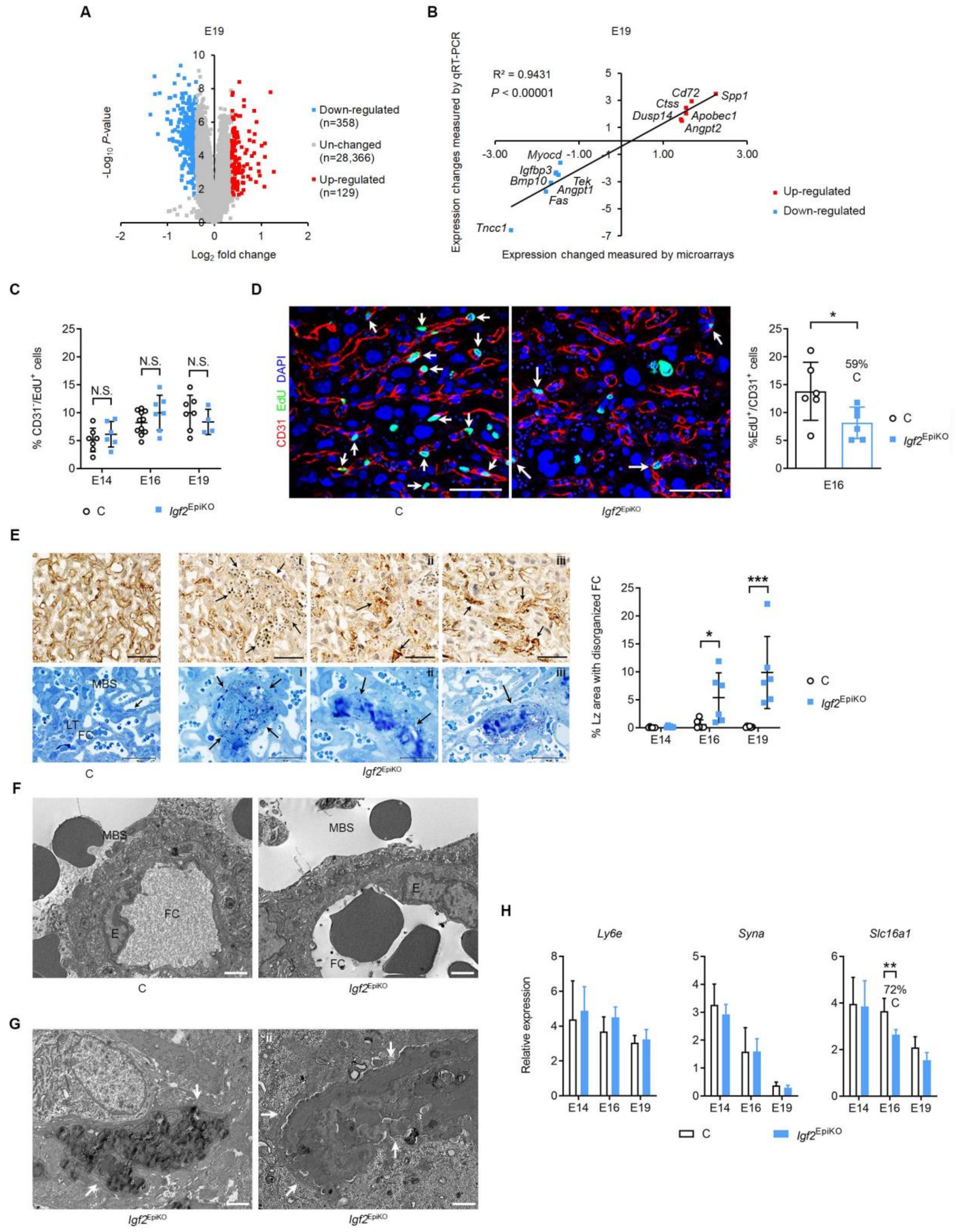

Figure S4. Fetus-derived *Igf2* deletion (*Igf2*^{EpiKO}) alters gene expression in placental Lz and FPEC survival and proliferation. Related to Figure 3.

(**A**) Volcano plot depicting differentially expressed genes (DEG) identified in E19 Lz by expression microarray analysis (n=6 samples per genotype, all from male conceptuses). (**B**) Biological validation using qRT-PCR for 13 DEGs (n=6–7 samples per genotype), normalized against three housekeeping

genes (*Sdha*, *Gapdh* and *Pmm1*). (**C**) The reduction in proliferation seen in FEPC (Fig. 3e) is not observed in non-endothelial cells from Lz measured by flow cytometry analysis after EdU injections (16 hours exposure; n=4–11 per group). (**D**) Representative confocal microscopy image of FPEC proliferation in control (panel i) versus mutant (panel ii) E16 placentae by immunofluorescent staining for CD31 combined with Click-iT EdU imaging. Arrows point towards FPEC nuclei that incorporated EdU *in vivo* during the 16 hours exposure to the thymidine analogue. Scale bar is 50 µm. The accompanying graph shows data quantification based on counting between 250 to 600 FPEC per sample (n=6 placentae/genotype). **(E)** Top row: CD31 staining in control (C) and *Igf2*^{EpiKO} mutant E16 Lz illustrating abnormally large FCs lacking endothelial cells (i) or obstructed capillaries surrounded by fragmented and disorganized FPEC (ii, iii). Scale bars are 50µm. Bottom row: methylene blue-stained E16 Lz resin sections (arrows indicate a FPEC in C and thrombotic FC in mutants: i-iii). Scale bars are 30µm. The graph on the right side shows the quantification of percentage of Lz areas with disorganized pattern of FC. Measurement of total Lz and abnormal Lz areas was performed using HALO image analysis software (n=6 samples per genotype at each developmental stage). (**F**) Representative transmission electron microscopy (TEM) micrographs showing intact feto-maternal barriers separating fetal capillaries (FC) from maternal blood spaces (MBS) in both controls (C) and *Igf2*^{EpiKO} mutants, at E16. Scale bars are 2µm (n=3 biological replicates per group with 6-15 micrographs/sample). (**G**) TEM micrographs from E16 *Igf2*^{EpiKO} mutants. White arrows point towards an apoptotic endothelial cell (panel i) and a blocked/thrombotic capillary with apoptotic endothelial lining (panel ii). In both cases the overlying trophoblast is intact. Scale bars are 2µm. (**H**) qRT-PCR analysis of genes expressed in Syn-TI in micro-dissected Lz in mutants versus controls (n=6–8 samples per group for each developmental time point). For all graphs, data is presented as averages or individual values ± SD; N.S. – non-significant, * $P < 0.05$, ** $P < 0.01$, *** $P < 0.001$ by two-way ANOVA plus Sidak's multiple comparisons tests in (C), (D) and (H) or Mann Whitney test in (E).

Figure S5

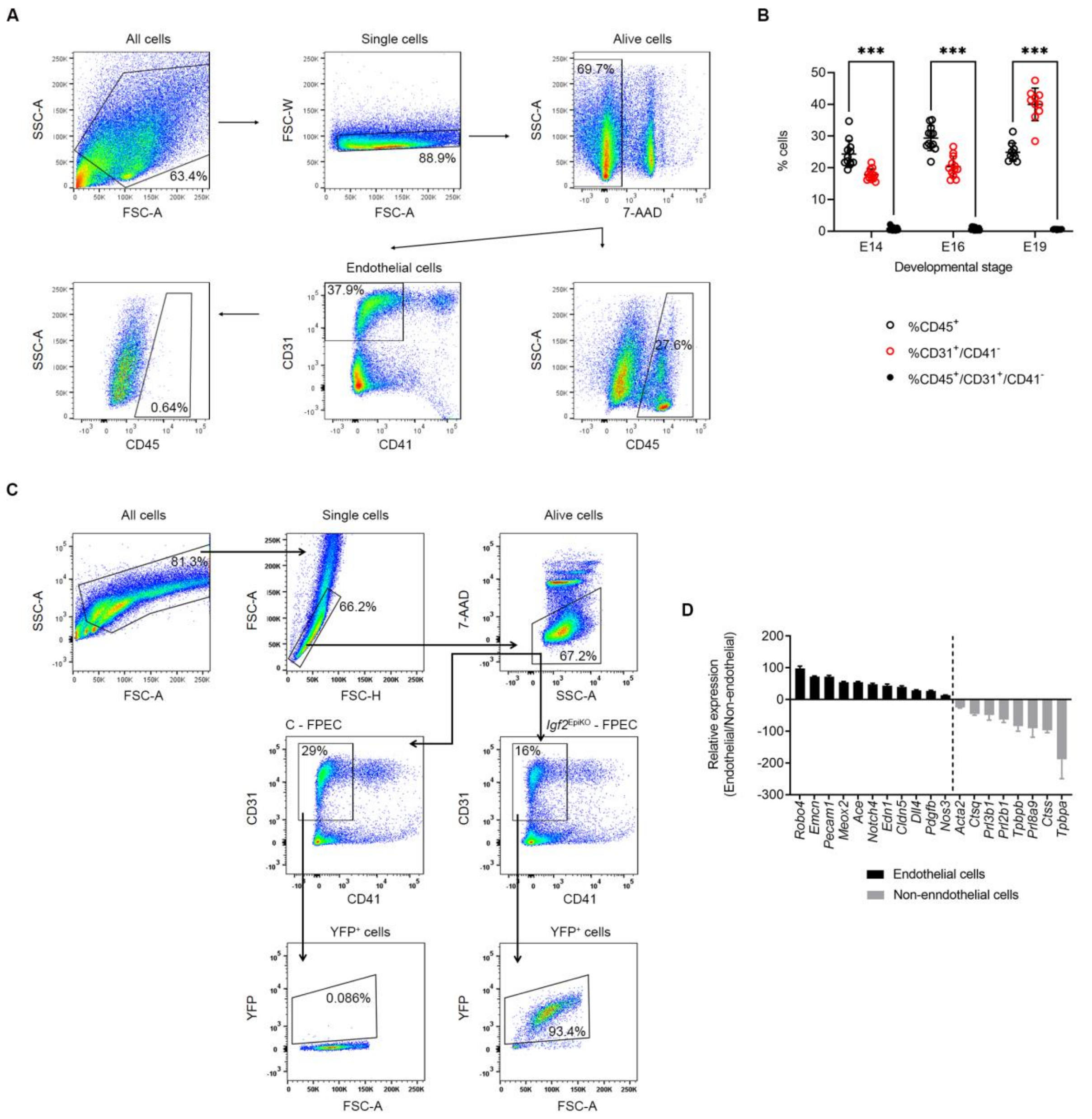

Figure S5. Experimental design used for FPEC analysis by flow cytometry and FPEC isolation by FACS. Related to Figures 4 and 5.

(**A**) Gating strategy used for flow cytometry analysis of CD45 expression (marker of all differentiated hematopoietic cells, except erythrocytes and plasma cells) within FPEC (defined as $CD31^+/CD41^-$ cells). The example shown is from an E19 sample. (**B**) Statistical analysis of CD45+, $CD31^+/CD41^-$ (FPEC) and $CD45^+/CD31^+/CD41^-$ cells at E14, E16 and E19. At all three developmental stages, the proportion of $CD45^+$ within the FPEC ($CD31^+/CD41^-$) is very low. The graph show individual data points with averages and SD (n=10-12 samples from two litters at each developmental stage). *** $p < 0.001$ by two-way ANOVA with Tuckey's multiple comparisons test. (**C**) Gating strategy used for FPEC and YFP analysis by flow cytometry and FPEC isolation by FACS. Mutant FPEC are also positive for YFP (activated by Cre mediated deletion of the *Rosa26* ^{fl}STOP^{fl}YFP STOP cassette), thus providing an internal control for Cre efficiency in each biological sample. (**D**) RNA-seq analysis of marker genes expressed in FPEC or non-endothelial cells isolated by FACS from E16 control Lz. The graph shows the relative enrichment in

FPEC of known markers of endothelial cells (black) and depletion of marker genes expressed by other cell types found in the Lz (grey): pericytes (*Acta2*), sinusoidal trophoblast giant cells (*Ctsq*, *Prl2b1*, *Prl8a9*), parietal trophoblast giant cells (*Prl3b1*) or spongiotrophoblast cells (*Tpbpa*, *Tpbpb*).

Figure S6

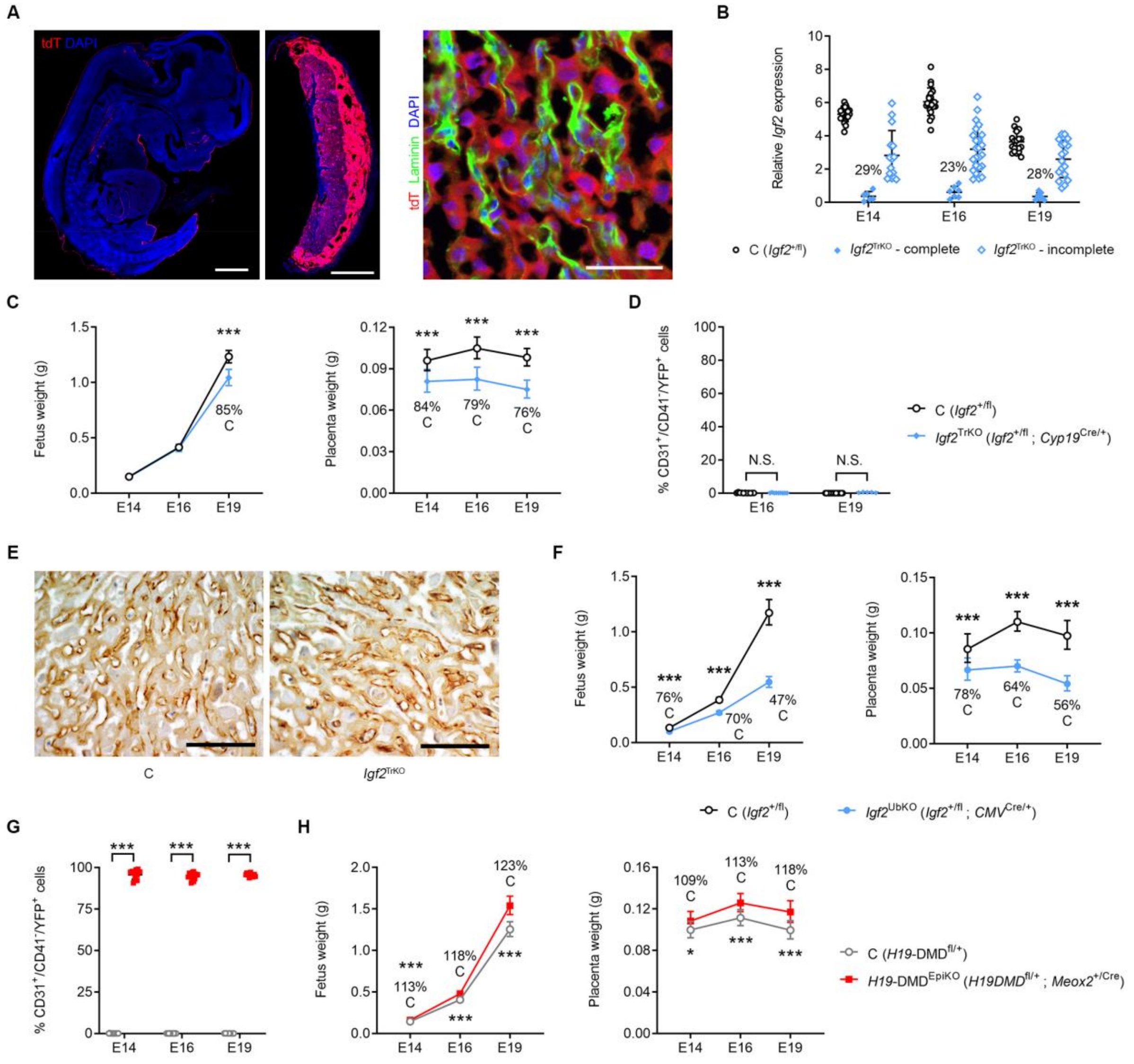

Figure S6. Specificity and efficiency of *Igf2*^{TrKO}, *Igf2*^{UbKO} and *H19*-DMD^{EpiKO} deletions. Related to Figure 4.

(**A**) Representative confocal microscopy on frozen sections from a double transgenic Ai9(RCL-tdT), *Cyp19*^{Cre} fetus and corresponding placenta, at E16, demonstrating high Cre activity (red) in placenta and weak activity in embryonic skin and eye lenses. Right panel: *Cyp19*^{Cre} is only active in the trophoblast cells in Lz, as demonstrated by lack of overlapping between the tomato protein (red) and laminin (green) expressed in FPEC. Scale bars are 1 mm (left and middle panel) or 50 µm (right panel). (**B**) Efficiency of *Igf2* deletion by *Cyp19*^{Cre} in *Igf2*^{TrKO} mutants (*Igf2*$^{+/fl}$; *Cyp19*$^{Cre/+}$) versus controls (*Igf2*$^{+/fl}$) evaluated using qRT-PCR in micro-dissected placental Jz layer (n=20–31 samples per genotype). Only 23-29% of all *Igf2*^{TrKO} mutants have high levels of deletion (>80%). (**C**) Placenta growth restriction precedes fetal growth restriction in *Igf2*^{TrKO} mutants (n=4–9 litters at each developmental stage; only mutants with >80% deletion were included in this analysis). (**D**) Flow cytometry analysis showing that *Cyp19*^{Cre} is not expressed in FPEC (note lack of YFP expression in *Igf2*^{TrKO} mutants) (n=6–21 per genotype). (**E**) Representative CD31 immunostainings in E16 control and *Igf2*^{TrKO} Lz (scale bars are 100µm) showing no impact of the deletion on FPEC numbers. (**F**) Severe fetal and placental growth

restriction in *Igf2*^{UbKO} (*Igf2*$^{+/fl}$; *CMV*$^{Cre/+}$) mutants (n=3-8 litters at each developmental stage). (**G**) Flow cytometry analysis shows that the majority (>80%) of *H19*-DMD^{EpiKO} (*Meox2*$^{+/Cre}$; *H19*-DMD$^{fl/+}$) mutant FPEC express YFP, demonstrating good efficiency of *Meox2*^{Cre} in these cells (n=9–15 per genotype). (**H**) Fetal and placental overgrowth in *H19*-DMD^{EpiKO} mutants (n=3-4 litters at each developmental stage). For all graphs, data is shown as averages or individual values ± SD in (B), (D) and (G) or 95% confidence intervals (95%CI) in (C), (F) and (H). N.S. – non-significant, * $P < 0.05$; ** $P < 0.01$; *** $P < 0.001$ calculated by two-way ANOVA plus Sidak's multiple comparisons tests in (B), (D), and (G) and mixed effects model in (C), (F) and (H).

Figure S7

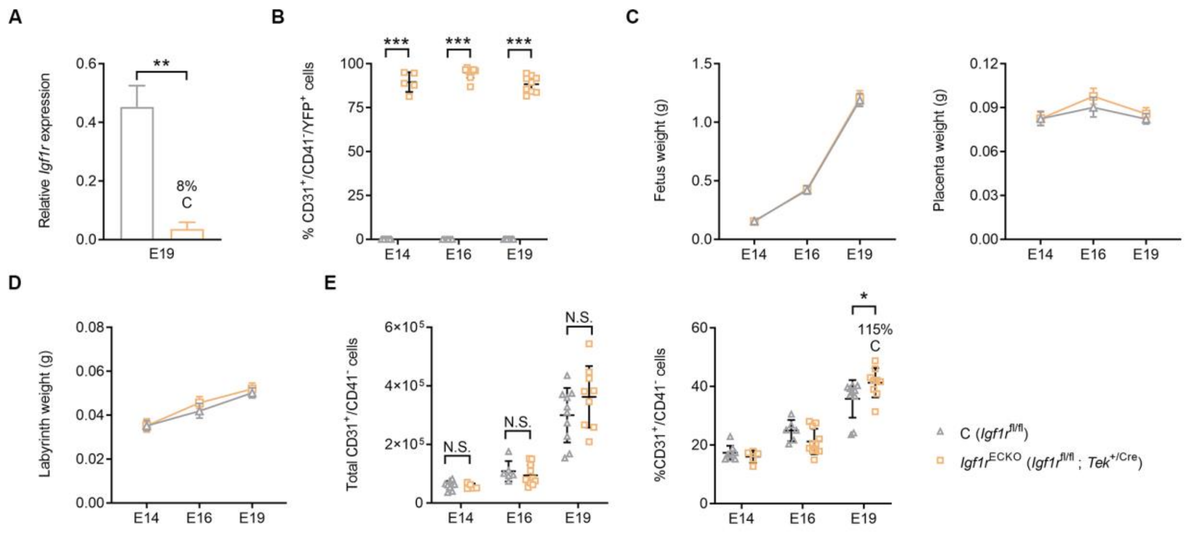

Figure S7. Conditional deletion of *Igf1r* from endothelium using *Tek*^{Cre}. Related to Figure 7.

(**A**) qRT-PCR analysis of *Igf1r* mRNA levels in primary FPEC isolated by FACS from E19 Lz of *Igf1r*^{ECKO} (*Igf1r*$^{fl/fl}$; *Tek*$^{+/Cre}$) mutants versus *Igf1r*$^{fl/fl}$ controls. (**B**) Flow cytometry analysis showing that the majority (>80%) of *Igf1r*^{ECKO} mutant FPEC express YFP, demonstrating good efficiency of *Tek2*^{Cre} in these samples (n=5–11 per genotype). Fetal, placental (**C**) and Lz (**D**) growth kinetics are not altered in *Igf1r*^{ECKO} mutants compared to controls (n=6–18 conceptuses from n=3-7 litters for each developmental stage). (**E**) Total numbers and proportions of FPEC/Lz measured by flow cytometry (n=5–11 per genotype). For all graphs, data is shown as individual values or averages ± SD in (A), (B) and (E) or 95%CI in (C) and (D); N.S. – non-significant, * $P < 0.05$, ** $P < 0.01$, *** $P < 0.001$ by Mann-Whitney tests in (A) or two-way ANOVA plus Sidak's multiple comparisons tests in (B) and (E) or mixed effects model in (C) and (D).

Table S4. Angiostatic and pro-angiogenic factors produced by feto-placental endothelial cells under the control of fetus-derived IGF2. Related to Figure 5.

Protein	Expression change	Function (cellular compartment)	Role in angiogenesis	PMID
CXCL10	Up-regulated	Cytokine (extracellular space)	angiostatic	7537965, 7540647, 8611715, 9064358, 10914483
IL15	Up-regulated	Cytokine (extracellular space)	angiostatic	28379958
THBS1	Up-regulated		angiostatic	22553494
ADAMTS1	Up-regulated	Peptidase (extracellular space)	angiostatic	12716911, 12814950, 17082774, 22776012
APCDD1	Up-regulated	Membrane-bound glycoprotein (cellular membrane)	angiostatic	29154126
IFIT2	Up-regulated	Interferon-induced protein (cytoplasm)	angiostatic	26515391
IFI16	Up-regulated	Transcription regulator (nucleus)	angiostatic	14729471, 21488755
CCL2	Up-regulated	Cytokine (extracellular space)	pro-angiogenic/ angiostatic if prolonged expression	15516694, 16888027, 23329645
EGR1	Up-regulated	Transcription regulator (nucleus)	pro-angiogenic/ angiostatic if prolonged expression	10339488, 12872165, 16818645, 27041221
KLF4	Up-regulated	Transcription regulator (nucleus)	pro-angiogenic/ angiostatic if prolonged expression	24599951, 27431648, 26823670
GDF15	Up-regulated	Growth factor (extracellular space)	pro-angiogenic	21773947, 28831101
HBEGF	Up-regulated	Growth factor (extracellular space)	pro-angiogenic	15289334, 18925469
SERPINE1	Up-regulated	Protease inhibitor (extracellular space)	pro-angiogenic	26180080
PLAT	Up-regulated	Peptidase (extracellular space)	pro-angiogenic	24601228
ISG20	Up-regulated	Exonuclease (nucleus)	pro-angiogenic	29195126
HEY2	Down-regulated	Transcription regulator (nucleus)	pro-angiogenic	15107403, 16219802, 22421041

Table S5: Mouse strains and crosses. Related to STAR Methods.

Paternal genotype	Maternal genotype	Offspring/embryo genotypes	Related to Figure
Igf2$^{+/+}$	*Igf2*$^{+/+}$	*Igf2*$^{+/+}$	Figure 1<br>Figure 2I<br>Figure 6<br>Figures S5A, S5B, S5D
Igf2$^{fl/fl}$;<br>*Rosa26YFP*-stop$^{fl/fl}$	*Meox2*$^{+/Cre}$	C (*Igf2*$^{+/fl}$;<br>*Rosa26YFP*-stop$^{fl/fl}$;<br>*Meox2*$^{+/+}$)	Figures 2B, 2C, 2D, 2J<br>Figure 3<br>Figure 4A<br>Figures 5A, 5C<br>Figures S1C, S1D, S1E, S1F, S1G, S1H<br>Figure S2A<br>Figure S4<br>Figure S5C
		Igf2^{EpiKO} (*Igf2*$^{+/fl}$;<br>*Rosa26YFP*-stop$^{+/fl}$;<br>Meox2$^{Cre/+}$)	
Igf2$^{fl/fl}$;<br>*Rosa26YFP*-stop$^{fl/fl}$	*Tek*$^{+/Cre}$	C (*Igf2*$^{+/fl}$;<br>*Rosa26YFP*-stop$^{fl/fl}$;<br>*Tek*$^{+/+}$)	Figures 2F, 2G, 2H, 2J<br>Figure 4B<br>Figure 5D<br>Figure S2B<br>Figures S3B, S3C, S3D
		Igf2^{ECKO} (*Igf2*$^{+/fl}$;<br>*Rosa26YFP*-stop$^{+/fl}$;<br>Tek$^{Cre/+}$)	
Igf2$^{fl/fl}$;<br>*Rosa26YFP*-stop$^{fl/fl}$	*Vav*$^{+/iCre}$	C (*Igf2*$^{+/fl}$;<br>*Rosa26YFP*-stop$^{fl/fl}$;<br>*Vav*$^{+/+}$)	Figures S3F, S3G, S3H, S3I
		Igf2^{HCKO} (*Igf2*$^{+/fl}$;<br>*Rosa26YFP*-stop$^{+/fl}$;<br>*Vav*$^{iCre/+}$)	
Igf2$^{+/+}$	*Meox2*$^{+/Cre}$	*Igf2*$^{+/+}$; *Meox2*$^{Cre/+}$	Figure S1I
		Igf2$^{+/+}$; *Meox2*$^{+/+}$	
Ai9(RCL-tdT)$^{fl/fl}$	*Meox2*$^{+/Cre}$	*Ai9(RCL-tdT)*$^{+/fl}$; *Meox2*$^{Cre/+}$	Figure S1B
		Ai9(RCL-tdT)$^{+/fl}$; *Meox2*$^{+/+}$	
Ai9(RCL-tdT)$^{fl/fl}$	*Tek*$^{+/Cre}$	*Ai9(RCL-tdT)*$^{+/fl}$; *Tek*$^{Cre/+}$	Figures 2E, S3A
		Ai9(RCL-tdT)$^{+/fl}$; *Tek*$^{+/+}$	
Igf2$^{+/+}$	*Tek*$^{+/Cre}$	*Igf2*$^{+/+}$; *Tek*$^{Cre/+}$	Figure S3E
		Igf2$^{+/+}$; *Tek*$^{+/+}$	
Igf2$^{fl/fl}$;<br>*Rosa26YFP*-stop$^{fl/fl}$	*Cyp19*$^{+/Cre}$	C (*Igf2*$^{+/fl}$;<br>*Rosa26YFP*-stop$^{+/fl}$<br>*Cyp19*$^{+/+}$)	Figure 4C<br>Figures S6B, S6C, S6D, S6E
		Igf2^{TrKO} (*Igf2*$^{+/fl}$;<br>*Rosa26YFP*-stop$^{+/fl}$<br>*Cyp19*$^{Cre/+}$)	
Ai9(RCL-tdT)$^{fl/fl}$	*Cyp19*$^{+/Cre}$	*Ai9(RCL-tdT)*$^{+/fl}$; *Cyp19*$^{Cre/+}$	Figure S6A
		Ai9(RCL-tdT)$^{+/fl}$; *Cyp19*$^{+/+}$	
Igf2$^{fl/fl}$;<br>*Rosa26YFP*-stop$^{fl/fl}$	*CMV*$^{+/Cre}$	C (*Igf2*$^{+/fl}$;<br>*Rosa26YFP*-stop$^{+/fl}$<br>*CMV*$^{+/+}$)	Figure 4D<br>Figure S6F
		Igf2^{UbKO} (*Igf2*$^{+/fl}$;	

		Rosa26YFP-stop$^{+/fl}$ *CMV*$^{Cre/+}$)	
Meox2$^{+/Cre}$	*H19-DMD*$^{fl/fl}$; *Rosa26YFP*-stop$^{fl/fl}$	C (*H19-DMD*$^{fl/+}$; *Rosa26YFP*-stop$^{fl/+}$; *Meox2*$^{+/+}$)	Figure 4E<br>Figures S6G, S6H
		H19-DMD^{EpiKO} (*H19-DMD*$^{fl/+}$; *Rosa26YFP*-stop$^{fl/+}$; *Meox2*$^{+/Cre}$)	
Tek$^{+/Cre}$	*Igf2r*$^{fl/fl}$; *Rosa26YFP*-stop$^{fl/fl}$	C (*Igf2r*$^{fl/+}$; *Rosa26YFP*-stop$^{fl/+}$; *Tek*$^{+/+}$)	Figures 7A, 7B, 7C, 7D, 7E, 7F, 7G
		Igf2r^{ECKO} (*Igf2r*$^{fl/+}$; *Rosa26YFP*-stop$^{fl/+}$; *Tek*$^{+/Cre}$)	
Igf1r$^{+/fl}$; *Rosa26YFP*-stop$^{+/fl}$; *Tek*$^{Cre/+}$	*Igf1r*$^{fl/fl}$; *Rosa26YFP*-stop$^{fl/fl}$	C (*Igf1r*$^{fl/fl}$; *Rosa26YFP*-stop$^{fl/fl\ or\ +/fl}$; *Tek*$^{+/+}$)	Figure S7
		Igf1r^{ECKO} (*Igf1r*$^{fl/fl}$; *Rosa26YFP*-stop$^{fl/fl\ or\ +/fl}$; *Tek*$^{+/Cre}$)	
		Het deletions (*Igf1r*$^{+/fl}$; *Rosa26YFP*-stop$^{fl/fl\ or\ +/fl}$; *Tek*$^{+/Cre}$) – not used	

Table S6. Primers used for genotyping or qRT-PCR. Related to STAR Methods.

Primers used for genotyping by PCR					
**Strain**	**Primer**	**Sequence**	**Primer**	**Sequence**	**Amplicon (bp)**
Igf2$^{fl/fl}$	F	TTACAGTTCAAAGCCACCACG	RW<br>RD	GCCAAAGAGATGAGAAGCACC<br>GCCAAACACAGTAAAAAGAAATGC	WT: 324<br>fl: 449<br>del: 384
Rosa26 ^{fl}STOP^{fl}YFP	F	TGTTATCAGTAAGGGAGCT	R-WT<br>R-fl	CACACCAGGTTAGCCTTTA<br>AAGACCGCGAAGAGTTTGT	WT: 239<br>fl: 301
Meox2-Cre	F	GGACCACCTTCTTTTGGCTTC	R-WT<br>R-Cre	AAGATGTGGAGAGTACGGGGTAG<br>CAGATCCTCCTCAGAAATCAGC	WT: 410<br>Cre: 311
Tek-Cre	F	TGTAAACAAGAGCGAGTGGA	R-WT<br>R-Cre	AGAGAATGGCGAGAAGTCAC<br>TGAGTGAACGAACCTGGTCG	WT: 240<br>Cre: 610
Vav-iCre	F-WT<br>F-Vav	ATGTCTCCAATCCTTGAACACTG<br>GACTACCTCCTGTACCTGCAAG	R-WT<br>R-Vav	GCAGTGGGAGAAATCAGAACC<br>ACTCTGATTCTGGCAATTTCGG	WT: 254<br>Cre: 329
Cyp19-Cre	F	GACCTTGCTGAGATTAGATC	R	AGAGAGAAGCATGTTTAGCTGG	Cre: 545
CMV-Cre	F	CGAGTGATGAGGTTCGCAAG	R	TGAGTGAACGAACCTGGTCG	Cre: 390
H19-DMD$^{fl/fl}$	F	CAGGCCTGTCCTCACCTGAAC	R	GCCAGCTTGCCTTGGCAACCCCTT	WT: 387<br>fl: 520
Igf2r$^{fl/fl}$	F	CCTTCCCTCCAGGCCGTTAC	R	GGTGAGGTCTCCATCTGAGTACC	WT: 225<br>fl: 259
Igf1r$^{fl/fl}$	F	CTTCCCAGCTTGCTACTCTAGG	R	CAGGCTTGCAATGAGACATGGG	WT: 124<br>fl: 220
**Primers used for qRT-PCR**					
**Gene**	**Primer**	**Sequence**	**Primer**	**Sequence**	**Amplicon (bp)**
Igf2	F	AGTCCGAGAGGGACGTGTCTA	R	CGGACTGTCTCCAGGTGTCAT	102
Angpt1	F	GAAGCAACTTCTCAACAGACA	R	TTCTTTGTGTTTTCCCTCCATT	100
Angpt2	F	CTTCTACCTCGCTGGTGAAGAG	R	GCTAAAATCACTTCCTGGTTGG	106
Tek	F	GGAGTGGAGTGAAGAACTAGG	R	GTGGAGTCAGTGATGTTGGAGA	93
Fas	F	CTGCAGACATGCTGTGGATCT	R	GCCTCCTCAGCTTTAAACTCTC	114
Ctss	F	AGAGACCCTACCCTGGACTACC	R	GATTCTTTTCCCAGATGAGACG	109
Spp1	F	ACCATGAGATTGGCAGTGATTT	R	GAGCTGCCAGAATCAGTCACTT	83
Tnnc1	F	GATCTCTTCCGCATGTTTGAC	R	TCAATGTCATCTTCCGTAATGG	107
Myocd	F	ATTCCTGTGCACACTGCTGTAA	R	GAGCTTCTTCACCTTTGGTTTG	96
Apobec1	F	GCACACCTGAGGAAACAAAGTC	R	CAGAGTGGGATCAACAGCTACA	134
Cd72	F	CCAAGGAGAACCTGAAAACTGA	R	GCACCTTTCCTGATATGGAATC	146

Dusp14	F	CTCCCTGGAAATCCTTAGCAC	R	ACCTCTGGAGCTCATGAAGATG	133
Bmp10	F	CTCTACAACAAATTCGCCACAG	R	GAGCCCATTAAAAGTGACTGGT	108
Igfbp3	F	CAGGCAGCCTAAGCACCTAC	R	GGAACTTGGAATCGGTCACTC	135
Adgre1	F	TAGCTGCTCTTCTGATACCCTC	R	CCAACATTCATCTTGTCCCCTC	145
Gcm1	F	CCGCAAGATTTACCTGAGACC	R	GAATAAGCTTCAGGGGTCCATT	98
Syna	F	AGCCCTCTCTGGACAATATTCA	R	CAAGGTGGGAGAAGATATTTGG	89
Synb	F	CAGCTGACACCCTCATTAAACA	R	ATCCAGAAATGGGAATGAAGTG	122
Slc16a1	F	TCGCAGCTTCTTTCTGTAACAC	R	TCATAGTCAGAGCTGGGTTCAA	102
Slc16a3	F	TGCAGAAGCATTATCCAGATCTAC	R	GTATCGATTGAGCATGATGAGG	99
Ly6e	F	ACATGAGAGTCTTCCTGCCTGT	R	TTCTGATCGGTACATGAGAAGC	91
Adamts1	F	CAAAGGACAGGTGCAAGCTC	R	TTGCACACAGACAGAGGTAGAG	119
Cxcl10	F	CGTCATTTTCTGCCTCATCCTG	R	TGATTTCAAGCTTCCCTATGGC	134
Thbs1	F	ATGTACCCATCCAGAGCATCTT	R	GGTTCCAAAGACAAACCTCACA	125
Edn1	F	GACATCATCTGGGTCAACACTC	R	AAGTCTTTCAAGGAACGCTTGG	86
Iigp1	F	ATGATTTGCCCTCCAGCTTTAC	R	ACTGAATATTCCCTTTTCTCATCCT	117
Cdkn1a	F	GAACATCTCAGGGCCGAAAAC	R	CACTTCAGGGTTTTCTCTTGCA	96
Hey2	F	CTGCCAAGTTAGAAAAGGCTGA	R	CTCATGAAGTCTGTGGCAAGAG	118
Igf1r	F	GTTATCCACGACGATGAGTGC	R	AGTCACCGAATCGATGGTTTTC	150
Igf2r	F	GGAAGACACCAGAACCAGACA	R	TGACACTCATCCTCTGGAAGC	103
Insr	F	GAGAGGATGTGAGACGACGG	R	AGCAGTTCTCCAGCTCATGTAG	149
Gapdh	F	ACAACTCACTCAAGATTGTCAGCA	R	ATGGCATGGACTGTGGTCAT	121
Sdha	F	TTCCGTGTGGGGAGTGTATTG	R	ATTCTGCAGCTCCAGGGTCTC	135
Pmm1	F	ATCCGGGAGAAGTTTGTGGAA	R	GCTGTCTTCATCCAGGCTGTC	144
Ppia	F	AAGGGTTCCTCCTTTCACAGAA	R	GATGCCAGGACCTGTATGCTT	146

Table S7. Conditions used for immunostainings. Related to STAR Methods.

Staining	Antigen retrieval	Blocking	Primary antibody	Secondary antibody
IGF2	Digestion with 1% pronase (Protease from Streptomyces griseus, Sigma – P6911) in 1xPBS for 10 min at 37°C	15% Donkey serum (Sigma – D9663) in PBS	Goat anti-human IGF2 (1:50, R&D systems AF-292) overnight at 4°C	AF488 Donkey anti-goat (1:200, Jackson ImmunoResearch – 705-546-147), one hour at room temperature (RT)
IGF2R	Boiling for 20 min in Tris-EDTA buffer (10 mM Tris, 1 mM EDTA, 0.05% Tween 20, pH 9.0,)	Animal-free blocking solution (Vector – SP-5030)	Rabbit anti-IGF2R (1:400, Cell Signaling 14364) overnight at 4°C	AF594 Donkey anti-rabbit (1:200, Jackson ImmunoResearch 711-546-152), one hour at RT
YFP	Autoclaving for 15 min at 121°C in citric acid buffer (10 mM citric acid, pH 6.0, 0.05% Tween 20)	5% Donkey serum (Sigma – D9663) in PBS	Goat anti-GFP (1:200, Abcam – ab6673) overnight at 4°C	AF488 Donkey anti-goat (1:200, Jackson ImmunoResearch – 705-546-147), one hour at RT
CD31 (immune-histochemistry)	Boiling for 30 min in citric acid buffer (10 mM citric acid, pH 6.0, 0.05% Tween 20)	- 3% H2O2 solution (peroxidase inactivation) 30 min at RT;<br>- 10% Goat serum (Sigma – G9023) and 1% BSA in PBS	Rabbit anti-CD31 (1:50, Abcam – ab28364) overnight at 4°C	Goat anti-Rabbit IgG, biotinylated (1:1000, Abcam – ab6720), one hour at room temperature, then Streptavidin-horse radish peroxidase (1:250 Rockland S000-03), one hour at room temperature, then DAB (Dako – K3468), 3-20 minutes at RT
CD31 (immune-fluorescence – assay 1)	Boiling for 30 min in citric acid buffer (10 mM citric acid, pH 6.0, 0.05% Tween 20)	15% Donkey serum (Sigma – D9663) in PBS	Rabbit anti-CD31 (1:50, Abcam – ab28364) overnight at 4°C	AF594 Donkey anti-rabbit (1:200, Jackson ImmunoResearch 711-546-152), one hour at RT
CD31 (immune-fluorescence – assay 2)	Boiling for 20 min in Tris-EDTA buffer (10 mM Tris, 1 mM EDTA, 0.05% Tween 20, pH 9.0,)	Animal-free blocking solution (Vector – SP-5030)	Goat anti-CD31 (1:20, R&D – AF3628) overnight at 4°C	NL557-conjugated Donkey Anti-Goat (1:200, R&D – NL001), one hour at RT
F4/80	Heat-induced antigen retrieval in Target Retrieval Solution (pH=6) – Dako S236984-2	- Bloxall (peroxidase) Blocking Solution – Vector Labs SP-6000;<br>- Animal-Free Blocker - Vector Labs SP-5030	Rat anti-Mouse F4/80 (1:20, [CI:A3-1] – Bio-Rad MCA497) 1 hour at RT	- Rabbit anti-Rat IgG (H+L) (1:250, Bethyl A110-322A) 1 hour at room temperature;<br>- Anti-Rabbit HRP (ImmPress – Vector Labs MP-7451) 30 min at RT;<br>- DAB (ImmPact DAB Kit - Vector Labs SK-4105)
MCT1	Proteinase K digestion (Dako – S3020) for 3 minutes at room temperature	15% Donkey serum (Sigma – D9663) in PBS	Chicken anti-MCT1 (1:200, Merk Millipore – AB1286-I) overnight at 4°C	AF488 Donkey anti-chicken (1:200, Jackson ImmunoResearch – 703-546-155), one hour at RT

MCT4	Proteinase K digestion (Dako – S3020) for 3 minutes at room temperature	15% Donkey serum (Sigma – D9663) in PBS	Rabbit anti-MCT4 (1:500, Merck Millipore – AB3314P) overnight at 4°C	AF488 Donkey anti-rabbit (1:200, Jackson ImmunoResearch – 711-546-152), one hour at RT
Laminin	Proteinase K digestion (Dako – S3020) for 3 minutes at room temperature	15% Donkey serum (Sigma – D9663) in PBS	Rabbit anti-laminin (1:500, Dako – Z0097) overnight at 4°C	AF488 Donkey anti-rabbit (1:200, Jackson ImmunoResearch – 711-546-152), one hour at RT
Lectin	Digestion with 0.04% pepsin (Sigma – 10108057001) in 0.01M HCl, for 10min at 37°C	- 3% methanol (peroxidase inactivation) for 10min at RT;<br>- 2% bovine serum albumin, 1% skimmed dry milk and 0.1% Tween20, for 15min at RT	biotinylated lectin (1:250 isolectin B4, B-1205, Vector Laboratories) for 90min at 37°C	horseradish peroxide-conjugated streptavidin (1:500 Rockland Immunochemicals S000-03 for 60min at RT) followed by DAB (Sigma D3939) for 10min at RT
Cytokeratin	Digestion with 0.04% pepsin (Sigma – 10108057001) in 0.01M HCl, for 10min at 37°C	- 3% methanol (peroxidase inactivation) for 10min at RT;<br>- 2% bovine serum albumin, 1% skimmed dry milk and 0.1% Tween20, for 15min at RT	Rabbit anti-pan cytokeratin (1:75 Novus Biologicals nb600-579) overnight at 4°C	alkaline phosphatase-conjugated goat anti-rabbit (1:500 Abcam ab6722) for 60min at RT followed by NBT/BCIP containing levimasole to block endogenous phosphatase (Thermo Fisher Scientific 34070) for 10min at RT
EPCAM (frozen sections)	None	15% Donkey serum (Sigma – D9663) in PBS	Rat anti-mouse CD326/Epcam Clone G8.8 (1:50, BD Biosciences 552370) overnight at 4°C	AF594 Donkey anti-rat (1:250, Thermo Fisher Scientific A-21209) one hour at room temperature
